# Phylogeography, colonization and population history of the Midas cichlid species complex (*Amphilophus *spp.) in the Nicaraguan crater lakes

**DOI:** 10.1186/1471-2148-10-326

**Published:** 2010-10-26

**Authors:** Marta Barluenga, Axel Meyer

**Affiliations:** 1 Lehrstuhl für Zoologie und Evolutionsbiologie, Department of Biology, University of Konstanz, Universitätsstrasse 10, 78457 Konstanz, Germany; 2 Museo Nacional de Ciencias Naturales CSIC, José Gutiérrez Abascal 2, 28006 Madrid, Spain

## Abstract

**Background:**

Elucidation of the mechanisms driving speciation requires detailed knowledge about the phylogenetic relationships and phylogeography of the incipient species within their entire ranges as well as their colonization history. The Midas cichlid species complex *Amphilophus *spp. has been proven to be a powerful model system for the study of ecological specialization, sexual selection and the mechanisms of sympatric speciation. Here we present a comprehensive and integrative phylogeographic analysis of the complete Midas Cichlid species complex in Nicaragua (> 2000 individuals) covering the entire distributional range, using two types of molecular markers (the mitochondrial DNA control region and 15 microsatellites). We investigated the majority of known lake populations of this species complex and reconstructed their colonization history in order to distinguish between alternative speciation scenarios.

**Results:**

We found that the large lakes contain older and more diverse Midas Cichlid populations, while all crater lakes hold younger and genetically less variable species assemblages. The large lakes appear to have repeatedly acted as source populations for all crater lakes, and our data indicate that faunal exchange among crater lakes is extremely unlikely. Despite their very recent (often only a few thousand years old) and common origin from the two large Nicaraguan lakes, all crater lake Midas Cichlid radiations underwent independent, but parallel, evolution, and comprise distinct genetic units. Indeed several of these crater lakes contain multiple genetically distinct incipient species that most likely arose through sympatric speciation. Several crater lake radiations can be traced back to a single ancestral line, but some appear to have more than one founding lineage. The timing of the colonization(s) of each crater lake differs, although most of them occurred more (probably much more) recently than 20,000 years ago.

**Conclusion:**

The genetic differentiation of the crater lake populations is directly related to the number of founding lineages, but independent of the timing of colonization. Interestingly, levels of phenotypic differentiation, and speciation events, appeared independent of both factors.

## Background

Since the application of molecular techniques gained widespread acceptance among evolutionary biologists it became possible to elucidate the mechanisms that are responsible for the emergence of novel species with more rigor than before [1-5]. Despite considerable advances in the field, a hotly debated question remains whether speciation always requires geographic separation or instead can evolve in spite of gene flow [4,6-12]. Recent studies conclude that although allopatric speciation predominates (*e.g.*, [4]), speciation with gene flow (sympatric and parapatric) is theoretically plausible (for review see *e.g. *[9]). Albeit still low, the number of empirical studies strongly supporting a scenario of sympatric and parapatric speciation is growing [9,11,13-23]. This has shifted the debate away from "if" and towards "how" and "how often" speciation with gene flow occurs in nature, and what the ecological and genetic conditions are under which sympatric and parapatric speciation are most likely to occur [9,24].

The study of biogeography and phylogeography can provide baseline information on the question as to whether speciation requires complete geographic isolation or not, by revealing contemporary and historical gene flow among populations and incipient species [25]. One problem of speciation research is that biological, ecological and biogeographic conditions at the time of the origination of species may have changed over time, and possibly be different today. Hence, the biotic conditions and geographic circumstances that might have influenced speciation mechanisms and events might often be obscured by subsequent evolutionary events [4,9,26]. For example, species that evolved in allopatry might become sympatric in their distribution subsequent to their origin (*e.g.*, [27]). One approach towards a better understanding of speciation is to study incipient species, lineages that are in the process of splitting [4]. In order to make inferences about the mode, speed and biogeographic correlates or causes of any given speciation event, it is mandatory to accumulate knowledge about the phylogeography and evolutionary history of the involved species and populations [26,28,29]. However, speciation studies are rarely combined with comprehensive phylogeographic knowledge.

Cichlids (Family Cichlidae) are tropical freshwater fishes occurring in southern and Central America, Africa, Madagascar and India. They are one of the best models for the study of biological diversification, since this family of fish comprises both the fastest rates of evolution (*e.g.*, [14,30-36]) and the most diverse adaptive radiations known [33,37-43]. The East African Great Lakes Victoria, Malawi and Tanganyika - the hotspots of their biodiversity - alone contain more than 1,500 endemic species [40,41,44,45], and have therefore been the focus of numerous studies. It has repeatedly been questioned whether the hundreds of endemic species in each of the East African lakes arose through ecological speciation in allopatric settings alone, or whether it is necessary to invoke somewhat less traditional mechanisms of speciation to explain the origin of the cichlids' unique diversity, such as sexual selection, or microallopatric, parapatric, or even sympatric diversification [38,41,46-48]. The elucidation of the mechanisms of speciation in East African cichlid assemblages is complicated by the huge dimensions of the lakes and their cichlid fish radiations, their habitat diversity and the difficulty in reconstructing species relationships, particularly among the extremely young species of lakes Victoria and Malawi [30-32,49].

Cichlids have also radiated albeit to a lesser extent in smaller-scale lake environments. Two well-known examples of such radiations are the Cameroonian [18] and the Nicaraguan [14,50] crater lake cichlids. These settings provide better grounds for testing alternative speciation modes, due to the general lack of barriers to gene flow. Evidence for sympatric speciation has repeatedly been described in these crater lake cichlid systems [14,18,19]. In this study we aim to provide a comprehensive phylogeographic context to the Nicaraguan case studies of sympatric speciation [14,51-54]. This very young cichlid radiation has already contributed towards a deeper understanding of the evolutionary processes generating cichlid diversification and is likely to do so in future studies. In order to be able to rule out alternative speciation hypotheses in the future, we provide here comprehensive knowledge about its population history and the phylogeographic circumstances under which repeated sympatric speciation occurred.

**Figure 1:**
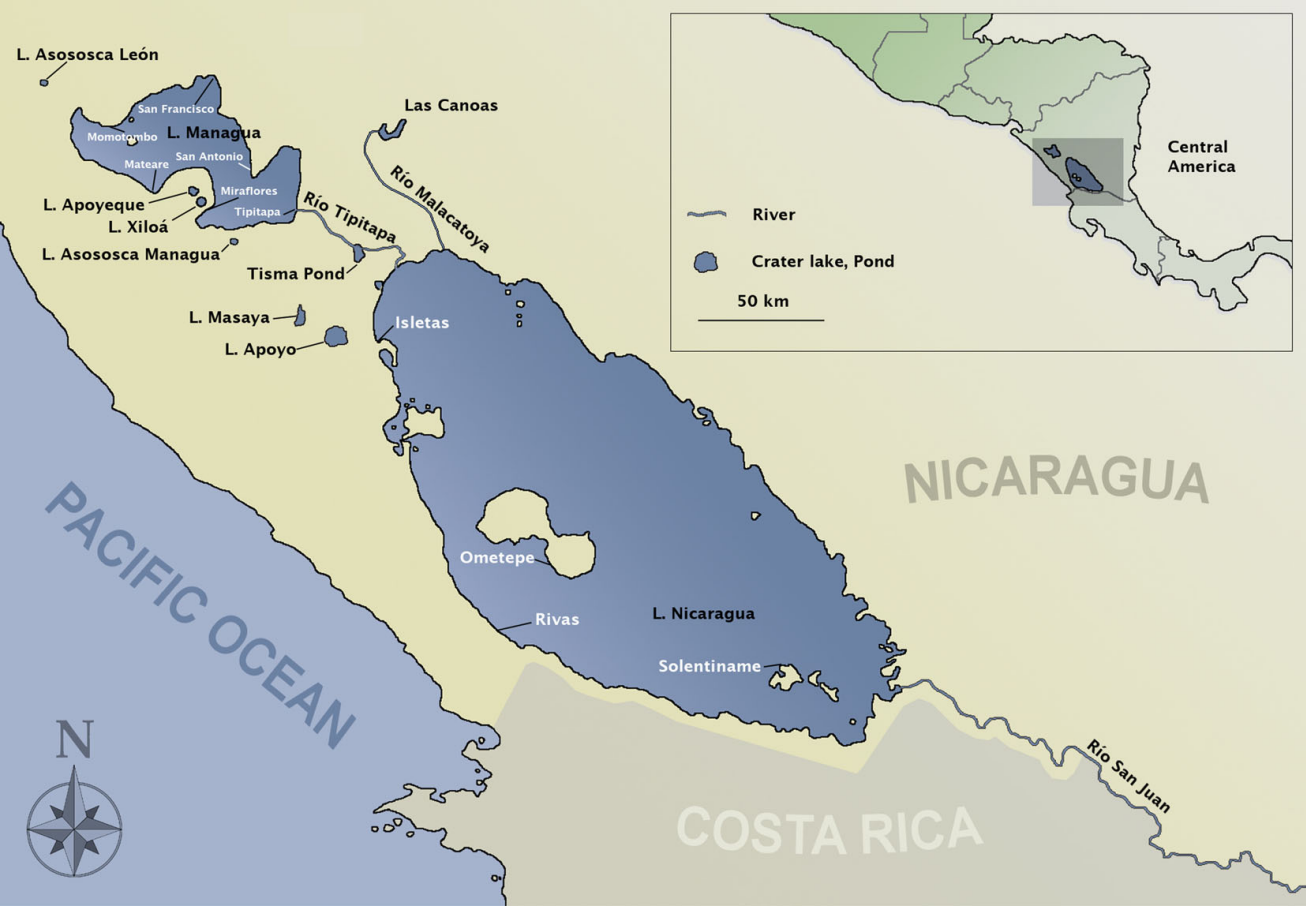
**Map of the Pacific coast of Nicaragua and Costa Rica**. Labeled localities correspond to sampling sites. Fish were collected from several sites in the large Nicaraguan lakes (Managua, Nicaragua), and from several volcanic crater lakes (Asososca León, Apoyeque, Xiloá, Asososca Managua, Masaya, Apoyo), Tisma Pond, Las Canoas reservoir, and the rivers Tipitapa, Malacatoya and San Juan.

Species of the *Amphilophus *radiation exist in an group of crater lakes that are located on the western part of Nicaragua in Central America right on the Pacific Ring of Fire, as well as in the Large Nicaraguan Lakes, Managua and Nicaragua [55-57]; Figure 1; Table 1). Fish from this group are also found in tributaries around the lakes and some rivers in Costa Rica where they are considered rare [57,58]. Initially only two species were described for the species complex, a generalist high bodied form widespread in the area (*Cichlasoma citrinellum *- now *Amphilophus citrinellus*), and a specialist thick-lipped form restricted to the large lakes Managua and Nicaragua (*C. labiatum*, now *A. labiatus*, [59,60]). A third species endemic to the crater Lake Apoyo was described more than one hundred years later, in 1976 (*C. zaliosum*, now *A. zaliosus *[56]). Several more species have since been very recently described in this polymorphic and polychromatic complex in the more thoroughly studied crater lakes Xiloá and Apoyo [14,50,52,56,61,62]. However, the taxonomy of this species complex will still need to be worked out in full, by combining ecological, morphological and genetic data also from populations of the less well-studied crater lakes. But, it seems save to say that several more species await description - depending on one's view on species. As Seth Meek already remarked in 1907 [63] about *C. citrinellus *"of all the species in these lakes, this one is by far the most variable. I made repeated efforts to divide this material listed below in from two a half-dozen or more species, but in all cases I was unable to find any tangible constant character to define them. To regard them as more than one species meant only to limit the number by the material at hand, and so I have lumped them all in one." Later that page (p. 123) he goes on: "It is possible that more than one species should be recognized here, and no doubt such will some day be the case, especially if some enthusiastic student of fishes has at his command far less amount of material than I have had the opportunity to examine."

**Table 1:** Age, dimensions and number of fish species described in the sampled Nicaraguan lakes.

	**Lake Managua**	**Lake Nicaragua**	**Crater Lake Asososca León**	**Crater Lake Apoyeque**	**Crater Lake Xiloá**	**Crater Lake Asososca Managua**	**Crater Lake Masaya**	**Crater Lake Apoyo**
	
Age	> 500000	> 500000	-	< 10000	10000	< 10000	100000-25000	< 23000
Surface (km^2^)	1050	7740	0.81	2.5	3.75	0.73	8.38	21.1
Average depth (m)	12.4	15-20	17.2	52	60	54.3	41.7	142
Maximum depth (m)	43	50	35	110	88.5	91	72.5	> 200
No. cichlid species	14(1)	16(3)	2(1)	2	9*	2	6	3*(2)
No. fish species	26	> 40	4	3	15	3	10	8

In parenthesis are the number of introduced African tilapias (*Oreochromis aureus *in Lake Managua, *O. aureus*, *O. mossambicus *and *O. niloticus *in Lake Nicaragua; *O. mossmabicus *in crater Lake Asossoca León; *O. aureus *and *O. niloticus *in crater Lake Apoyo; McKaye *et a*l. 1995; Waid *et a*l. 1999; McCrary *et al. *2007). Asterisks show the lakes for which more species of the Midas Cichlid species complex have been proposed.

The Midas cichlid species complex, as a consequence of its extensive variation in color and morphology, and its particular geographic distribution, is an especially interesting model system for the study of the mechanisms and the underlying biogeographic settings of speciation. To be able to distinguish between alternative evolutionary scenarios - *e.g.*, whether particular body plans or trophic strategies evolved only once or multiple times, or whether given lineages are the consequence of single or repeated colonization events within confined water bodies - it is compulsory to have detailed knowledge of the phylogeography, population history and colonization history of the species complex throughout its range (see [64,65]). To this end we first aimed to collect the entire diversity of phenotypic forms and species present in each water body and then investigated the phylogeographic patterns of the entire Midas cichlid complex in Nicaragua, focusing on the populations from the crater lakes, several of which are likely to have been and still are undergoing *in situ *diversification. We also collected data on the populations from the large lakes, the most likely source population of the newly formed and much younger volcanic calderas, including data from surrounding rivers.

Previous studies have partially reconstructed the phylogeography of the Midas Cichlid species complex in some of the lakes mostly based on mitochondrial DNA sequences [52,66,67] Geiger *et al. *[67] have just published a phylogeographic study of this species complex based on AFLP markers on a sample of *ca*. 100 fish from 9 water bodies, covering the majority of the area of distribution. Here, using over 2000 fish samples and two types of molecular markers (the complete sequence of the mitochondrial control region and 15 microsatellites) we reconstruct the phylogeography of the entire Midas cichlid complex. We first describe the phenotypic diversity encountered in each lake. We assign individuals to populations based on nuclear genetic data to test for the genetic cohesiveness of each lake/crater lake population. We then focus on the Large Nicaraguan lakes, which represent the source populations, because they contain the largest, oldest and most stable populations, and investigate the genetic diversity and structure of their species and populations. Following this, we investigate the age and colonization history of each crater lake, and estimate the timing, source and size of the colonization events. We discuss the relative importance of the colonizing population size *versus *the timing of colonization, and relate it to present levels of intralacustrine phenotypic differentiation. We also discuss the apparent disconnection found between current genetic and phenotypic diversity within lakes. Later we will discuss how our results compare with those of the study by Geiger *et al. *[67]. We finally test the hypothesis that isolated water bodies have undergone independent evolution, regardless of their very young history and their degree of phenotypic differentiation.

## Results

### Samples and Phenotypic diversity

The number of samples obtained in each of the studied water bodies differed which was by differences in accessibility and abundance. Localities from which we were able to collect less that 15 samples where not used for population genetic approaches. We found diverse body plans and color types in all studied lakes. In the large lakes we found high-bodied *A. citrinellus *with cryptic dark *normal *and conspicuous *gold *coloration (see [52]) in all sampled sites (< 15 samples in Mateare and Momotombo). We also collected thick-lipped *A. labiatus *individuals from both large lakes, with both normal and gold colorations, always associated with rocky habitats. Within Lake Managua *A. labiatus *was found only in two localities Miraflores (< 15 samples) and Momotombo. In Lake Nicaragua *A. labiatus *was observed at all sampled localities (but >15 samples were only collected in Isletas in Granada where this species was highly abundant). In Tisma we found both normal and gold *A. citrinellus*. In crater lakes Asososca Managua and Asososca Leon we found mostly high-bodied *A. citrinellus *forms, but also a few elongated individuals (< 15 per lake; all with normal coloration in the former lake and all normal but one gold in the latter, see Additional file 1, Table S1). In crater Lake Apoyeque all fish had normal coloration and most fish were high-bodied *A. citrinellus *type, but a few individuals had thick lips (< 15). In crater Lake Xiloá we found two types of high-bodied individuals, one with yellowish coloration breeding at smaller sizes and living in shallower habitats, and another type with both normal coloration (most abundant) and gold coloration individuals, which breed at bigger sizes and inhabit deeper habitats. We also found an elongated form living in the open water with both normal and gold coloration. These three forms have been described as new species (*Amphilophus amarillo, A. xiloaensis *and *A. sagittae *respectively; [61]). A thick-lipped form has also been seen in this lake [50], but although we have observed them while diving we collected no representatives. In crater Lake Masaya we found mostly high-bodied normal *A. citrinelus *fish, but also gold, and thick-lipped individuals (for both <15). In crater Lake Apoyo, similarly to Xiloá, we found two types of high-bodied fish, one breeding at smaller sizes and living in shallower habitats, and another breeding at bigger sizes and inhabiting deeper habitats. These two morphs have been described as new species (*A. astorquii *and *A. chancho *respectively [62]); we also collected an elongated form living in the open water - *A. zaliosus*. Another species has been described in this lake (*A. flaveolus*) similar to the small high-bodied form but with yellowish coloration. We collected a few individuals that responded to this description (Lorenzo J. Lopez Pérez pers. com.) (< 15). A detailed list of all specimens included in this study, voucher identifications and their sampling localities is provided in Additional file 1, Table S1.

### Descriptive statistics and genetic diversity

The 15 microsatellite loci analyzed (Additional file 1, Table S2) contained high levels of polymorphism. The number of alleles per locus ranged between 3 (Acit 6) and 41 (BurtKit), and the number of alleles per lake and species ranged between 64 (crater Lake Apoyeque) and 246 (*A. citrinellus*, Lake Nicaragua). Details on descriptive statistics and genetic diversity indexes averaged across loci are shown in Table 2. The highest genetic diversity measured as both gene diversity (Hs) and allelic richness was found in the populations from Lake Nicaragua (both *A. citrinellus *and *A. labiatus*) followed by the populations in Lake Managua and Tisma Pond. Genetic diversity was significantly higher in the large lakes and Tisma Pond than in the crater lakes (Hs, 0.754 *vs*. 0.596, *P *= 0.012; allelic richness, 11.219 *vs*. 6.708, *P *= 0.002). There was no evidence for systematic scoring errors according to MICRO-CHECKER. Linkage disequilibrium between pairs of loci was non-significant for every comparison. Genotype distributions were generally in accordance with expected Hardy-Weinberg proportions, and only 19 out of 180 population-locus combinations showed significant deviations, but did not consistently involve the same loci.

**Table 2:** List of number of individuals per lake and species and summary statistics of mtDNA and microsatellites (averaged across 15 loci).

	**Nicaragua**		**Managua**								
	***A. citrinellus***	***A. labiatus***	***A. citrinellus***	***A. labiatus***	**Tisma Pond**	**Asososca León**	**Lake Apoyeque**	**Lake Xiloá**	**Asososca Managua**	**Lake Masaya**	**Lake Apoyo**
	
No. Individuals	345	268	265	32	56	40	90	522	44	149	248
**mtDNA sequences**											
Haplotype richness	17.81	12.66	15.09	14	14.86	6.75	1.33	3.22	4.77	3.42	5.29
% private haplotypes	44%	76%	40%	43%	25%	100%	2%	66%	75%	39%	100%
Distance (no. mutations)	16	13	13	10	10	5	3	10	4	9	8
Haplotype diversity	0.963 ± 0.006	0.954 ± 0.008	0.942 ± 0.008	0.867 ± 0.005	0.883 ± 0.036	0.822 ± 0.040	0.128 ± 0.0047	0.824 ± 0.021	0.588 ± 0.074	0.807 ± 0.021	0.736 ± 0.028
Nucleotide diversity	0.005 ± 0.003	0.005 ± 0.003	0.005 ± 0.003	0.003 ± 0.002	0.005 ± 0.003	0.003 ± 0.002	0.0001 ± 0.0001	0.002 ± 0.001	0.001 ± 0.001	0.003 ± 0.002	0.002 ± 0.001
Tajima's D	-2.524**	-2.102**	-2.201**	-1.845*	-1.161^ns^	-0.504^ns^	-1.364*	-1.899*	0.041^ns^	-1.488*	-2.181**
**microsatellites**											
Allelic richness	12.81	11.51	10.89	10.06	10.83	4.10	6.02	9.13	5.67	9.36	7.45
Gene diversity	0.780	0.757	0.744	0.717	0.746	0.421	0.580	0.668	0.524	0.737	0.590
Fis	0.035	0.009	0.048	0.048	0.043	0.152	0.038	0.117	0.044	0.033	0.142

Haplotype and allelic richness are calculated haplotypes measures the maximum distance significant. based on the minimum population sample size (32 individuals). Distance between in mutations within each sample. Probability values: *P < 0.05, **P < 0.001, ns, non significant.

### Clustering analyses and population genetic differentiation

**Figure 2:**
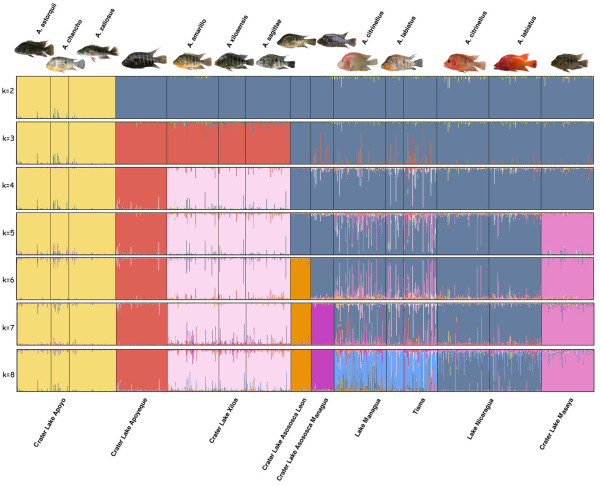
**Bayesian population assignment test based on 15 microsatellite loci with the software STRUCTURE**. Seven to eight genetically distinct populations are uncovered that might be considered to be species.

We estimated the number of genetically distinct populations contained in our Midas Cichlid species group by applying a Bayesian model-based clustering algorithm, assuming a fixed number of populations as implemented in STRUCTURE[68]. This analysis revealed that all individuals within water bodies formed genetically homogeneous groups based on multilocus nuclear markers (Figure 2). Importantly, all different species and morphotypes within the large and crater lakes were genetically more similar to other species or morphs within the same lake than to phenotypically similar forms in other water bodies. It is important to note that thick-lipped forms from the crater lakes (Apoyeque, Masaya and Xiloá) were genetically indistinguishable from the other fish within those lakes. The best log-likelihood probability was found associated with k = 7 (see a plot with all the log-likelihood probabilities in Additional file 2, Figure S1a). To further evaluate this result we applied a clustering algorithm assuming a variable number of populations as implemented in STRUCTURAMA. This analysis found that the most likely number of populations was 4 (Additional file 2, Table S3a). We plotted clustering groups from k = 2-8 in Figure 2. Each step from k = 2 to k = 8 gradually differentiated better and better among the different lakes in the following sequence: k = 2, the three species in crater Lake Apoyo, which formed a genetically homogeneous cluster, from the rest; k = 3, crater Lake Apoyo, crater Lake Apoyeque together with the three species in crater Lake Xiloá, from the rest; k = 4, crater Lake Apoyo, crater Lake Apoyeque, all three species in crater Lake Xiloá, from the rest; k = 5, crater Lake Apoyo, crater Lake Apoyeque, crater Lake Xiloá, crater Lake Masaya, from the rest; k = 6, crater Lake Apoyo, crater Lake Apoyeque, crater Lake Xiloá, crater Lake Masaya, crater Lake Asososca León, from the rest; k = 7, crater Lake Apoyo, crater Lake Apoyeque, crater Lake Xiloá, crater Lake Masaya, crater Lake Asososca León crater Lake Asososca Managua from, the rest, and k = 8 crater Lake Apoyo, crater Lake Apoyeque, crater Lake Xiloá, crater Lake Masaya, crater Lake Asososca León, crater Lake Asososca Managua, Lake Managua (*A. citrinellus *and *A. labiatus*) plus Tisma Pond, and Lake Nicaragua (*A. citrinellus *and *A. labiatus*). We did a further clustering analysis restricted to the populations within the large lakes, and found no differentiation between the two species, *A. citrinellus *and *A. labiatus *within any of the two lakes. STRUCTURE revealed k = 2 (Additional file 2, Figure S1b) separating all fish within each of the two large lakes (Tisma Pond clustering together with the fish from Lake Managua), and STRUCTURAMA confirmed two genetically distinct populations (Additional file 2, Table S3b, see also k = 2 in Additional file 2, Figure S2).

**Figure 3:**
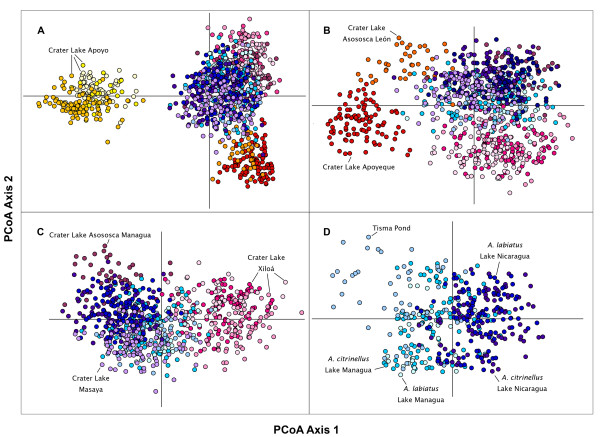
**Plot of the two first axes of the Principal coordinate analyses (PCoA)**. Each circle represents a single individual and colors reflect species and lake of origin. **A**. Analysis including all sampled populations (PCoA1 = 44.71%, PCoA2 = 16.54%). **B**. Analysis excluding the populations from crater Lake Apoyo (PCoA1 = 27.49%, PCoA2 = 20.39%). **C**. Analysis excluding samples from crater lakes Apoyo, Apoyeque and Asososca León (PCoA1 = 24.67%, PCoA2 = 19.65%). **D**. Analysis including the samples from the large lakes and Tisma Pond (PCoA1 = 24.04%, PCoA2 = 19.03%).

We also performed a series of principal correspondence analyses (PCoA) to investigate the relative position of each lake population within a multidimensional space (Figure 3). The result of this analysis was largely congruent with the clustering of STRUCTURE. We first included all populations, and the first principal coordinate axis (Figure 3a; PCoA1 = 44.71%, *F_ST _*= 0.071, *P *= 0.01) clearly differentiated the species in crater Lake Apoyo from all other populations, as did the previous clustering method. PCoA2 (16.54%, *F_ST _*= 0.025, *P *= 0.12) separated, although not significantly, the species within crater Lake Apoyo, and populations in crater lakes Apoyeque and Asososca León from the rest (Figure 3a). The second analysis (Figure 3b) was performed without the samples from crater Lake Apoyo, and differentiated the populations from crater lakes Apoyeque and Asososca León from the other populations along PCoA1 (27.49%, *F_ST _*= 0.033, *P *= 0.001). PCoA2 (20.39%, *F_ST _*= 0.023, *P *= 0.001) separated these two populations. Hence, the third analysis (Figure 3c) was performed without samples from the crater lakes Apoyo, Apoyeque and Asososca León. This analysis PCoA1 (24.67%, *F_ST _*= 0.021, *P *= 0.001) distinguished most clearly the species in crater Lake Xiloá (which showed some overlap with individuals from Lake anagua) and crater Lake Masaya (which showed some overlap with Lake Nicaragua) from the rest, and less clearly the samples from crater Lake Asososca Managua. PCoA2 (19.65%, *F_ST _*= 0.016, *P *= 0.001) separated crater lakes Xiloá and Masaya from crater Lake Asososca Managua. The last analysis included only the samples from the two large lakes in Nicaragua (both *A. citrinellus *and *A. labiatus*) and Tisma Pond. PCoA1 (24.04%, *F_ST _*= 0.014, *P *= 0.001) discriminated between the two lakes but revealed some degree of overlap, and clumped the samples from Tisma Pond together with those of Lake Managua (PCoA2 = 19.03%, *F_ST _*= 0.005, *P *= 1.00; Figure 3d). As in the previous clustering analysis, within the two large lakes the two species *A. citrinellus *and *A. labiatus *were not clearly differentiated.

F-statistics revealed that populations of the Midas Cichlid species complex from all lakes were generally significantly differentiated from each other with both types of molecular markers (average mtDNA *Φ_ST _*= 0.23; microsatellites *F_ST _*= 0.16). The few exceptions from this general finding involved the fish from the large lakes, fish from Tisma Pond and fish from the River San Juan (see Table 3, Additional file 2, Table S4). Fish from Tisma Pond cannot be genetically distinguished from *A. citrinellus *in Lake Managua, to which it is temporarily connected. Fish from the River San Juan cannot be distinguished from *A. citrinellus *in Lake Nicaragua, to which is permanently connected. Interestingly, fish from Las Canoas, a water body geographically connected to Lake Nicaragua but today separated by a dam, were genetically clearly differentiated from all other populations (Additional file 2, Table S4); no single mtDNA haplotype was shared between Las Canoas and fish in any other water body. All identified species within crater lakes are genetically differentiated from each other supporting their taxonomic assignment as different species (Table 3). Geographically distant populations within the large lakes showed some degree of genetic differentiation as well. Within Lake Managua, populations of *A. citrinellus *collected from different sites (Figure 1, names in white) were genetically indistinguishable, but *A. labiatus *collected in Momotombo were consistently genetically differentiated from *A. citrinellus *from all other localities with both types of molecular markers (see Additional file 2, Table S4a). Within Lake Nicaragua, geographically distant populations of both *A. citrinellus *and *A. labiatus *were not consistently differentiated, although some pairwise comparisons clearly differed (Additional file 2, Table S4b). The comparison of the two species in the best-sampled population, the Isletas, revealed significant differences.

**Table 3:** Matrix of pairwise *F*-statistics between pairs of lakes and species.

	**Nicaragua**		**Managua**												
	***A. citrinellus***	***A. labiatus***	***A. citrinellus***	***A. labiatus***	**Tisma**	**Asososca León**	**Apoyeque**	***A. amarillo***	**Xiloá *A. xiloaensis***	***A. sagittae***	**Masaya**	**Asososca Managua**	***A. astorquii***	**Apoyo *A. chancho***	***A. zaliosus***
	
Lake Nicaragua															
*A. citrinellus*	0.000	0.015**	0.120**	0.027^ns^	0.115**	0.367**	0.072**	0.047**	0.032**	0.084**	0.075**	0.276**	0.253**	0.278**	0.293**
*A. labiatus*	0.009**	0.000	0.140**	0.023^ns^	0.124**	0.380**	0.060**	0.039**	0.034**	0.072**	0.067**	0.266**	0.286**	0.308**	0.337**
Lake Managua															
*A. citrinellus*	0.029**	0.023**	0.000	0.143**	-0.004^ns^	0.347**	0.234**	0.184**	0.154**	0.223**	0.217**	0.357**	0.319**	0.319**	0.344**
*A. labiatus*	0.055**	0.044**	0.021**	0.000	0.152*	0.540**	0.134**	0.031^ns^	0.033^ns^	0.107**	0.090**	0.460**	0.438**	0.431**	0.595**
Tisma Pond	0.017**	0.011**	-0.015^ns^	0.013*	0.000	0.400**	0.352**	0.215**	0.180**	0.281**	0.248**	0.424**	0.414**	0.378**	0.488**
Lake Asososca León	0.177**	0.193**	0.192**	0.261	0.218**	0.000	0.788**	0.598**	0.565**	0.662**	0.569**	0.716**	0.655**	0.571**	0.735**
Lake Apoyeque	0.154**	0.159**	0.130**	0.180	0.134**	0.253**	0.000	0.052**	0.143**	0.146**	0.124**	0.728**	0.642**	0.686**	0.814**
Lake Xiloá															
*A. amarillo*	0.106**	0.092**	0.066**	0.082	0.061**	0.278**	0.167**	0.000	0.031**	0.062**	0.082**	0.443**	0.461**	0.488**	0.578**
*A. xiloaensis*	0.089**	0.078**	0.059**	0.084	0.060**	0.287**	0.184**	0.016**	0.000	0.080**	0.093**	0.447**	0.426**	0.442**	0.543**
*A. sagittae*	0.130**	0.118**	0.078**	0.103	0.078**	0.289**	0.171**	0.048**	0.055**	0.000	0.140**	0.526**	0.537**	0.566**	0.665**
Lake Masaya															
	0.065**	0.067**	0.044**	0.071	0.027**	0.207**	0.142**	0.108**	0.100**	0.119**	0.000	0.415**	0.448**	0.478**	0.538**
Lake Asososca Managua	0.124**	0.118**	0.125**	0.186	0.145**	0.341**	0.266**	0.195**	0.191**	0.245**	0.171**	0.000	0.657**	0.645**	0.782**
Lake Apoyo															
*A. astorquii*	0.215**	0.225**	0.228**	0.270	0.230**	0.410**	0.355**	0.293**	0.297**	0.324	0.231**	0.375**	0.000	0.109**	0.089**
*A. chancho*	0.185**	0.201**	0.208**	0.247	0.220**	0.403**	0.345**	0.290**	0.288**	0.320**	0.214**	0.361**	0.074**	0.000	0.129**
*A. zaliosus*	0.253**	0.269**	0.274**	0.313	0.296**	0.457**	0.382**	0.348**	0.363**	0.369**	0.276**	0.433**	0.138**	0.127**	0.000

Below the diagonal are the data from the microsatellites and above the diagonal the data from mtDNA sequences. Probability values: *P < 0.05, **P < 0.001, ns, non significant.

### Phylogenetic reconstruction and demographic analyses

**Figure 4:**
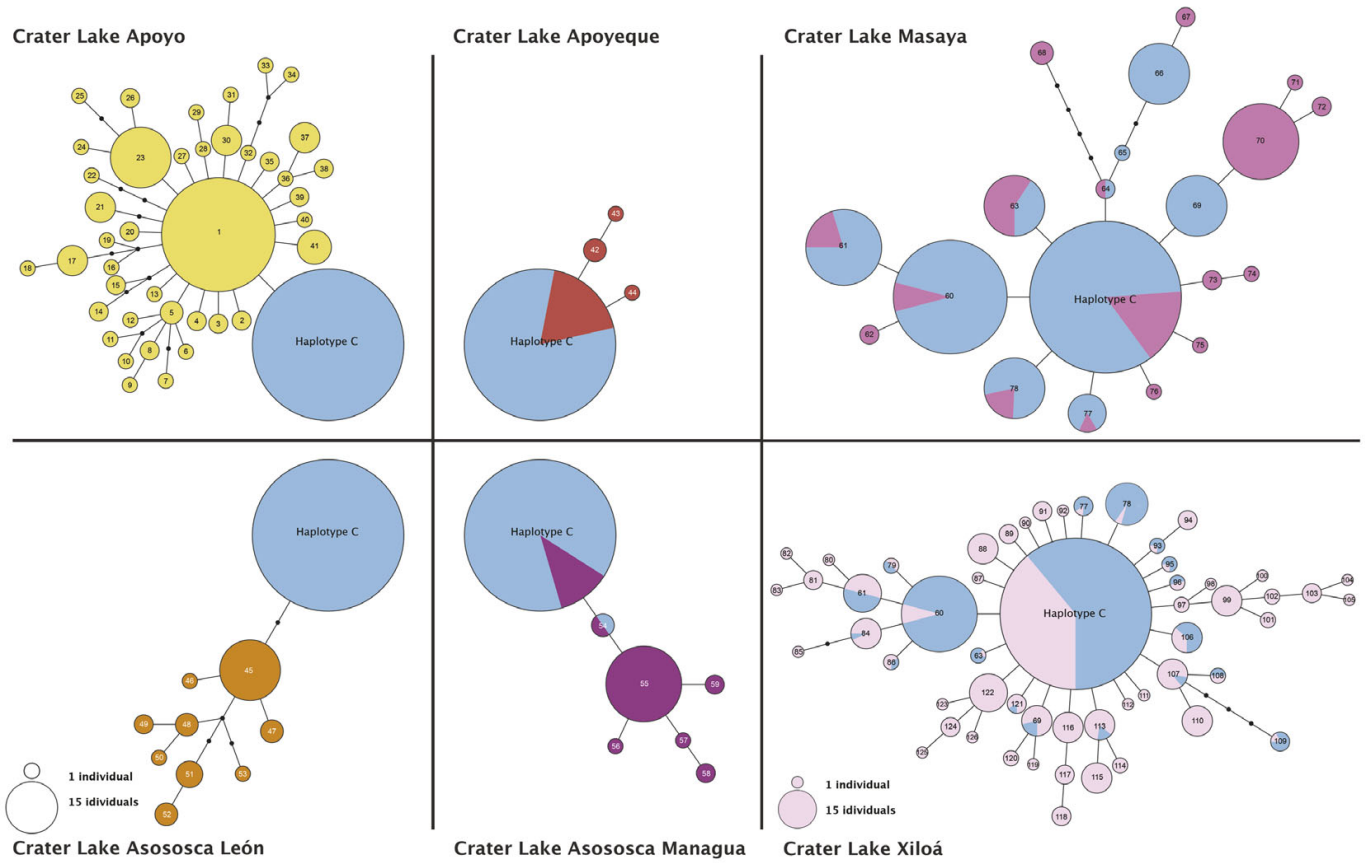
**Unrooted haplotype networks of the complete mtDNA control region of cichlids of the Midas Cichlid species complex from six crater lakes in Nicaragua**. Circles represent unique DNA sequences, and their size reflects the number of individuals sharing a particular haplotype (see scale; note that scale is different for crater Lake Xiloá). Colors refer to different lakes, light blue represents in each case fish from elsewhere outside the given crater lake. Connections between haplotypes represent mutational steps. The central haplotype 'C', is the most common haplotype that is found in the large lakes and some of the crater lake populations.

Among the 2173 fish included in this study we found 512 different mitochondrial haplotypes. MtDNA haplotypes differed from each other by an average of 3.5 and a maximum of 17 mutations. The number of haplotypes, proportion of private alleles, maximum number of mutations and nucleotide diversity found in each lake is shown in Table 2. A haplotype network of all unique mtDNA sequences had a star-like structure, with a central most abundant haplotype (haplotype C) found in almost all populations and localities in the central position. Due to the difficulties in depicting such large number of haplotypes, only the networks for each of the six crater lakes we investigated are shown (Figure 4), and since all species within crater lakes clustered together in the Bayesian assignment test using nuclear markers, in this analyses they were depicted with a single crater lake color. Only two crater lakes, Apoyo (including all described species) and Asososca León do not contain individuals with the most common mtDNA haplotype 'C'. The samples from the large lakes Nicaragua and Managua (including both species *A. citrinellus *and *A. labiatus*) and Tisma Pond, contained the largest amount of genetic diversity with the largest maximum distance between haplotypes (see Table 2). These lakes shared many haplotypes, but they had also many private ones. Crater Lake Apoyeque contained the smallest mtDNA diversity with only three private haplotypes found that were separated each only by single mutations, with almost all of the 90 Apoyeque individuals investigated here sharing the central haplotype C (note that within a much smaller sample size, only 15 individuals, Geiger *et al. *[67] only found the central haplotyope C and none of the private mtDNA haplotypes). Crater lakes Masaya and Xiloa both had a large number of different haplotypes, several private and several shared with both large lakes. Crater Lake Asososca Managua shared the haplotype C, and one more haplotype (54) with two samples from Lake Managua and Tisma respectively. Crater lakes Asososca Leon and Apoyo did not share any haplotype with any other locality and are the only two crater lakes discovered to be monophyletic as shown by mtDNA. Detailed information about the mtDNA haplotype of each individual used is shown in Additional file 1, TableS1.

**Figure 5:**
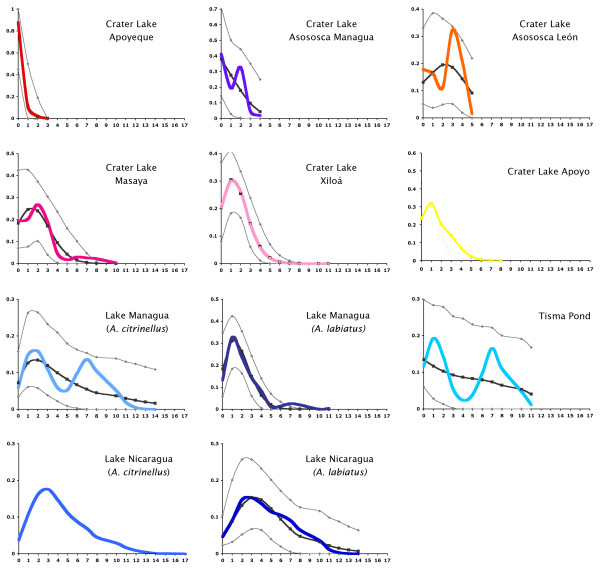
**Mismatch analysis showing the inferred demographic histories of individuals from each species in each of the Nicaraguan lakes**. Colored lines represent observed data, black lines represent the best-fit model, and in grey are the upper and lower boundaries.

A mismatch analysis was performed to compare the demographic history of the Midas Cichlid lineages in the different lakes of Nicaragua (Figure 5); the associated parameters are summarized in Table 4. All populations but those of *A. citrinellus *from the large Lake Nicaragua and fish in crater Lake Apoyo followed a model of sudden expansion. The oldest expansions with the largest mismatch values (mismatch mean values > 4) were detected in the two large lakes. We found signatures of extremely recent expansions in crater Lake Apoyeque (mismatch mean value < 1). Significant Tajima's D with very negative values were found for all populations but those from Tisma Pond, Asososca León and Asososca Managua (Table 2), showing deviation from neutrality, possibly as a consequence of the expansion of populations, although these result could also be explained by selective sweeps.

**Table 4:** Mismatch analysis estimated parameters

	**Lake Nicaragua**		**Lake Managua**								
	***A. citrinellus***	***A. labiatus***	***A. citrinellus***	***A. labiatus***	**Tisma Pond**	**Lake Asososca León**	**Lake Apoyeque**	**Lake Xiloá**	**Lake Asososca Managua**	**Lake Masaya**	**Lake Apoyo**
	
Mean no. of differences											1.594
τ	-	4.167	2.852	1.781							0.836
θ_0_	-	0.739	2.167	0.034	0.316	0.156	0.001	0.066	0.040	0.060	0.006
θ_1_	-	27457.3	54099.3	73519.2	10366.5	30380.4	16999.9	58317.6	50978.8	43333.2	81522.500
SSD	0.055**	0.002^ns^	0.016^ns^	0.005^ns^	0.027^ns^	0.038^ns^	0.0001^ns^	0.0001^ns^	0.033^ns^	0.007^ns^	0.057**
Raggedness index	0.012^ns^	0.008^ns^	0.022^ns^	0.064^ns^	0.034^ns^	0.098^ns^	0.594^ns^	0.034^ns^	0.145^ns^	0.032^ns^	0.031^ns^

τ is the moment estimator of the age of the expansion; θ_0 _and θ_1 _are the mutation parameters before and after the expansion respectively; SSD is the test of the validity of a stepwise expansion model based on the sum of square deviations between the observed and the expected mismatch, and the significance of the test is estimated with a parametric bootstrap approach, and the same method is used to test the significance of the Raggedness index (probability values: *p < 0.05, **p < 0.001, ns = non-significant).

## Discussion

The Midas Cichlid species complex has gained notoriety as a textbook example for rapid phenotypic diversity, explosive rates of speciation and as an example for speciation in sympatry. It is well recognized as being phenotypically highly polymorphic despite its very young age [35,52,56,57,63,69,70]. Based on this phenotypic diversity several incipient species have been described [50,61,62]. Our comprehensive phylogeographic approach finds it to be genetically diverse as well, with levels of overall diversity comparable to that of the hundreds of cichlid species inhabiting the Lake Victoria region in East Africa [31,32]. All Nicaraguan lakes differed in their levels of genetic diversity. Populations in the vast and old Nicaraguan large lakes were as expected genetically most diverse (Table 2; Figure 5). They contain the oldest, and most diverse fauna (Table 1). Crater lake populations were typically genetically much less diverse (Figure 5, Table 2) and certainly much younger. The crater lake populations differed considerably from each other just as the crater lakes vary in size, age and degree of isolation (Table 1). Remarkably, in spite of being genetically rather depauperate (crater Lake Apoyeque is the extreme case where only three different mtDNA haplotypes were found), the Midas cichlids in some crater lakes are phenotypically diverse, and even contain novel phenotypes, which in several cases are genetically differentiated (Table 3) and most likely constitute endemic incipient species. There was no obvious relationship between the number of morphs or species described for a particular crater lake, and its genetic diversity. Fish from the two large lakes appear to have repeatedly acted as source populations of the newly formed and much younger volcanic calderas. Lake Managua most likely seeded the northern crater lakes, being physically connected in the past to some of them (e.g. Lake Xiloá), and L. Nicaragua the southern ones, as suggested by levels of genetic similarity, relative position in the multilocus multidimensional space (Figure 3), and geographical proximity. Most interestingly, despite their recent and common origin, all crater lake Midas Cichlid populations underwent independent evolution and have unique genetic signatures (Figure 2). All Nicaraguan lakes are genetically differentiated from each other, and all different morphs or incipient species described within each lake are genetically more similar to each other than to any other fish in any other lake. Some remarkable similar phenotypes are found across some of the crate lake species assemblages that most likely arose independently in parallel.

### The Large Nicaraguan lakes: the source populations

The large Nicaraguan lakes, Managua and Nicaragua contain the most genetically diverse Midas Cichlid populations, which have experienced the oldest demographic expansions 3-4 mutations ago (Figure 5; Table 2; Table 4). Previous estimations date the earliest expansion of Midas cichlids in the Nicaraguan lakes *ca*. 100,000 years ago [52] Geological data confirm that the basin of the large Nicaraguan lakes originated less than 1 Mya [70,71], and fish are estimated to first have colonized the area only about 500,000 years ago [70]. Despite the relatively shallowness of the area, demographic analyses show that the large Nicaraguan lakes probably remained a relatively stable environment for fish populations over time, owing to their large dimensions and relatively homogeneous environment. The large Nicaraguan lakes are the most likely historical source of the Midas Cichlid species complex. The members of this species complex are rare in rivers, although its closest relatives (*A. hogaboomorus*, *A. centrarchus *or *A. trimaculatus *[72]) are riverine species, as probably was their ancestor. The Midas Cichlid ancestor must have adapted early to the lacustrine environment where the species is now most abundant.

Historically, the two large Nicaraguan lakes formed a single water body that separated in the late Pleistocene [55,73]. Today they remain connected via the River Tipitapa, partially subterranean, but with periodical water exchange. Accordingly, the fish from the two large lakes are genetically relatively similar (Figure 2, Figure 3), although significant population differentiation between them does exist (Figure 2, Table 3). The permanent pond on the connecting river, Tisma, contains a fish population genetically indistinguishable from those from Lake Managua, at the inflow of the river, but they are genetically differentiated from the fish close to the outflow into Lake Nicaragua (Table 3, Additional file 2, Figure S2). Therefore, the contemporary exchange of fish between these lakes appears not to be very extensive. Lake Nicaragua drains in the south into River San Juan. Accordingly, *A. citrinellus *from the upper Rio San Juan are genetically indistinguishable from those in the lake (Additional file 2, Table 4). The River Malacatoya drains into the North of Lake Nicaragua, but the fish from the reservoir in this river (Las Canoas) are genetically markedly differentiated from *A. citrinellus *in Lake Nicaragua, as well as in any other water body, indicating absence of contemporary fish exchange (Table 3).

#### Allopatric differentiation

The sheer size of the large Nicaraguan lakes, they are the largest in Central America, might favor the divergence of geographically distant populations within lakes. Cichlid species widespread in the large African lakes often show allopatric diversification, even forming allopatric local species in some instances (see review in [43]). In both large Nicaraguan lakes, populations of *A. citrinellus *separated by several kilometers showed some genetic differentiation, but this result was not consistently supported by the different molecular markers employed (Additional file 2, Table S4). Thus, the genetic differentiation of allopatric populations of *A. citrinellus *within the large lakes is not very pronounced in our sample, possibly because of large effective population sizes and the persistence of shared ancestral polymorphisms. However, relatively high levels of recent gene flow among populations within this lake could also explain this finding, since no barrier to gene flow other than distance exists. Similarly, phenotypic differences among allopatric populations are not pronounced (unpubl. data), probably due to habitat similarity and local adaptations to similar environmental conditions. Some hidden diversity (both genetic and phenotypic) could still remain undiscovered due to the relative turbidity of the large lakes, although this possibility seems unlikely because of the extensive work in the area over the years.

#### Sympatric differentiation

Two species of the Midas Cichlid complex co-occur in sympatry in the large Nicaraguan lakes, the widespread and common *A. citrinellus*, and the seemingly more specialized and only locally abundant thick-lipped species *A. labiatus*. These two species are morphologically quite distinct, which suggests that they also are ecologically segregated, although ecological studies are still lacking. *Amphilophus labiatus *have very characteristic fleshy lips [56], a character also common in other African cichlid species [39], and more elongated snout [74], traits interpreted as adaptations for feeding on invertebrates and crustaceans between crannies, sealing the substrate and sucking the food items [56,75]. The elongated snout is suggested to be an additional adaptation for increasing the power of suction [74]. In agreement with this hypothesized foraging behavior, we found *A. labiatus*, as other fleshy-lipped cichlids, linked to the presence of rocky habitats-which is also reflected in our sample collection, while *A. citrinellus *was mostly found on sandy substrates in the large lakes [76].

*Amphilophus labiatus *was collected from several sites in Lake Nicaragua, and populations across the lake showed some allopatric differentiation. Sympatric populations of the two species showed genetic differentiation, but the Bayesian assignment tests did not discriminate between them (Additional file 2, Figure S2). In Lake Managua *A. labiatus *is less abundant, since there are also fewer rocks, and we did not find it to be coexisting in sympatry with *A. citrinellus*. For this study only one sampling site provided sufficient samples (Momotombo), and *A. labiatus *from this site was consistently genetically significantly different from all *A. citrinellus *populations. However, since no sympatric sample of *A. citrinellus *from this site could be collected, it is not possible to distinguish between species segregation due to ecological adaptation or allopatric differentiation.

In summary, although thick-lipped species are common in cichlid radiations, and *A. citrinellus *and *A. labiatus *are morphologically and ecologically clearly differentiated, they remain genetically surprisingly indistinguishable (Additional file 2, Figure S2). This is possibly due to the persistence of ancestral shared polymorphisms. Pronounced phenotypic plasticity can be ruled out since in sympatric settings genetic differences are often found and lips persist also in the laboratory, when fish are feed on the same diets (A.M. pers. obs.). Also in the laboratory thick-lipped fish prefer to mate with thick-lipped fish over fish with small lips (A.M. pers. obs.) further supporting their status as two described species. It would appear that the morphological innovation of fleshy lips evolved very rapidly and multiple times independently in different geographic locations. This idea is supported by the presence of thick-lipped forms in some of the Nicaraguan crater lakes (such as Masaya, Xiloá and Apoyeque - although in low frequencies and genetically undifferentiated from other forms within those lakes) and in other cichlid radiations in the African lakes. Future research on additional morphological characters such as body shape, trophic structures or coloration and genetic differentiation might lead to future descriptions of several thick-lipped species in those crater lakes.

### Colonization history of the crater lakes

An interesting result of this study is that despite the recent origin of most crater lakes (< 20,000 years), the Midas Cichlid populations in each of them had their own characteristic genetic signature, clearly differentiated from any other population in the area (Figure 2), as well as its own array of phenotypes and species ([50,74], Barluenga *et al. *unpubl.). This genetic distinction has also been found using AFLP data on a smaller sample of Midas cichlids, corroborating the robustness of this result [67]. Subsets of the same source populations from the large lakes seeded all crater lakes. Potential heterogeneity within the source populations due to unconnected colonization events, coupled with the independent evolution occurring in each crater lake, have resulted in very differentiated crater lake populations. This is genetically reflected in the abundance of private mtDNA haplotypes in each lake (Table 2), their unique microsatellite allele composition (Figure 2; Figure 3) and, ultimately, the evolution of endemic crater lake species in some cases [14,50,51,56,62,77]. For example, in crater Lake Xiloá also a new sardine and a new shrimp species have been described [77]. In several crater lakes several new species that belong to the Midas Cichlid species complex have been described more recently [50,61,62], and we here report genetic differentiation among several of them.

The unique genetic signature of each crater lake population appears to be influenced by the combination of two main factors, the age of colonization (recent *vs*. old), but also the number of founding lineages (single or multiple colonizations, and/or founding populations large or small). Other factors likely to influence genetic composition are demographic events after colonization.

#### Timing of colonization

We inferred the time of colonization of the different crater lakes from the estimates of major demographic expansions they experienced within these areas (Figure 5). If there were major demographic events after colonization (*e.g.*, volcanic activity or newer colonizations) our estimations would correspond to establishment of the recent population rather than colonization *per se*. Older populations in the Midas Cichlid system are characterized by higher numbers of private alleles and a distinct allelic composition, but not necessarily by pronounced phenotypic differentiation. Younger populations are typically genetically undifferentiated from the source population, although in some cases contain novel morphotypes, that have not been described in the large source lakes. Remarkably, we find a pronounced disconnection between genetic and phenotypic diversity. According to the demographic analyses based on mtDNA sequences, the oldest colonization was most likely in crater Lake Asososca León, which expanded within the lake about 3-4 mutations ago (Figure 5). It is probably a relatively old lake, although its exact geological age is unknown. This lake contains a relatively large number of unique mtDNA haplotypes (see also [67]) separated by up to six mutations from any haplotype that is found in any of the other lakes (Figure 4). Interestingly and in spite of a relatively old age and diverse genetic signature, phenotypic diversity does not appear to be pronounced. Although not comparatively analyzed here, most individuals belonged to the typical high-bodied morphotype, with only a few more elongated individuals. The estimates of population expansion of the Midas Cichlid species complexes of crater lakes Masaya and Apoyo (each of them containing at least three different incipient species; [14,62,78], pers. obs.) are younger, about 1-2 mutations ago. This would fit with the known age of Lake Apoyo, dated at less than 20,000 years. A population expansion about one mutation ago was detected for Lake Xiloá (with three to five described species; [50]), which agrees with the records of the last volcanic activity in the area (INETER, http://www.ineter.gob.ni/). Population expansions occurring less than one mutation ago were consistently found in Asososca Managua (with mostly high bodied *A. citrinellus *individuals, but several more elongated) and Apoyeque (two morphs described [78,79]), lineages and lakes that could be just a few thousand of years old. Clearly, in less, possibly even much less than 10,000 years [79], the species complexes in each of the crater lakes in Nicaragua expanded and diversified to the small-scale radiations that are now evident from most of these isolated lakes.

#### Number of founding lineages

Genetic diversity values in some crater lakes strikingly contrast their very recent origin (according to volcanic activity reports, [80]; INETER, http://www.ineter.gob.ni/), and young estimated demographic history. This is the case for crater lakes Xiloá and Masaya (Table 2; Figure 4), which also have relatively high number of fish species (Table 1). Some of this genetic variation possibly did not evolve *in situ *in these lakes, due to the recency of their multiple colonization(s) (see also Figure 5). These two lakes could have been temporarily connected to the large lakes, when they would have acquired a large portion of the source fauna's genetic diversity (including diverse Midas cichlids), which would also explain the extensive faunal correspondence among these lakes crater lakes and the large Nicaraguan lakes. No data about physical connections exist for crater Lake Masaya, and geological and geographic evidence make this possibility rather unlikely, leaving the presence of this diversity (including polymorphic and polychromatic Midas cichlid species) unresolved. Unfortunately, crater Lake Masaya is highly polluted, since the city of Masaya dumps its waste and waste water untreated into it, severely hampering detailed study of its fauna due to health concerns. Geological reports confirm that crater Lake Xiloá was originally connected to Lake Managua [81] supporting our interpretation of the origin of its genetic diversity. These two lakes (Xiloá and Managua) separated when the water level dropped as Lake Nicaragua began to drain into the Atlantic Ocean through the San Juan river [77]. The fossil record also indicates that the original fauna of Lake Xiloá was more diverse than it is nowadays [77]. It is argued that the stringent physical conditions of this crater lake (very high concentration of salts in the water) might have caused the disappearance of some species, as well as the evolution of local endemism [77]. The high levels of polymorphism in the Midas cichlids of this crater lake suggests that *in situ *diversification might have taken place in the lake during its short history independent from Lake Managua [50]. This study demonstrate all Midas cichlids from crater Lake Xiloá are genetically more similar to each other than to any other fish in the area (Figure 2; see also [51]).

Both crater lakes Asososca Managua and Apoyeque have a signature typical of small and genetically homogeneous colonizations, since all mtDNA haplotypes are connected to each other by single mutations, and the amount of genetic diversity is muted. Alternatively, those populations might have experienced a bottleneck due to volcanic activity after colonization. The time since their colonization is estimated to be particularly short (especially crater Lake Apoyeque was probably colonized in historic times; [79]) which is reflected in very small overall distances between haplotypes. The age of the present day populations in both lakes could be much younger than 10,000 or in the case of Apoyeque even only 1000 years. Geological data report the last eruption of Volcano Apoyeque < 2,000 years ago (INETER, http://www.ineter.gob.ni/; [82]). Periodic fish kills are reported to occur still today for some of the crater lakes due to remaining volcanic activity, and the Apoyeque crater has even been reported to have been active in historic times, less than 150 years ago. Remarkably, despite its very recent origin, the crater Lake Apoyeque contains a polymorphic Midas Cichlid population, with normal and thick-lipped forms [78,79], The colonization of these two lakes could have been caused by exceptional natural phenomena, such as a hurricane or a tropical storm that could have resulted in fish rains. However, it has been speculated that indigenous human populations might have stocked some of the crater lakes with fishes as well [73]. It should be noted, however, that the genetic signatures of the lakes argue against this possibility (multiple human stockings at least) since most of the crater lakes appear to be both monophyletic and show unique population expansion signals.

Crater Lake Apoyo also has a signature of a small and genetically homogeneous colonization and no single mtDNA haplotype is shared with any other lake (*i.e.*, all mtDNA haplotypes are private to the lake; Figure 4). The genetic signature of the Midas Cichlid species complex that is endemic to Lake Apoyo is the most derived as revealed by all genetic analyses (Table 3; Figure 2; Figure 3), uncovering a very strong founder effect [7] during the colonization, followed by a pronounced expansion event, about two mutational steps ago. It has already been shown that *A. zaliosus*, an endemic species adapted to open water, originated in this lake and evolved sympatrically with the ancestral Midas cichlid population within the lake [14,56]. Here we show evidence of differentiation of up to three species within this crater lake, all forming a genetically homogeneous group (both with mitochondrial and nuclear markers). This confirms the scenario of sympatric speciation *in situ *in the lake, and make alternative evolutionary scenarios (such as the proposed multiple colonizations of the lake with introgression, or the origin of these species somewhere outside crater Lake Apoyo and secondary colonization [83]), far less parsimonious, implying a series of very unlikely extinction events (see [84]).

Making inferences about the evolutionary processes that have occurred in crater Lake Asososca León is rendered difficult since the colonization of this lake occurred a relatively long time ago compared to the other crater lakes. We show here, as we did previously also for crater Lake Apoyo [14], that all mtDNA haplotypes are private (Figure 4). This strongly suggests that the size of the colonizing population was very small, or, alternatively, that a major extinction event, or even periods of smaller population size have occurred in its history that decimated all other haplotypes that might have once existed in this crater lake. The latter explanation seems rather unlikely since there is no reason to assume that only the locally endemic mtDNA types should have survived such a catastrophic event. Another alternative possibility would be that the lake was colonized from a source other than the large lakes, *e.g.*, a river, thus explaining the lack of sharing of mtDNA haplotypes with their populations. Samples of other fish species from this lake would allow exploring this, albeit unlikely, hypothesis. Although this lake has not been thoroughly studied, no major morphological or genetic diversification among the Midas cichlids from there has been detected, despite the presence of a few more elongated individuals.

The combined effect of both timing and size of the colonizing populations will influence the genetic composition of newly formed crater lake populations, together with possible demographic events after colonization linked to volcanic activity. Although the longer ago a colonization event occurred, the more likely it is for populations to have diversified *in situ*, we find the strongest genetic differentiation among species in lakes where founder populations were small and genetically homogeneous, regardless of their age. Bottleneck effects most strongly shape the genetic structure of crater lake populations at neutral loci. Interestingly, the evolution of phenotypic differentiation does not seem to be related to genetic diversity (at least as measured by neutral markers). Most likely, differences in ecological opportunity (diversity of ecological niches, release of interspecific competition and predation, etc.) among crater lakes determine the degree of morphological, behavioral and ecological diversity contained in each system. The validation of this hypothesis would require a more formal description of all the phenotypic diversity as well as the characterization of the ecological niches contained in each lake, an effort that is still incomplete.

#### Origin of the colonization

The large lakes are the most likely source populations of all crater lake Midas cichlids. The origin of the Midas Cichlid in crater Lake Asososca León is difficult to reconstruct since it shows no obvious genetic affinities to any other studied population. Crater Lake Apoyeque has a genetic composition that would be compatible with several population sources, since the most common haplotype within the lake is also the overall central one (haplotype C); also the most common haplotype in the majority of the other lakes, including the large lakes. Its geographical position suggests that its fauna could have its origin in Lake Managua. Crater Lake Xiloá could be a potential source as well, and nuclear markers cluster these populations together (k = 3 in Figure 2), although the central haplotype 'C' is not as common among the Midas cichlids from there. The fauna of crater Lake Xiloá very likely had its origin in Lake Managua (suggested by the F-statistics and PCoA analyses; see also [67]), just as that of crater Lake Asososca Managua (which only shares haplotypes with fish from this lake, apart from the haplotype C) (Figure 2, Figure 3; Table 3). Crater Lake Masaya shows higher similarity to Lake Nicaragua (Figure 2), what is consistent with its closer geographic proximity. The Midas cichlids from crater Lake Apoyo are rather distinct from any other population, but pairwise genetic distances (Table 3) and geography suggest that it might have had its origin in Lake Nicaragua, which is also supported by recent AFLP analyses [67].

#### Neutral genetic diversity and phenotypic variation

When populations colonize new environments adaptations to the novel conditions arise. The Midas Cichlid in several lakes in Nicaragua has evolved new species adapted to different ecological niches. Novel phenotypes often appear by selection on new mutations. But this process requires waiting for new mutations to be generated. Faster alternatives are the recruitment of beneficial mutations from standing genetic variation [85], or even reshuffling of standing variation by hybridization [86]. The evolution of the Midas Cichlid in the Nicaraguan crater lakes has occurred in a relatively short period of time [14,52]. New mutations are not likely to explain all existing diversity. In addition, the repeated evolution of equivalent species makes the 'evolution of new mutations' hypothesis rather unlikely. Therefore, it is likely that standing genetic variation plays an important role here. Some crater lakes have been colonized by multiple lineages, perhaps at multiple times, which will likely maintain high standing genetic diversity, and even make possible re-shuffling of existing variation. However, some crater lake populations have clearly been colonized by severely bottlenecked lineages, which reduces the expected retained variation. Interestingly, we do not find reduced or slowed levels of adaptation and speciation in these populations, as exemplified by the radiation in crater Lake Apoyo, or even in crater Lake Apoyeque. This raises the very exciting question of how standing levels of genetic variation affect the speed and diversity attained by adaptive radiations. Preliminary transcriptome wide analyses on crater Lake Apoyo species, where genetic diversity is relatively low, revealed weak functional genomic differences between the incipient species [87], concordant with the young age of the radiation. Similar analyses on genetically richer crater lakes could provide information about the relative functional differentiation in relation to standing diversity.

## Conclusion

The Midas Cichlid species complexes from the Nicaraguan lakes are genetically and phenotypically diverse. Single lake flocks comprise in some cases homogeneous and monophyletic lineages, while in some others more diverse and polyphyletic genetic lineages possibly colonized by more than one ancestral species. The isolation of populations in the crater lake habitats combined with the different selective pressures they are exposed to (physicochemical conditions of the water, size and depth of the lakes, predation and competition pressures due to different biotic compositions, etc.), suggest that evolution *in situ *within these lakes is currently happening, making this species complex a model system for the study of parallel sympatric speciation. The differences in the diversity of ecological and morphological forms found in each lake could not simply be explained as an effect of colonization by different ancestral lineages, since the source populations for all of these crater lake species assemblages were the same: the large Nicaraguan lakes. It is remarkable that the obvious similarities in ecological specializations due to ecological opportunities - open water species such as *A. zaliosus *in Apoyo and *A. saggitae *in Xiloá, only exist in deep crater lakes - are absent from the source populations in the large lakes, and arose independently and extremely rapidly in more than one crater lake. Strong similarity can be found to the situation of the stickleback species pairs of postglacial lakes. The Midas Cichlid community in each crater lake is likely shaped by similar ecological and evolutionary forces that promote the evolution of equivalent ecological communities.

## Methods

### Sampling

Fish of the Midas Cichlid species complex were collected during the dry season (February-March) of 2001 and the wet seasons (November-December) of 2003 and 2005 with gill nets, harpooning while scuba diving, and with the help of local fishermen, from twelve different locations in Nicaragua, covering the main range in which these species are found (with the exception of a few smaller rivers and two isolated lakes, -Monte Galán and Tiscapa). The collections included the two Large Nicaraguan Lakes - L. Managua and L. Nicaragua (including samples from several distant localities in each lake, see Figure 1; Additional file 2, Table S4), and from a pond (called Tisma) on the River Tipitapa that connects these two lakes. Also seven crater lakes (from North to South: Asososca León, Apoyeque, Xiloá, Asososca Managua, Masaya and Apoyo; see Figure 1 and Table 1 for details on age size depth and faunal composition of the crater lakes) were sampled. A water reservoir on the River Malacatoya that drains into Lake Nicaragua, Las Canoas and River San Juan that drains from Lake Nicaragua into the Atlantic Ocean were sampled as well (Figure 1). All fish were photographed in a standardized position for phenotypic characterization. All major body plans found in each water body were described. Fish were sized, and fin clips and muscle samples were preserved in 90% ethanol for DNA analyses. Whole fish or fish heads were taken as voucher specimens and to characterize the trophic apparatus. A total of 2157 fish were collected (see Table 2 for sample sizes per lake).

### Microsatellite analysis

Total DNA was isolated using a proteinase *K *digestion followed by sodium chloride extraction and ethanol precipitation [88]. Individuals were genotyped with 15 microsatellite loci (eight loci were developed specifically for *Amphilophus citrinellus*: Acit1, Acit2, Acit3, Acit4, Acit6, [89]; Unh011, Unh012, Unh013, McKaye *et al. *2002; two published loci developed for other cichlid species: TmoM7, [90]; Unh002, [91]; and five newly developed loci: Abur28, Abur45, Abur82, Abur151, [92], (Genbank accession numbers: EU564224, EU564237, EU564256 and EU564292 respectively); BurtKit, Salzburger *et al. *unpubl.; see Additional file 2, Table S2), amplified with fluorescent reverse primers (HEX, FAM and NED dyes) and fragment length was analyzed with the internal size marker Genescan-500 ROX (Applied Biosystems) on an ABI 3100XL Automated Sequencer (Applied Biosystems), and with GENESCAN v.3.7 and GENOTYPER v.3.7 (Applied Biosystems) software packages. Genotypes were checked for scoring errors using MICRO-CHECKER v. 2.3 [93].

The basic descriptive statistics, number of alleles, F_IS_, and the genetic diversity indexes allelic richness and gene diversity were compiled for each population using FSTAT v.2.9.3 [94]. We compared the statistical differentiation of the genetic diversity indexes between the group formed by all the crater lakes and the group of all populations in the large lakes and Tisma Pond with FSTAT. Departure from Hardy-Weinberg equilibrium for each locus across and within populations was calculated using a test analogous to Fisher's exact tests [95] estimated with a 100,000 step, 1,000 iteration, Markov Chain Monte Carlo series of permutations, as implemented in ARLEQUIN v. 3.0 [96]. Linkage disequilibrium was tested for all possible pairs of loci in each population and globally for each pair of loci across populations with ARLEQUIN. Critical significance levels for multiple testing were corrected following the sequential Bonferroni procedure [97].

Two different approaches were used in an effort to examine the genetic clustering of the populations in the different lakes. First, the Bayesian clustering method as implemented in STRUCTURE v. 2.2 [68] was used to assess the grouping of individuals without prior information on their sampling location assuming admixture. With this analysis we aimed to test for genetic similarity within each water body. We included information about species within the better studied crater lakes Xiloá and Apoyo. This clustering method assumes a fixed number of populations *K *and calculates the posterior probability of assigning individuals to each of the populations using a variant of the Markov chain Monte Carlo (MCMC) method. Posterior probabilities were calculated in multiple runs, where each run consisted of 500,000 iterations with a burn-in period of 50,000. We set *K *between 1-15, and performed 10 replicates for each value of K. Pritchard *et al. *[68] suggest a method based upon marginal likelihoods to determine the minimal number of populations needed to explain the data. Possible limitations of this method have been suggested [98]. Therefore we also used a method implemented in the software STRUCTURAMA v.1.0 [99], where both the assignment of individuals to populations and the number of populations are treated as random variables following a Dirichlet process prior [100]. This method allows to directly estimate the number of populations, and is suggested to provide more accurate estimations of the number of populations [101]. The results of this clustering method were plotted with the software DISTRUCT v. 1.1 [102].

Second, a Principal Coordinate Analysis (PCoA) of individual diploid genotypes was performed as implemented in GENALEX v.6 [103] to visualize the two dimensional clustering of populations. Several analyses were performed by successively removing the most differentiated populations to reveal all levels of differentiation among populations (Figure 4). Tests of the significance of total inertia and individual axes inertia, as well as F_ST _differentiation per axes were calculated with 1000 permutations with PCAGEN v.1.0 [104].

In the last two analyses (PCoA and Bayesian clustering) in order to have a compensated number of samples due to software limitations, we used only a subset of the samples representing all localities and morphotypes of the fish with a maximum of 100 individuals per lake and species.

### mtDNA analyses

The complete mitochondrial control region (836 bp) was PCR amplified using published primers and reaction conditions (L-Pro-F, [105]; 12S5R, 5'-GGC GGA TAC TTG CAT GT-3') on a GeneAmp PCR System 9700 Thermocyler (Applied Biosystems). The polymerase chain reaction (PCR) products were purified using the QIAquick PCR purification kit (QIAGEN), and sequenced in both directions with the BigDye Terminator Cycle Sequencing Ready Reaction kit (Applied Biosystems). Sequencing products were analyzed on an ABI 3100 Automated Sequencer (Applied Biosystems) (see Additional file 1, Table S1 for GenBank accession numbers).

Mitochondrial DNA sequences were aligned using the software SEQUENCHER v. 4.2 (Gene Code Corporation) and verified by eye. The alignment was collapsed into the representative haplotypes with COLLAPSE v. 1.2 http://darwin.uvigo.es/software/collapse.html, but the information about the frequencies of haplotypes was preserved. Number of haplotypes, proportion of private alleles, and nucleotide diversity [106] per lake and species were calculated with ARLEQUIN. A maximum likelihood (ML) tree was constructed with PAUP* v. 4.0b10 [107]. A model of sequence evolution was chosen applying jMODELTEST v. 0.1 [108]. jMODELTEST revealed that the optimal model of molecular evolution was the unequal-frequency Kimura 3-parameter plus Gamma correction (0.68) and a proportion of invariable sites of 0.58% (K81uf+I+G). On the basis of the obtained tree, a haplotype network including geographic information and frequency of haplotypes was built (Figure 2). The ML tree was translated into an unrooted tree with maximum parsimony branch lengths in order to associate each branch with mutational steps. Gaps were incorporated in the definition of the haplotypes for the construction of the network.

A mismatch analysis was performed to estimate the demographic history of the species complex from each of the lakes (Figure 3, Table 3). The fit of the observed pairwise mismatch distributions to a sudden expansion demographic model was tested using a generalized least square procedure and by computing the raggedness index of the observed distributions [109] as implemented in ARLEQUIN. The validity of a stepwise expansion model for the data was tested using Markov Chain Monte Carlo simulations (1000 steps) with ARLEQUIN. We computed the moment estimator of the age of the expansion (τ), and the mutation parameters θ_0 _(2 μN_0_) and θ_1 _(2 μN_1_) using a parametric bootstrap approach (1000 simulations), where μ is the mutation rate and N is the female effective population size. Tajima's D [110] analysis was performed with ARLEQUIN as an additional test of population expansion.

Genetic differences among and within lakes were estimated with pairwise *F*-statistics [111] as implemented in ARLEQUIN. Critical significance levels for multiple testing were corrected following the sequential Bonferroni procedure [97].

## Authors' contributions

MB and AM designed the study and obtained the material. MB did the molecular work and the genetic analyses, and wrote a first draft of the manuscript. Both authors completed and approved the final manuscript.

## Authors' information

Marta Barluenga is an evolutionary ecologist interested in speciation and the origin of adaptive radiations. Axel Meyer is an evolutionary geneticist interested in the ecological, developmental and genomic aspects of speciation and organismal diversification.

## Supplementary Material

Additional file 1 **Supplementary Table 1**. Table with all the individuals included in the study with species identification, individual ID, lake of origin, GenBank accession numbers of mitochondrial control region sequences, and haplotype ID (see Figure 2). A citrinellus* accounts for individuals within the crater lakes that could not be assigned to any of the newly described species because of being juveniles. Click here for file

Additional file 2 **Supplementary materials**. This additional file contains information about the microsatellite primers used in the study, and additional details on the results of the clustering analyses with STRUCTURE and STRUCTURAMA, and pairwise statistics. Click here for file
