# Supplementary material for: Phylogeography, colonization and population history of the Midas cichlid species complex (Amphilophus spp.) in the Nicaraguan crater lakes
Source: BMC Evol Biol. 2010 Oct 26;10:326. doi: 10.1186/1471-2148-10-326 (PMC3087546; doi:10.1186/1471-2148-10-326)
Supplement: Additional file 1 — Supplementary Table 1. Table with all the individuals included in the study with species identification, individual ID, lake of origin, GenBank accession numbers of mitochondrial control region sequences, and haplotype ID (see Figure 2). A citrinellus* accounts for individuals within the crater lakes that could not be assigned to any of the newly described species because of being juveniles. [file 1471-2148-10-326-S1.PDF]

Table S1. List of species, sample number, lake of origin, GenBank accession numbers of mitochondrial control region sequences, and haplotype ID (see Fig. 2).
*A citrinellus** accounts for individuals within the crater lakes that could not be assigned to any of the newly described species because of being juveniles.

Species	Specimen ID	Lake	Accesion No.	Haplotype
A. zaliosus	zAyo3101	L. Apoyo	DQ229964	-
A. zaliosus	zAyo3118	L. Apoyo	DQ229965	1
A. zaliosus	zAyo3119	L. Apoyo	DQ229966	1
A. zaliosus	zAyo3122	L. Apoyo	DQ230058	23
A. zaliosus	zAyo3124	L. Apoyo	DQ230051	41
A. zaliosus	zAyo3125	L. Apoyo	DQ229969	1
A. zaliosus	zAyo3126	L. Apoyo	DQ229970	1
A. zaliosus	zAyo3129	L. Apoyo	DQ229972	1
A. zaliosus	zAyo3130	L. Apoyo	DQ229973	1
A. zaliosus	zAyo3131	L. Apoyo	DQ229974	1
A. zaliosus	zAyo3132	L. Apoyo	DQ229975	1
A. zaliosus	zAyo3135	L. Apoyo	DQ229977	1
A. zaliosus	zAyo3137	L. Apoyo	DQ229979	1
A. zaliosus	zAyo3138	L. Apoyo	DQ230052	41
A. zaliosus	zAyo3140	L. Apoyo	DQ230053	41
A. zaliosus	zAyo3141	L. Apoyo	DQ229981	1
A. zaliosus	zAyo3147	L. Apoyo	DQ230054	41
A. zaliosus	zAyo3150	L. Apoyo	DQ229985	1
A. zaliosus	zAyo3151	L. Apoyo	DQ229986	1
A. zaliosus	zAyo3153	L. Apoyo	DQ229987	1
A. zaliosus	zAyo3154	L. Apoyo	DQ229988	1
A. zaliosus	zAyo3155	L. Apoyo	DQ229989	1
A. zaliosus	zAyo3159	L. Apoyo	DQ229992	1
A. zaliosus	zAyo3160	L. Apoyo	DQ229993	-
A. zaliosus	zAyo3167	L. Apoyo	DQ229996	1
A. zaliosus	zAyo3449	L. Apoyo	DQ230055	41
A. zaliosus	zAyo3458	L. Apoyo	DQ230007	-
A. zaliosus	zAyo3459	L. Apoyo	DQ230056	41
A. zaliosus	zAyo3460	L. Apoyo	DQ230008	-
A. zaliosus	zAyo3461	L. Apoyo	DQ230009	-
A. zaliosus	zAyo3462	L. Apoyo	DQ230010	-
A. zaliosus	zAyo3463	L. Apoyo	DQ230048	30
A. zaliosus	zAyo3474	L. Apoyo	DQ230015	1
A. zaliosus	zAyo3964	L. Apoyo	DQ230057	41
A. zaliosus	zAyo3965	L. Apoyo	DQ230019	1
A. zaliosus	zAyo3968	L. Apoyo	DQ230020	1
A. zaliosus	zAyo3969	L. Apoyo	DQ230021	1
A. zaliosus	zAyo3976	L. Apoyo	DQ230027	1
A. zaliosus	zAyo5377	L. Apoyo	HM183496	1
A. zaliosus	zAyo5378	L. Apoyo	HM183627	40
A. zaliosus	zAyo5379	L. Apoyo	HM183628	41
A. zaliosus	zAyo5380	L. Apoyo	HM183581	23
A. zaliosus	zAyo5381	L. Apoyo	HM183497	1
A. zaliosus	zAyo5382	L. Apoyo	HM183629	41
A. zaliosus	zAyo5383	L. Apoyo	HM183630	41
A. zaliosus	zAyo5384	L. Apoyo	HM183498	1
A. zaliosus	zAyo5385	L. Apoyo	HM183499	1

A. zaliosus	zAyo5386	L. Apoyo	HM183631	41
A. zaliosus	zAyo5387	L. Apoyo	HM183632	41
A. zaliosus	zAyo5388	L. Apoyo	HM183500	1
A. zaliosus	zAyo5389	L. Apoyo	HM183501	1
A. zaliosus	zAyo5390	L. Apoyo	HM183502	1
A. zaliosus	zAyo5391	L. Apoyo	HM183566	14
A. zaliosus	zAyo5392	L. Apoyo	HM183633	41
A. zaliosus	zAyo5393	L. Apoyo	HM183582	23
A. zaliosus	zAyo5394	L. Apoyo	HM183503	1
A. zaliosus	zAyo5395	L. Apoyo	HM183618	35
A. zaliosus	zAyo5396	L. Apoyo	HM183504	1
A. zaliosus	zAyo5397	L. Apoyo	HM183505	1
A. zaliosus	zAyo5398	L. Apoyo	HM183506	1
A. zaliosus	zAyo5399	L. Apoyo	HM183578	21
A. zaliosus	zAyo5400	L. Apoyo	HM183507	1
A. zaliosus	zAyo5401	L. Apoyo	HM183508	1
A. zaliosus	zAyo5402	L. Apoyo	HM183509	1
A. zaliosus	zAyo5403	L. Apoyo	HM183510	1
A. zaliosus	zAyo5404	L. Apoyo	HM183511	1
A. zaliosus	zAyo5405	L. Apoyo	HM183512	1
A. zaliosus	zAyo5406	L. Apoyo	HM183513	1
A. zaliosus	zAyo5407	L. Apoyo	HM183619	35
A. zaliosus	zAyo5408	L. Apoyo	HM183514	1
A. zaliosus	zAyo5409	L. Apoyo	HM183515	1
A. zaliosus	zAyo5410	L. Apoyo	HM183634	41
A. zaliosus	zAyo5411	L. Apoyo	HM183516	1
A. zaliosus	zAyo5413	L. Apoyo	HM183517	1
A. zaliosus	zAyo5414	L. Apoyo	HM183635	41
A. zaliosus	zAyo5415	L. Apoyo	HM183518	1
A. zaliosus	zAyo5416	L. Apoyo	HM183636	41
A. zaliosus	zAyo5417	L. Apoyo	HM183567	14
A. zaliosus	zAyo5418	L. Apoyo	HM183577	20
A. zaliosus	zAyo5419	L. Apoyo	HM183579	21
A. zaliosus	zAyo5421	L. Apoyo	HM183519	1
A. zaliosus	zAyo5422	L. Apoyo	HM183520	1
A. zaliosus	zAyo5423	L. Apoyo	HM183521	1
A. zaliosus	zAyo5424	L. Apoyo	HM183522	1
A. zaliosus	zAyo5689	L. Apoyo	HM183523	1
A. zaliosus	zAyo5697	L. Apoyo	HM183524	1
A. zaliosus	zAyo5709	L. Apoyo	HM183525	1
A. zaliosus	zAyo5710	L. Apoyo	HM183568	14
A. zaliosus	zAyo5711	L. Apoyo	HM183608	30
A. zaliosus	zAyo5712	L. Apoyo	HM183526	1
A. astorquii	cAyo3128	L. Apoyo	DQ230040	15
A. astorquii	cAyo3139	L. Apoyo	DQ229980	1
A. astorquii	cAyo3156	L. Apoyo	DQ230060	23
A. astorquii	cAyo3440	L. Apoyo	DQ230031	5
A. astorquii	cAyo3448	L. Apoyo	DQ230061	23
A. astorquii	cAyo3452	L. Apoyo	DQ230005	1
A. astorquii	cAyo3453	L. Apoyo	DQ230032	5
A. astorquii	cAyo3454	L. Apoyo	DQ230041	15
A. astorquii	cAyo3455	L. Apoyo	DQ230067	4

A. astorquii	cAyo3473	L. Apoyo	DQ230014	1
A. astorquii	cAyo3475	L. Apoyo	DQ230063	23
A. astorquii	cAyo3476	L. Apoyo	DQ230042	15
A. astorquii	cAyo3478	L. Apoyo	DQ230016	1
A. astorquii	cAyo3479	L. Apoyo	DQ230043	20
A. astorquii	cAyo3481	L. Apoyo	DQ230070	37
A. astorquii	cAyo3564	L. Apoyo	DQ230035	6
A. astorquii	cAyo3963	L. Apoyo	DQ230038	9
A. astorquii	cAyo3966	L. Apoyo	DQ230039	39
A. astorquii	cAyo3967	L. Apoyo	DQ230037	8
A. astorquii	cAyo3972	L. Apoyo	DQ230024	1
A. astorquii	cAyo5049	L. Apoyo	HM183620	37
A. astorquii	cAyo5050	L. Apoyo	HM183621	37
A. astorquii	cAyo5051	L. Apoyo	HM183561	7
A. astorquii	cAyo5053	L. Apoyo	HM183583	23
A. astorquii	cAyo5420	L. Apoyo	HM183584	23
A. astorquii	cAyo5425	L. Apoyo	HM183622	37
A. astorquii	cAyo5426	L. Apoyo	HM183527	1
A. astorquii	cAyo5427	L. Apoyo	HM183557	3
A. astorquii	cAyo5428	L. Apoyo	HM183585	23
A. astorquii	cAyo5429	L. Apoyo	HM183528	1
A. astorquii	cAyo5430	L. Apoyo	HM183529	1
A. astorquii	cAyo5431	L. Apoyo	HM183530	1
A. astorquii	cAyo5433	L. Apoyo	HM183531	1
A. astorquii	cAyo5434	L. Apoyo	HM183609	30
A. astorquii	cAyo5435	L. Apoyo	HM183610	30
A. astorquii	cAyo5436	L. Apoyo	HM183532	1
A. astorquii	cAyo5437	L. Apoyo	HM183533	1
A. astorquii	cAyo5438	L. Apoyo	HM183534	1
A. astorquii	cAyo5439	L. Apoyo	HM183586	23
A. astorquii	cAyo5440	L. Apoyo	HM183535	1
A. astorquii	cAyo5441	L. Apoyo	HM183587	23
A. astorquii	cAyo5442	L. Apoyo	HM183588	23
A. astorquii	cAyo5443	L. Apoyo	HM183589	23
A. astorquii	cAyo5444	L. Apoyo	HM183611	30
A. astorquii	cAyo5445	L. Apoyo	HM183536	1
A. astorquii	cAyo5446	L. Apoyo	HM183537	1
A. astorquii	cAyo5447	L. Apoyo	HM183612	30
A. astorquii	cAyo5676	L. Apoyo	HM183606	28
A. astorquii	cAyo5678	L. Apoyo	HM183602	24
A. astorquii	cAyo5684	L. Apoyo	HM183590	23
A. astorquii	cAyo5685	L. Apoyo	HM183591	23
A. astorquii	cAyo5686	L. Apoyo	HM183623	37
A. astorquii	cAyo5687	L. Apoyo	HM183569	15
A. astorquii	cAyo5690	L. Apoyo	HM183592	23
A. astorquii	cAyo5691	L. Apoyo	HM183607	29
A. astorquii	cAyo5692	L. Apoyo	HM183538	1
A. astorquii	cAyo5693	L. Apoyo	HM183593	23
A. astorquii	cAyo5694	L. Apoyo	HM183615	31
A. astorquii	cAyo5695	L. Apoyo	HM183555	2
A. astorquii	cAyo5696	L. Apoyo	HM183594	23
A. astorquii	cAyo5698	L. Apoyo	HM183613	30
A. astorquii	cAyo5699	L. Apoyo	HM183539	1

A. astorquii	cAyo5701	L. Apoyo	HM183595	23
A. astorquii	cAyo5702	L. Apoyo	HM183558	3
A. astorquii	cAyo5703	L. Apoyo	HM183626	39
A. astorquii	cAyo5704	L. Apoyo	HM183540	1
A. astorquii	cAyo5705	L. Apoyo	HM183556	2
A. astorquii	cAyo5706	L. Apoyo	HM183541	1
A. astorquii	cAyo5707	L. Apoyo	HM183559	4
A. astorquii	cAyo5713	L. Apoyo	HM183614	30
A. astorquii	cAyo5715	L. Apoyo	HM183542	1
A. astorquii	cAyo5716	L. Apoyo	HM183596	23
A. astorquii	cAyo5717	L. Apoyo	HM183597	23
A. astorquii	cAyo5718	L. Apoyo	HM183543	1
A. astorquii	cAyo5719	L. Apoyo	HM183598	23
A. astorquii	cAyo5721	L. Apoyo	HM183544	1
A. astorquii	cAyo5722	L. Apoyo	HM183624	37
A. astorquii	cAyo5723	L. Apoyo	HM183599	23
A. chancho	cAyo3120	L. Apoyo	DQ230044	21
A. chancho	cAyo3133	L. Apoyo	DQ230045	21
A. chancho	cAyo3144	L. Apoyo	DQ230046	21
A. chancho	cAyo3145	L. Apoyo	DQ230076	17
A. chancho	cAyo3149	L. Apoyo	DQ230069	36
A. chancho	cAyo3152	L. Apoyo	DQ230077	17
A. chancho	cAyo3158	L. Apoyo	DQ229991	1
A. chancho	cAyo3165	L. Apoyo	DQ230075	27
A. chancho	cAyo3470	L. Apoyo	DQ230078	17
A. chancho	cAyo3480	L. Apoyo	DQ230017	1
A. chancho	cAyo3960	L. Apoyo	DQ230066	22
A. chancho	cAyo3961	L. Apoyo	DQ230029	19
A. chancho	cAyo3962	L. Apoyo	DQ230079	17
A. chancho	cAyo3970	L. Apoyo	DQ230022	1
A. chancho	cAyo3971	L. Apoyo	DQ230023	1
A. chancho	cAyo3974	L. Apoyo	DQ230025	1
A. chancho	cAyo3975	L. Apoyo	DQ230026	1
A. chancho	cAyo3977	L. Apoyo	DQ230028	1
A. chancho	cAyo3979	L. Apoyo	DQ230034	5
A. chancho	cAyo3980	L. Apoyo	DQ230080	17
A. chancho	cAyo3981	L. Apoyo	DQ230071	37
A. chancho	cAyo5052	L. Apoyo	HM183600	23
A. chancho	cAyo5412	L. Apoyo	HM183617	34
A. chancho	cAyo5432	L. Apoyo	HM183604	26
A. chancho	cAyo5663	L. Apoyo	HM183571	17
A. chancho	cAyo5664	L. Apoyo	HM183570	16
A. chancho	cAyo5665	L. Apoyo	HM183545	1
A. chancho	cAyo5666	L. Apoyo	HM183572	17
A. chancho	cAyo5667	L. Apoyo	HM183573	17
A. chancho	cAyo5671	L. Apoyo	HM183546	1
A. chancho	cAyo5672	L. Apoyo	HM183547	1
A. chancho	cAyo5673	L. Apoyo	HM183548	1
A. chancho	cAyo5674	L. Apoyo	HM183574	17
A. chancho	cAyo5675	L. Apoyo	HM183549	1
A. chancho	cAyo5677	L. Apoyo	HM183576	18
A. chancho	cAyo5679	L. Apoyo	HM183575	17
A. chancho	cAyo5688	L. Apoyo	HM183560	5

A. chancho	cAyo5700	L. Apoyo	HM183601	23
A. chancho	cAyo5720	L. Apoyo	HM183550	1
A. chancho	cAyo5724	L. Apoyo	HM183551	1
A. chancho	cAyo5726	L. Apoyo	HM183605	26
*A. citrinellus**	cAyo3123	L. Apoyo	DQ229968	1
*A. citrinellus**	cAyo3127	L. Apoyo	DQ229971	1
*A. citrinellus**	cAyo3136	L. Apoyo	DQ229978	1
*A. citrinellus**	cAyo3143	L. Apoyo	DQ229983	1
*A. citrinellus**	cAyo3148	L. Apoyo	DQ230059	23
*A. citrinellus**	cAyo3157	L. Apoyo	DQ229990	1
*A. citrinellus**	cAyo3161	L. Apoyo	DQ230030	5
*A. citrinellus**	cAyo3163	L. Apoyo	DQ230047	21
*A. citrinellus**	cAyo3164	L. Apoyo	DQ230036	8
*A. citrinellus**	cAyo3166	L. Apoyo	DQ229995	1
*A. citrinellus**	cAyo3441	L. Apoyo	DQ229997	1
*A. citrinellus**	cAyo3443	L. Apoyo	DQ229999	1
*A. citrinellus**	cAyo3450	L. Apoyo	DQ230003	1
*A. citrinellus**	cAyo3451	L. Apoyo	DQ230004	1
*A. citrinellus**	cAyo3456	L. Apoyo	DQ230006	1
*A. citrinellus**	cAyo3457	L. Apoyo	DQ230033	5
*A. citrinellus**	cAyo3464	L. Apoyo	DQ230049	30
*A. citrinellus**	cAyo3466	L. Apoyo	DQ230068	4
*A. citrinellus**	cAyo3467	L. Apoyo	DQ230012	1
*A. citrinellus**	cAyo3469	L. Apoyo	DQ230072	38
*A. citrinellus**	cAyo3471	L. Apoyo	DQ230062	23
*A. citrinellus**	cAyo3472	L. Apoyo	DQ230050	30
*A. citrinellus**	cAyo3477	L. Apoyo	DQ230081	32
*A. citrinellus**	cAyo3565	L. Apoyo	DQ230065	13
*A. citrinellus**	cAyo3566	L. Apoyo	DQ230073	38
*A. citrinellus**	cAyo3973	L. Apoyo	DQ230064	23
*A. citrinellus**	cAyo3978	L. Apoyo	DQ230074	38
*A. citrinellus**	cAyo5708	L. Apoyo	HM183552	1
*A. citrinellus**	cAyo5714	L. Apoyo	HM183616	33
*A. citrinellus**	cAyo5725	L. Apoyo	HM183553	1
*A. citrinellus**	cAyo5727	L. Apoyo	HM183580	21
*A. citrinellus**	cAyo5728	L. Apoyo	HM183554	1
A. flaveolus	cAyo5668	L. Apoyo	HM183564	11
A. flaveolus	cAyo5669	L. Apoyo	HM183562	8
A. flaveolus	cAyo5670	L. Apoyo		-
A. flaveolus	cAyo5680	L. Apoyo	HM183625	37
A. flaveolus	cAyo5681	L. Apoyo	HM183563	10
A. flaveolus	cAyo5682	L. Apoyo	HM183603	25
A. flaveolus	cAyo5683	L. Apoyo	HM183565	12
A. citrinellus	cAye4048	L. Apoyeque	HM184500	C
A. citrinellus	cAye4049	L. Apoyeque		-
A. citrinellus	cAye4050	L. Apoyeque		-
A. citrinellus	cAye4051	L. Apoyeque		-
A. citrinellus	cAye4052	L. Apoyeque		-
A. citrinellus	cAye4053	L. Apoyeque		-
A. citrinellus	cAye4054	L. Apoyeque	HM184501	C
A. citrinellus	cAye4055	L. Apoyeque	HM184502	C
A. citrinellus	cAye4056	L. Apoyeque		-

A. citrinellus	cAye4057	L. Apoyeque	HM184503	C
A. citrinellus	cAye5003	L. Apoyeque	HM184504	C
A. citrinellus	cAye5004	L. Apoyeque	HM184505	C
A. citrinellus	cAye5005	L. Apoyeque	HM183637	42
A. citrinellus	cAye5006	L. Apoyeque		-
A. citrinellus	cAye5007	L. Apoyeque		-
A. citrinellus	cAye5008	L. Apoyeque	HM184506	C
A. citrinellus	cAye5009	L. Apoyeque	HM184507	C
A. citrinellus	cAye5010	L. Apoyeque	HM184508	C
A. citrinellus	cAye5011	L. Apoyeque	HM184509	C
A. citrinellus	cAye5013	L. Apoyeque	HM184511	C
A. citrinellus	cAye5113	L. Apoyeque	HM184512	C
A. citrinellus	cAye5115	L. Apoyeque	HM184514	C
A. citrinellus	cAye5116	L. Apoyeque	HM184515	C
A. citrinellus	cAye5117	L. Apoyeque	HM183638	42
A. citrinellus	cAye5118	L. Apoyeque	HM184516	C
A. citrinellus	cAye5120	L. Apoyeque	HM184518	C
A. citrinellus	cAye5121	L. Apoyeque	HM184519	C
A. citrinellus	cAye5122	L. Apoyeque		-
A. citrinellus	cAye5123	L. Apoyeque	HM184520	C
A. citrinellus	cAye5124	L. Apoyeque	HM184521	C
A. citrinellus	cAye5125	L. Apoyeque	HM184522	C
A. citrinellus	cAye5126	L. Apoyeque	HM184523	C
A. citrinellus	cAye5127	L. Apoyeque		-
A. citrinellus	cAye5128	L. Apoyeque	HM184524	C
A. citrinellus	cAye5129	L. Apoyeque	HM184525	C
A. citrinellus	cAye5130	L. Apoyeque	HM184526	C
A. citrinellus	cAye5131	L. Apoyeque	HM184527	C
A. citrinellus	cAye5132	L. Apoyeque	HM184528	C
A. citrinellus	cAye5133	L. Apoyeque	HM184529	C
A. citrinellus	cAye5135	L. Apoyeque	HM184531	C
A. citrinellus	cAye5137	L. Apoyeque	HM184533	C
A. citrinellus	cAye5138	L. Apoyeque	HM183639	42
A. citrinellus	cAye5139	L. Apoyeque	HM184534	C
A. citrinellus	cAye5140	L. Apoyeque	HM184535	C
A. citrinellus	cAye5142	L. Apoyeque	HM184537	C
A. citrinellus	cAye5144	L. Apoyeque	HM184539	C
A. citrinellus	cAye5145	L. Apoyeque	HM184540	C
A. citrinellus	cAye5146	L. Apoyeque	HM184541	C
A. citrinellus	cAye5147	L. Apoyeque	HM184542	C
A. citrinellus	cAye5148	L. Apoyeque	HM184543	C
A. citrinellus	cAye5150	L. Apoyeque	HM184545	C
A. citrinellus	cAye5151	L. Apoyeque	HM184546	C
A. citrinellus	cAye5152	L. Apoyeque	HM184547	C
A. citrinellus	cAye5154	L. Apoyeque	HM184549	C
A. citrinellus	cAye5155	L. Apoyeque	HM184550	C
A. citrinellus	cAye5157	L. Apoyeque	HM184552	C
A. citrinellus	cAye5158	L. Apoyeque	HM183641	44
A. citrinellus	cAye5159	L. Apoyeque	HM183640	43
A. citrinellus	cAye5160	L. Apoyeque	HM184553	C
A. citrinellus	cAye5161	L. Apoyeque	HM184554	C
A. citrinellus	cAye5162	L. Apoyeque	HM184555	C
A. citrinellus	cAye5163	L. Apoyeque	HM184556	C

A. citrinellus	cAye5164	L. Apoyeque	HM184557	C
A. citrinellus	cAye5165	L. Apoyeque	HM184558	C
A. citrinellus	cAye5166	L. Apoyeque	HM184559	C
A. citrinellus	cAye5167	L. Apoyeque	HM184560	C
A. citrinellus	cAye5168	L. Apoyeque	HM184561	C
A. citrinellus	cAye5169	L. Apoyeque	HM184562	C
A. citrinellus	cAye5170	L. Apoyeque	HM184563	C
A. citrinellus	cAye5171	L. Apoyeque		-
A. citrinellus	cAye5172	L. Apoyeque	HM184564	C
A. citrinellus	cAye5173	L. Apoyeque	HM184565	C
A. citrinellus	cAye5174	L. Apoyeque	HM184566	C
A. citrinellus	cAye5175	L. Apoyeque	HM184567	C
A. citrinellus	cAye5176	L. Apoyeque	HM184568	C
A. citrinellus	cAye5177	L. Apoyeque	HM184569	C
A. citrinellus	cAye5252	L. Apoyeque	HM184570	C
A. citrinellus	cAye5253	L. Apoyeque	HM184571	C
A. citrinellus	cAye5254	L. Apoyeque	HM184572	C
A. citrinellus	cAye5255	L. Apoyeque	HM184573	C
A. citrinellus	cAye5257	L. Apoyeque	HM184575	C
A. citrinellus	cAye5258	L. Apoyeque	HM184576	C
A. citrinellus	cAye5259	L. Apoyeque	HM184577	C
A. citrinellus	cAye5260	L. Apoyeque	HM184578	C
A. citrinellus	cAye5262	L. Apoyeque	HM184580	C
A. citrinellus	cAye5263	L. Apoyeque	HM184581	C
A. citrinellus	cAye5264	L. Apoyeque	HM184582	C
A. citrinellus lipped	cAye5012	L. Apoyeque	HM184510	C
A. citrinellus lipped	cAye5114	L. Apoyeque	HM184513	C
A. citrinellus lipped	cAye5119	L. Apoyeque	HM184517	C
A. citrinellus lipped	cAye5134	L. Apoyeque	HM184530	C
A. citrinellus lipped	cAye5136	L. Apoyeque	HM184532	C
A. citrinellus lipped	cAye5141	L. Apoyeque	HM184536	C
A. citrinellus lipped	cAye5143	L. Apoyeque	HM184538	C
A. citrinellus lipped	cAye5149	L. Apoyeque	HM184544	C
A. citrinellus lipped	cAye5153	L. Apoyeque	HM184548	C
A. citrinellus lipped	cAye5156	L. Apoyeque	HM184551	C
A. citrinellus lipped	cAye5256	L. Apoyeque	HM184574	C
A. citrinellus lipped	cAye5261	L. Apoyeque	HM184579	C
A. citrinellus	cAsL5336	L. Asososca Leon	HM183642	45
A. citrinellus	cAsL5337	L. Asososca Leon	HM183667	49
A. citrinellus	cAsL5338	L. Asososca Leon	HM183657	47
A. citrinellus	cAsL5339	L. Asososca Leon	HM183643	45
A. citrinellus	cAsL5340	L. Asososca Leon	HM183644	45
A. citrinellus	cAsL5341	L. Asososca Leon	HM183662	48
A. citrinellus	cAsL5342	L. Asososca Leon	HM183670	51
A. citrinellus	cAsL5343	L. Asososca Leon	HM183681	53
A. citrinellus	cAsL5344	L. Asososca Leon	HM183645	45
A. citrinellus	cAsL5345	L. Asososca Leon	HM183671	51
A. citrinellus	cAsL5347	L. Asososca Leon	HM183677	52
A. citrinellus	cAsL5348	L. Asososca Leon	HM183646	45
A. citrinellus	cAsL5350	L. Asososca Leon	HM183678	52
A. citrinellus	cAsL5353	L. Asososca Leon	HM183656	46
A. citrinellus	cAsL5354	L. Asososca Leon	HM183647	45

A. citrinellus	cAsL5357	L. Asososca Leon	HM183648	45
A. citrinellus	cAsL5358	L. Asososca Leon	HM183663	48
A. citrinellus	cAsL5359	L. Asososca Leon	HM183669	50
A. citrinellus	cAsL5360	L. Asososca Leon	HM183672	51
A. citrinellus	cAsL5361	L. Asososca Leon	HM183668	49
A. citrinellus	cAsL5362	L. Asososca Leon	HM183664	48
A. citrinellus	cAsL5363	L. Asososca Leon	HM183679	52
A. citrinellus	cAsL5365	L. Asososca Leon	HM183658	47
A. citrinellus	cAsL5366	L. Asososca Leon	HM183680	52
A. citrinellus	cAsL5367	L. Asososca Leon	HM183673	51
A. citrinellus	cAsL5368	L. Asososca Leon	HM183649	45
A. citrinellus	cAsL5369	L. Asososca Leon	HM183665	48
A. citrinellus	cAsL5370	L. Asososca Leon	HM183650	45
A. citrinellus	cAsL5371	L. Asososca Leon	HM183659	47
A. citrinellus	cAsL5372	L. Asososca Leon	HM183651	45
A. citrinellus	cAsL5373	L. Asososca Leon	HM183652	45
A. citrinellus	cAsL5374	L. Asososca Leon	HM183660	47
A. citrinellus	cAsL5375	L. Asososca Leon	HM183653	45
A. citrinellus elongated	cAsL5346	L. Asososca Leon	HM183654	45
A. citrinellus elongated	cAsL5349	L. Asososca Leon	HM183674	51
A. citrinellus elongated	cAsL5351	L. Asososca Leon	HM183655	45
A. citrinellus elongated	cAsL5352	L. Asososca Leon	HM183666	48
A. citrinellus elongated	cAsL5355	L. Asososca Leon	HM183661	47
A. citrinellus elongated	cAsL5356	L. Asososca Leon	HM183675	51
A. citrinellus elongated	cAsL5364	L. Asososca Leon	HM183676	51
A. citrinellus	cAsM5554	L. Asososca Managua	HM183685	55
A. citrinellus	cAsM5555	L. Asososca Managua	HM183686	55
A. citrinellus	cAsM5556	L. Asososca Managua	HM183682	54
A. citrinellus	cAsM5557	L. Asososca Managua	HM184583	C
A. citrinellus	cAsM5558	L. Asososca Managua	HM183687	55
A. citrinellus	cAsM5559	L. Asososca Managua	HM183712	56
A. citrinellus	cAsM5560	L. Asososca Managua	HM183688	55
A. citrinellus	cAsM5561	L. Asososca Managua	HM183716	59
A. citrinellus	cAsM5562	L. Asososca Managua	HM183689	55
A. citrinellus	cAsM5563	L. Asososca Managua	HM183717	59
A. citrinellus	cAsM5564	L. Asososca Managua	HM183690	55
A. citrinellus	cAsM5566	L. Asososca Managua	HM183691	55
A. citrinellus	cAsM5567	L. Asososca Managua	HM183692	55
A. citrinellus	cAsM5568	L. Asososca Managua	HM183693	55
A. citrinellus	cAsM5569	L. Asososca Managua	HM183683	54
A. citrinellus	cAsM5571	L. Asososca Managua	HM184584	C
A. citrinellus	cAsM5572	L. Asososca Managua	HM183694	55
A. citrinellus	cAsM5573	L. Asososca Managua	HM184585	C
A. citrinellus	cAsM5575	L. Asososca Managua	HM183713	57
A. citrinellus	cAsM5576	L. Asososca Managua	HM184586	C
A. citrinellus	cAsM5577	L. Asososca Managua	HM183695	55
A. citrinellus	cAsM5578	L. Asososca Managua	HM183696	55
A. citrinellus	cAsM5579	L. Asososca Managua	HM184587	C
A. citrinellus	cAsM5580	L. Asososca Managua	HM183697	55
A. citrinellus	cAsM5581	L. Asososca Managua	HM183698	55
A. citrinellus	cAsM5582	L. Asososca Managua	HM183699	55
A. citrinellus	cAsM5583	L. Asososca Managua	HM183700	55

A. citrinellus	cAsM5584	L. Asososca Managua	HM183701	55
A. citrinellus	cAsM5585	L. Asososca Managua	HM183702	55
A. citrinellus	cAsM5586	L. Asososca Managua	HM183703	55
A. citrinellus	cAsM5587	L. Asososca Managua	HM183704	55
A. citrinellus	cAsM5588	L. Asososca Managua	HM183705	55
A. citrinellus	cAsM5589	L. Asososca Managua	HM183706	55
A. citrinellus	cAsM5590	L. Asososca Managua	HM183707	55
A. citrinellus	cAsM5591	L. Asososca Managua	HM184588	C
A. citrinellus	cAsM5592	L. Asososca Managua	HM183714	58
A. citrinellus	cAsM5593	L. Asososca Managua	HM183708	55
A. citrinellus	cAsM5594	L. Asososca Managua	HM184589	C
A. citrinellus	cAsM5595	L. Asososca Managua	HM183709	55
A. citrinellus	cAsM5597	L. Asososca Managua	HM184590	C
A. citrinellus elongated	cAsM5553	L. Asososca Managua	HM183710	55
A. citrinellus elongated	cAsM5565	L. Asososca Managua	HM184591	C
A. citrinellus elongated	cAsM5574	L. Asososca Managua	HM183711	55
A. citrinellus gold	cAsM5596	L. Asososca Managua	HM183715	58
A. citrinellus	cMas1365	L. Masaya	AY567208	78
A. citrinellus	cMas1367	L. Masaya	AY567209	70
A. citrinellus	cMas1368	L. Masaya	AY567210	C
A. citrinellus	cMas1369	L. Masaya	AY567211	C
A. citrinellus	cMas1370	L. Masaya	AY567212	70
A. citrinellus	cMas1371	L. Masaya	AY567213	76
A. citrinellus	cMas1372	L. Masaya	AY567214	68
A. citrinellus	cMas1373	L. Masaya	AY567215	63
A. citrinellus	cMas1374	L. Masaya	AY567216	70
A. citrinellus	cMas1375	L. Masaya	AY567217	60
A. citrinellus	cMas1376	L. Masaya	AY567218	C
A. citrinellus	cMas1377	L. Masaya	AY567219	C
A. citrinellus	cMas1378	L. Masaya	AY567220	C
A. citrinellus	cMas1379	L. Masaya	AY567221	C
A. citrinellus	cMas1380	L. Masaya	AY567222	C
A. citrinellus	cMas1381	L. Masaya	AY567223	C
A. citrinellus	cMas1382	L. Masaya	AY567224	70
A. citrinellus	cMas1383	L. Masaya	AY567225	C
A. citrinellus	cMas1384	L. Masaya	AY567226	70
A. citrinellus	cMas1385	L. Masaya	AY567227	70
A. citrinellus	cMas1386	L. Masaya	AY567228	63
A. citrinellus	cMas1387	L. Masaya	AY567229	72
A. citrinellus	cMas1388	L. Masaya	AY567230	68
A. citrinellus	cMas1389	L. Masaya	AY567231	70
A. citrinellus	cMas1390	L. Masaya	AY567232	78
A. citrinellus	cMas1391	L. Masaya	AY567233	78
A. citrinellus	cMas1392	L. Masaya	AY567234	67
A. citrinellus	cMas1393	L. Masaya	AY567235	C
A. citrinellus	cMas1394	L. Masaya	AY567236	70
A. citrinellus	cMas1395	L. Masaya	AY567237	70
A. citrinellus	cMas1396	L. Masaya	AY567238	70
A. citrinellus	cMas1397	L. Masaya	AY567239	78
A. citrinellus	cMas1398	L. Masaya	AY567240	70
A. citrinellus	cMas1399	L. Masaya	AY567241	70
A. citrinellus	cMas1400	L. Masaya	AY567242	63

A. citrinellus	cMas1436	L. Masaya	AY567243	63
A. citrinellus	cMas3169	L. Masaya	EF157347	C
A. citrinellus	cMas3170	L. Masaya	EF157348	C
A. citrinellus	cMas3172	L. Masaya	EF157349	C
A. citrinellus	cMas3173	L. Masaya	EF157350	68
A. citrinellus	cMas3176	L. Masaya	EF157351	C
A. citrinellus	cMas3177	L. Masaya	EF157352	70
A. citrinellus	cMas3178	L. Masaya	EF157353	70
A. citrinellus	cMas3179	L. Masaya	EF157354	60
A. citrinellus	cMas3180	L. Masaya	EF157355	C
A. citrinellus	cMas3187	L. Masaya	EF157356	72
A. citrinellus	cMas3188	L. Masaya	EF157357	C
A. citrinellus	cMas3190	L. Masaya	EF157358	C
A. citrinellus	cMas3211	L. Masaya	EF157359	63
A. citrinellus	cMas3214	L. Masaya	EF157360	C
A. citrinellus	cMas3215	L. Masaya	EF157361	70
A. citrinellus	cMas3216	L. Masaya	EF157362	75
A. citrinellus	cMas3217	L. Masaya	EF157363	63
A. citrinellus	cMas3218	L. Masaya	EF157364	C
A. citrinellus	cMas3219	L. Masaya	EF157365	64
A. citrinellus	cMas3220	L. Masaya	EF157366	78
A. citrinellus	cMas3221	L. Masaya	EF157367	C
A. citrinellus	cMas3222	L. Masaya	EF157368	70
A. citrinellus	cMas3223	L. Masaya	EF157369	C
A. citrinellus	cMas3224	L. Masaya	EF157370	74
A. citrinellus	cMas3226	L. Masaya	EF157372	73
A. citrinellus	cMas3231	L. Masaya	EF157373	68
A. citrinellus	cMas3498	L. Masaya	EF157427	78
A. citrinellus	cMas3499	L. Masaya	EF157428	78
A. citrinellus	cMas3500	L. Masaya	EF157429	67
A. citrinellus	cMas3501	L. Masaya	EF157430	61
A. citrinellus	cMas3502	L. Masaya	EF157431	78
A. citrinellus	cMas3505	L. Masaya	EF157432	73
A. citrinellus	cMas3506	L. Masaya	EF157433	C
A. citrinellus	cMas3507	L. Masaya	EF157434	C
A. citrinellus	cMas3510	L. Masaya	EF157435	70
A. citrinellus	cMas3512	L. Masaya	EF157436	70
A. citrinellus	cMas3520	L. Masaya	EF157437	C
A. citrinellus	cMas3521	L. Masaya	EF157438	C
A. citrinellus	cMas3524	L. Masaya	EF157439	70
A. citrinellus	cMas3525	L. Masaya	EF157440	C
A. citrinellus	cMas3526	L. Masaya	EF157441	61
A. citrinellus	cMas3527	L. Masaya	EF157442	63
A. citrinellus	cMas3528	L. Masaya	EF157443	C
A. citrinellus	cMas3529	L. Masaya	EF157444	70
A. citrinellus	cMas3530	L. Masaya	EF157445	70
A. citrinellus	cMas3531	L. Masaya	EF157446	C
A. citrinellus	cMas3535	L. Masaya	EF157448	C
A. citrinellus	cMas3536	L. Masaya	EF157449	C
A. citrinellus	cMas3537	L. Masaya	EF157450	60
A. citrinellus	cMas3539	L. Masaya	EF157451	63
A. citrinellus	cMas3540	L. Masaya	EF157452	C
A. citrinellus	cMas3541	L. Masaya	EF157453	C

A. citrinellus	cMas3542	L. Masaya	EF157454	C
A. citrinellus	cMas3544	L. Masaya	EF157455	70
A. citrinellus	cMas3545	L. Masaya	EF157456	61
A. citrinellus	cMas3546	L. Masaya	EF157457	61
A. citrinellus	cMas3547	L. Masaya	EF157458	63
A. citrinellus	cMas3548	L. Masaya	EF157459	C
A. citrinellus	cMas3549	L. Masaya	EF157460	61
A. citrinellus	cMas3550	L. Masaya	EF157461	C
A. citrinellus	cMas3551	L. Masaya	EF157462	70
A. citrinellus	cMas3552	L. Masaya	EF157463	70
A. citrinellus	cMas3553	L. Masaya	EF157464	C
A. citrinellus	cMas3554	L. Masaya	EF157465	C
A. citrinellus	cMas3555	L. Masaya	EF157466	62
A. citrinellus	cMas3556	L. Masaya	EF157467	70
A. citrinellus	cMas3557	L. Masaya	EF157468	70
A. citrinellus	cMas3558	L. Masaya	EF157469	C
A. citrinellus	cMas3559	L. Masaya	EF157470	60
A. citrinellus	cMas3560	L. Masaya	EF157471	63
A. citrinellus	cMas3561	L. Masaya	EF157472	60
A. citrinellus	cMas3562	L. Masaya	EF157473	C
A. citrinellus	cMas3563	L. Masaya	EF157474	C
A. citrinellus	cMas3567	L. Masaya	EF157475	70
A. citrinellus	cMas3568	L. Masaya	EF157476	C
A. citrinellus	cMas3851	L. Masaya	EF157539	70
A. citrinellus	cMas3854	L. Masaya	EF157540	C
A. citrinellus	cMas3855	L. Masaya	EF157541	C
A. citrinellus	cMas3858	L. Masaya	EF157542	70
A. citrinellus	cMas3862	L. Masaya	EF157543	68
A. citrinellus	cMas3865	L. Masaya	EF157544	61
A. citrinellus	cMas3866	L. Masaya	EF157545	C
A. citrinellus	cMas3867	L. Masaya	EF157546	70
A. citrinellus	cMas3868	L. Masaya	EF157547	71
A. citrinellus	cMas3871	L. Masaya	EF157548	78
A. citrinellus	cMas3872	L. Masaya	EF157549	70
A. citrinellus	cMas3874	L. Masaya	EF157550	70
A. citrinellus	cMas3875	L. Masaya	EF157551	63
A. citrinellus	cMas3876	L. Masaya	EF157552	70
A. citrinellus	cMas3877	L. Masaya	EF157553	70
A. citrinellus	cMas3878	L. Masaya	EF157554	72
A. citrinellus	cMas3880	L. Masaya	EF157555	70
A. citrinellus	cMas3892	L. Masaya	EF157556	C
A. citrinellus	cMas3893	L. Masaya	EF157557	70
A. citrinellus	cMas3894	L. Masaya	EF157558	C
A. citrinellus	cMas3896	L. Masaya	EF157559	77
A. citrinellus	cMas3897	L. Masaya	EF157560	60
A. citrinellus	cMas3898	L. Masaya	EF157561	70
A. citrinellus	cMas3899	L. Masaya	EF157562	70
A. citrinellus	cMas3900	L. Masaya	EF157563	C
A. citrinellus	cMas3901	L. Masaya	EF157564	63
A. citrinellus	cMas3902	L. Masaya	EF157565	60
A. citrinellus	cMas3903	L. Masaya	EF157566	62
A. citrinellus	cMas3904	L. Masaya	EF157567	78
A. citrinellus	cMas3905	L. Masaya	EF157568	63

A. citrinellus	cMas3906	L. Masaya	EF157569	C
A. citrinellus	cMas3907	L. Masaya	EF157570	C
A. citrinellus	cMas3908	L. Masaya	EF157571	60
A. citrinellus	cMas3909	L. Masaya	EF157572	60
A. citrinellus gold	cMas3496	L. Masaya	EF157426	63
A. citrinellus gold	cMas3534	L. Masaya	EF157447	73
A. citrinellus lipped	cMas3225	L. Masaya	EF157371	70
A. citrinellus lipped	cMas3497	L. Masaya	HM183842	78
A. amarillo	cXil3087	L. Xiloa	EF157327	106
A. amarillo	cXil3095	L. Xiloa	EF157329	119
A. amarillo	cXil3234	L. Xiloa	EF157374	C
A. amarillo	cXil3236	L. Xiloa	EF157375	C
A. amarillo	cXil3237	L. Xiloa	EF157376	113
A. amarillo	cXil3238	L. Xiloa	EF157377	C
A. amarillo	cXil3240	L. Xiloa	EF157378	122
A. amarillo	cXil3241	L. Xiloa	EF157379	60
A. amarillo	cXil3243	L. Xiloa	EF157381	C
A. amarillo	cXil3244	L. Xiloa	EF157382	107
A. amarillo	cXil3245	L. Xiloa	EF157383	C
A. amarillo	cXil3247	L. Xiloa	EF157385	C
A. amarillo	cXil3248	L. Xiloa	EF157386	115
A. amarillo	cXil3249	L. Xiloa	EF157387	100
A. amarillo	cXil3250	L. Xiloa	EF157388	116
A. amarillo	cXil3251	L. Xiloa	EF157389	C
A. amarillo	cXil3252	L. Xiloa	EF157390	91
A. amarillo	cXil3254	L. Xiloa	EF157391	C
A. amarillo	cXil3255	L. Xiloa	EF157392	C
A. amarillo	cXil3256	L. Xiloa	EF157393	82
A. amarillo	cXil3257	L. Xiloa	EF157394	C
A. amarillo	cXil3594	L. Xiloa	EF157490	113
A. amarillo	cXil3595	L. Xiloa	EF157491	C
A. amarillo	cXil3596	L. Xiloa	EF157492	C
A. amarillo	cXil3597	L. Xiloa	EF157493	81
A. amarillo	cXil3813	L. Xiloa	EF157518	113
A. amarillo	cXil3814	L. Xiloa	EF157519	C
A. amarillo	cXil3828	L. Xiloa	EF157528	C
A. amarillo	cXil3829	L. Xiloa	EF157529	99
A. amarillo	cXil3830	L. Xiloa	EF157530	103
A. amarillo	cXil3832	L. Xiloa	EF157532	102
A. amarillo	cXil3835	L. Xiloa	EF157535	110
A. amarillo	cXil3836	L. Xiloa	EF157536	C
A. amarillo	cXil5014	L. Xiloa	HM183901	91
A. amarillo	cXil5015	L. Xiloa	HM184592	C
A. amarillo	cXil5021	L. Xiloa	HM184593	C
A. amarillo	cXil5031	L. Xiloa	HM184594	C
A. amarillo	cXil5033	L. Xiloa	HM183843	78
A. amarillo	cXil5037	L. Xiloa	HM183718	60
A. amarillo	cXil5039	L. Xiloa	HM184031	122
A. amarillo	cXil5040	L. Xiloa	HM184595	C
A. amarillo	cXil5042	L. Xiloa	HM184596	C
A. amarillo	cXil5043	L. Xiloa	HM183934	101
A. amarillo	cXil5059	L. Xiloa	HM183984	113

A. amarillo	cXil5060	L. Xiloa	HM183943	104
A. amarillo	cXil5061	L. Xiloa	HM184597	C
A. amarillo	cXil5062	L. Xiloa	HM184032	122
A. amarillo	cXil5063	L. Xiloa	HM184598	C
A. amarillo	cXil5064	L. Xiloa	HM183821	69
A. amarillo	cXil5065	L. Xiloa	HM184599	C
A. amarillo	cXil5066	L. Xiloa	HM184600	C
A. amarillo	cXil5067	L. Xiloa	HM183952	107
A. amarillo	cXil5068	L. Xiloa	HM184601	C
A. amarillo	cXil5069	L. Xiloa	HM184000	115
A. amarillo	cXil5070	L. Xiloa	HM183985	113
A. amarillo	cXil5071	L. Xiloa	HM183719	60
A. amarillo	cXil5072	L. Xiloa	HM184602	C
A. amarillo	cXil5073	L. Xiloa	HM183912	94
A. amarillo	cXil5074	L. Xiloa	HM183924	97
A. amarillo	cXil5075	L. Xiloa	HM184010	116
A. amarillo	cXil5078	L. Xiloa	HM183875	81
A. amarillo	cXil5079	L. Xiloa	HM184603	C
A. amarillo	cXil5080	L. Xiloa	HM183939	103
A. amarillo	cXil5081	L. Xiloa	HM183986	113
A. amarillo	cXil5082	L. Xiloa	HM184033	122
A. amarillo	cXil5083	L. Xiloa	HM184001	115
A. amarillo	cXil5084	L. Xiloa	HM184604	C
A. amarillo	cXil5085	L. Xiloa	HM183720	60
A. amarillo	cXil5086	L. Xiloa	HM184011	116
A. amarillo	cXil5087	L. Xiloa	HM184605	C
A. amarillo	cXil5088	L. Xiloa	HM183876	81
A. amarillo	cXil5089	L. Xiloa	HM184606	C
A. amarillo	cXil5090	L. Xiloa	HM183721	60
A. amarillo	cXil5091	L. Xiloa	HM184492	510
A. amarillo	cXil5092	L. Xiloa	HM183778	61
A. amarillo	cXil5093	L. Xiloa	HM184607	C
A. amarillo	cXil5094	L. Xiloa	HM183800	63
A. amarillo	cXil5095	L. Xiloa	HM184608	C
A. amarillo	cXil5096	L. Xiloa	HM183779	61
A. amarillo	cXil5097	L. Xiloa	HM183987	113
A. amarillo	cXil5098	L. Xiloa	HM184609	C
A. amarillo	cXil5099	L. Xiloa	HM184012	116
A. amarillo	cXil5100	L. Xiloa	HM183937	102
A. amarillo	cXil5101	L. Xiloa	HM184610	C
A. amarillo	cXil5103	L. Xiloa	HM184611	C
A. amarillo	cXil5112	L. Xiloa	HM184612	C
A. amarillo	cXil5179	L. Xiloa	HM184613	C
A. amarillo	cXil5180	L. Xiloa	HM183780	61
A. amarillo	cXil5181	L. Xiloa	HM184614	C
A. amarillo	cXil5183	L. Xiloa	HM183902	91
A. amarillo	cXil5184	L. Xiloa	HM183877	81
A. amarillo	cXil5185	L. Xiloa	HM184615	C
A. amarillo	cXil5186	L. Xiloa	HM184616	C
A. amarillo	cXil5188	L. Xiloa	HM183938	102
A. amarillo	cXil5190	L. Xiloa	HM183801	63
A. amarillo	cXil5191	L. Xiloa	HM184617	C
A. amarillo	cXil5192	L. Xiloa	HM184034	122

A. amarillo	cXil5193	L. Xiloa	HM184618	C
A. amarillo	cXil5195	L. Xiloa	HM183900	90
A. amarillo	cXil5196	L. Xiloa	HM184002	115
A. amarillo	cXil5197	L. Xiloa	HM184619	C
A. amarillo	cXil5198	L. Xiloa	HM184028	120
A. amarillo	cXil5199	L. Xiloa	HM184620	C
A. amarillo	cXil5201	L. Xiloa	HM184621	C
A. amarillo	cXil5202	L. Xiloa	HM184622	C
A. amarillo	cXil5203	L. Xiloa	HM183974	110
A. amarillo	cXil5204	L. Xiloa	HM183913	94
A. amarillo	cXil5206	L. Xiloa	HM184003	115
A. amarillo	cXil5207	L. Xiloa	HM184623	C
A. amarillo	cXil5208	L. Xiloa	HM184624	C
A. amarillo	cXil5209	L. Xiloa	HM184064	123
A. amarillo	cXil5211	L. Xiloa	HM184625	C
A. amarillo	cXil5212	L. Xiloa	HM183722	60
A. amarillo	cXil5213	L. Xiloa	HM183914	94
A. amarillo	cXil5214	L. Xiloa	HM183926	99
A. amarillo	cXil5218	L. Xiloa	HM183903	91
A. amarillo	cXil5219	L. Xiloa	HM183988	113
A. amarillo	cXil5220	L. Xiloa	HM183989	113
A. amarillo	cXil5221	L. Xiloa	HM184004	115
A. amarillo	cXil5222	L. Xiloa	HM184626	C
A. amarillo	cXil5223	L. Xiloa	HM184627	C
A. amarillo	cXil5224	L. Xiloa	HM184013	116
A. amarillo	cXil5225	L. Xiloa	HM184628	C
A. amarillo	cXil5227	L. Xiloa	HM183990	113
A. amarillo	cXil5228	L. Xiloa	HM183945	106
A. amarillo	cXil5229	L. Xiloa	HM183781	61
A. amarillo	cXil5230	L. Xiloa	HM184629	C
A. amarillo	cXil5231	L. Xiloa	HM184630	C
A. amarillo	cXil5232	L. Xiloa	HM184631	C
A. amarillo	cXil5233	L. Xiloa	HM184632	C
A. amarillo	cXil5234	L. Xiloa	HM184633	C
A. amarillo	cXil5235	L. Xiloa	HM183935	101
A. amarillo	cXil5236	L. Xiloa	HM183975	110
A. amarillo	cXil5237	L. Xiloa	HM183944	105
A. amarillo	cXil5238	L. Xiloa	HM184634	C
A. amarillo	cXil5239	L. Xiloa	HM183936	101
A. amarillo	cXil5240	L. Xiloa	HM183940	103
A. amarillo	cXil5241	L. Xiloa	HM183991	113
A. amarillo	cXil5242	L. Xiloa	HM183992	113
A. amarillo	cXil5243	L. Xiloa	HM184635	C
A. amarillo	cXil5244	L. Xiloa	HM183976	110
A. amarillo	cXil5245	L. Xiloa	HM184636	C
A. amarillo	cXil5246	L. Xiloa	HM184637	C
A. amarillo	cXil5247	L. Xiloa	HM183782	61
A. amarillo	cXil5248	L. Xiloa	HM184638	C
A. amarillo	cXil5291	L. Xiloa	HM184639	C
A. amarillo	cXil5306	L. Xiloa	HM184035	122
A. amarillo	cXil5307	L. Xiloa	HM184640	C
A. amarillo	cXil5308	L. Xiloa	HM184036	122
A. amarillo	cXil5309	L. Xiloa	HM184014	116

A. amarillo	cXil5310	L. Xiloa	HM184005	115
A. amarillo	cXil5311	L. Xiloa	HM184641	C
A. amarillo	cXil5312	L. Xiloa	HM184642	C
A. amarillo	cXil5313	L. Xiloa	HM184643	C
A. amarillo	cXil5314	L. Xiloa	HM184644	C
A. amarillo	cXil5315	L. Xiloa	HM183904	91
A. amarillo	cXil5316	L. Xiloa	HM184645	C
A. amarillo	cXil5317	L. Xiloa	HM183802	63
A. amarillo	cXil5318	L. Xiloa	HM183783	61
A. amarillo	cXil5319	L. Xiloa	HM183993	113
A. amarillo	cXil5320	L. Xiloa	HM183784	61
A. amarillo	cXil5321	L. Xiloa	HM184037	122
A. amarillo	cXil5322	L. Xiloa	HM183822	69
A. amarillo	cXil5326	L. Xiloa	HM183927	99
A. amarillo	cXil5328	L. Xiloa	HM183928	99
A. amarillo	cXil5329	L. Xiloa	HM184646	C
A. amarillo	cXil5330	L. Xiloa	HM184647	C
A. amarillo	cXil5331	L. Xiloa	HM184648	C
A. amarillo	cXil5332	L. Xiloa	HM183785	61
A. amarillo	cXil5456	L. Xiloa	HM184649	C
A. amarillo	cXil5457	L. Xiloa	HM184650	C
A. amarillo	cXil5458	L. Xiloa	HM184651	C
A. amarillo	cXil5459	L. Xiloa	HM184652	C
A. amarillo	cXil5460	L. Xiloa	HM184076	126
A. amarillo	cXil5461	L. Xiloa	HM184653	C
A. amarillo	cXil5462	L. Xiloa	HM183723	60
A. amarillo	cXil5463	L. Xiloa	HM184654	C
A. amarillo	cXil5464	L. Xiloa	HM183953	107
A. amarillo	cXil5465	L. Xiloa	HM183786	61
A. amarillo	cXil5466	L. Xiloa	HM184655	C
A. amarillo	cXil5467	L. Xiloa	HM183977	110
A. amarillo	cXil5470	L. Xiloa	HM184656	C
A. amarillo	cXil5475	L. Xiloa	HM184015	116
A. amarillo	cXil5476	L. Xiloa	HM184657	C
A. amarillo	cXil5480	L. Xiloa	HM184658	C
A. amarillo	cXil5486	L. Xiloa	HM184659	C
A. amarillo	cXil5487	L. Xiloa	HM184660	C
A. amarillo	cXil5490	L. Xiloa	HM184025	118
A. amarillo	cXil5491	L. Xiloa	HM184661	C
A. amarillo	cXil5492	L. Xiloa	HM183978	110
A. amarillo	cXil5493	L. Xiloa	HM184494	511
A. amarillo	cXil5499	L. Xiloa	HM184006	115
A. amarillo	cXil5522	L. Xiloa	HM184662	C
A. amarillo	cXil5530	L. Xiloa	HM183724	60
A. amarillo	cXil5549	L. Xiloa	HM184663	C
A. amarillo	cXil5550	L. Xiloa	HM184007	115
A. amarillo	cXil5551	L. Xiloa	HM184664	C
A. amarillo	cXil5552	L. Xiloa	HM183946	106
A. amarillo	cXil5745	L. Xiloa	HM183982	111
A. amarillo	cXil5746	L. Xiloa	HM184665	C
A. amarillo	cXil5747	L. Xiloa	HM183878	81
A. amarillo	cXil5748	L. Xiloa	HM184666	C
A. amarillo	cXil5749	L. Xiloa	HM183954	107

A. amarillo	cXil5864	L. Xiloa	HM184667	C
A. amarillo	cXil5865	L. Xiloa	HM183874	80
A. amarillo	cXil5866	L. Xiloa	HM184668	C
A. amarillo	cXil5867	L. Xiloa	HM183915	94
A. amarillo	cXil5868	L. Xiloa	HM184495	511
A. amarillo	cXil5869	L. Xiloa	HM183916	94
A. amarillo	cXil5870	L. Xiloa	HM184669	C
A. amarillo	cXil5871	L. Xiloa	HM183929	99
A. amarillo	cXil5872	L. Xiloa	HM183725	60
A. amarillo	cXil5873	L. Xiloa	HM183930	99
A. amarillo	cXil5874	L. Xiloa	HM183917	94
A. amarillo	cXil5875	L. Xiloa	HM183931	99
A. amarillo	cXil5876	L. Xiloa	HM184670	C
A. amarillo	cXil5877	L. Xiloa	HM184671	C
A. amarillo	cXil5878	L. Xiloa	HM184038	122
A. amarillo	cXil5879	L. Xiloa	HM184672	C
A. amarillo	cXil5880	L. Xiloa	HM184008	115
A. amarillo	cXil5882	L. Xiloa	HM183955	107
A. amarillo	cXil5883	L. Xiloa	HM184017	117
A. amarillo	cXil5884	L. Xiloa	HM183956	107
A. amarillo	cXil5885	L. Xiloa	HM184673	C
A. amarillo	cXil5886	L. Xiloa	HM184674	C
A. amarillo	cXil5887	L. Xiloa	HM183726	60
A. amarillo	cXil5888	L. Xiloa	HM183979	110
A. amarillo	cXil5889	L. Xiloa	HM184675	C
A. amarillo	cXil5890	L. Xiloa	HM184016	116
A. amarillo	cXil5891	L. Xiloa	HM184676	C
A. amarillo	cXil5892	L. Xiloa	HM183941	103
A. amarillo	cXil5893	L. Xiloa	HM184677	C
*A. citrinellus**	cXil1098	L. Xiloa	AY567244	C
*A. citrinellus**	cXil1099	L. Xiloa	AY567245	84
*A. citrinellus**	cXil1100	L. Xiloa	AY567246	115
*A. citrinellus**	cXil1101	L. Xiloa	AY567247	120
*A. citrinellus**	cXil1172	L. Xiloa	AY567248	60
*A. citrinellus**	cXil1173	L. Xiloa	AY567249	116
*A. citrinellus**	cXil1176	L. Xiloa	AY567250	C
*A. citrinellus**	cXil1177	L. Xiloa	AY567251	C
*A. citrinellus**	cXil1576	L. Xiloa	HM183887	88
*A. citrinellus**	cXil1618	L. Xiloa	AY567252	99
*A. citrinellus**	cXil1620	L. Xiloa	AY567253	84
*A. citrinellus**	cXil1621	L. Xiloa	AY567254	69
*A. citrinellus**	cXil1622	L. Xiloa	AY567255	C
*A. citrinellus**	cXil1623	L. Xiloa	AY567256	84
*A. citrinellus**	cXil1624	L. Xiloa	AY567257	84
*A. citrinellus**	cXil1625	L. Xiloa	AY567258	84
*A. citrinellus**	cXil1627	L. Xiloa	AY567259	83
*A. citrinellus**	cXil1628	L. Xiloa	AY567260	99
*A. citrinellus**	cXil1630	L. Xiloa	AY567261	113
*A. citrinellus**	cXil1631	L. Xiloa	AY567262	C
*A. citrinellus**	cXil1633	L. Xiloa	AY567263	C
*A. citrinellus**	cXil1642	L. Xiloa	AY567264	C
*A. citrinellus**	cXil1651	L. Xiloa	AY567265	98
*A. citrinellus**	cXil4058	L. Xiloa	EF157573	121

*A. citrinellus**	cXil5483	L. Xiloa	HM184678	C
*A. citrinellus**	cXil5494	L. Xiloa	HM184679	C
*A. citrinellus**	cXil5495	L. Xiloa	HM183823	69
*A. citrinellus**	cXil5496	L. Xiloa	HM183888	88
*A. citrinellus**	cXil5497	L. Xiloa	HM184039	122
*A. citrinellus**	cXil5498	L. Xiloa	HM184680	C
*A. citrinellus**	cXil5501	L. Xiloa	HM183824	69
*A. citrinellus**	cXil5502	L. Xiloa	HM184040	122
*A. citrinellus**	cXil5503	L. Xiloa	HM184681	C
*A. citrinellus**	cXil5504	L. Xiloa	HM184018	117
*A. citrinellus**	cXil5505	L. Xiloa	HM183803	63
*A. citrinellus**	cXil5506	L. Xiloa	HM184682	C
*A. citrinellus**	cXil5507	L. Xiloa	HM183932	99
*A. citrinellus**	cXil5508	L. Xiloa	HM184683	C
*A. citrinellus**	cXil5509	L. Xiloa	HM183825	69
*A. citrinellus**	cXil5510	L. Xiloa	HM183826	69
*A. citrinellus**	cXil5511	L. Xiloa	HM183787	61
*A. citrinellus**	cXil5512	L. Xiloa	HM184684	C
*A. citrinellus**	cXil5513	L. Xiloa	HM183889	88
*A. citrinellus**	cXil5514	L. Xiloa	HM184019	117
*A. citrinellus**	cXil5515	L. Xiloa	HM183885	86
*A. citrinellus**	cXil5516	L. Xiloa	HM184009	115
*A. citrinellus**	cXil5517	L. Xiloa	HM184041	122
*A. citrinellus**	cXil5518	L. Xiloa	HM184026	118
*A. citrinellus**	cXil5519	L. Xiloa	HM184027	118
*A. citrinellus**	cXil5520	L. Xiloa	HM184685	C
*A. citrinellus**	cXil5521	L. Xiloa	HM184686	C
*A. citrinellus**	cXil5523	L. Xiloa	HM184496	511
*A. citrinellus**	cXil5524	L. Xiloa	HM184042	122
*A. citrinellus**	cXil5525	L. Xiloa	HM183994	113
*A. citrinellus**	cXil5526	L. Xiloa	HM184687	C
*A. citrinellus**	cXil5527	L. Xiloa	HM183942	103
*A. citrinellus**	cXil5528	L. Xiloa	HM183788	61
*A. citrinellus**	cXil5529	L. Xiloa	HM184493	510
*A. citrinellus**	cXil5531	L. Xiloa	HM184043	122
*A. citrinellus**	cXil5532	L. Xiloa	HM183890	88
*A. citrinellus**	cXil5533	L. Xiloa	HM184044	122
*A. citrinellus**	cXil5534	L. Xiloa	HM183957	107
*A. citrinellus**	cXil5535	L. Xiloa	HM184688	C
*A. citrinellus**	cXil5536	L. Xiloa	HM183879	81
*A. citrinellus**	cXil5537	L. Xiloa	HM183881	84
A. sagittae	cXil3242	L. Xiloa	EF157380	121
A. sagittae	cXil3246	L. Xiloa	EF157384	510
A. sagittae	cXil3569	L. Xiloa	EF157477	C
A. sagittae	cXil3575	L. Xiloa	EF157482	107
A. sagittae	cXil3584	L. Xiloa	EF157486	C
A. sagittae	cXil3585	L. Xiloa	EF157487	85
A. sagittae	cXil3587	L. Xiloa	EF157489	87
A. sagittae	cXil3598	L. Xiloa	EF157494	122
A. sagittae	cXil3770	L. Xiloa	EF157499	124
A. sagittae	cXil3775	L. Xiloa	EF157502	122
A. sagittae	cXil3776	L. Xiloa	EF157503	122
A. sagittae	cXil3778	L. Xiloa	EF157504	C

A. sagittae	cXil3779	L. Xiloa	EF157505	89
A. sagittae	cXil3780	L. Xiloa	EF157506	122
A. sagittae	cXil3781	L. Xiloa	EF157507	122
A. sagittae	cXil3783	L. Xiloa	EF157508	122
A. sagittae	cXil3784	L. Xiloa	EF157509	122
A. sagittae	cXil3786	L. Xiloa	EF157510	107
A. sagittae	cXil3805	L. Xiloa	EF157511	122
A. sagittae	cXil3806	L. Xiloa	EF157512	124
A. sagittae	cXil3807	L. Xiloa	EF157513	122
A. sagittae	cXil3810	L. Xiloa	EF157515	122
A. sagittae	cXil3811	L. Xiloa	EF157516	C
A. sagittae	cXil3812	L. Xiloa	EF157517	C
A. sagittae	cXil3815	L. Xiloa	EF157520	C
A. sagittae	cXil3816	L. Xiloa	EF157521	C
A. sagittae	cXil3817	L. Xiloa	EF157522	86
A. sagittae	cXil3818	L. Xiloa	EF157523	C
A. sagittae	cXil3819	L. Xiloa	EF157524	122
A. sagittae	cXil3821	L. Xiloa	EF157525	81
A. sagittae	cXil3825	L. Xiloa	EF157526	106
A. sagittae	cXil3826	L. Xiloa	EF157527	C
A. sagittae	cXil3831	L. Xiloa	EF157531	81
A. sagittae	cXil3833	L. Xiloa	EF157533	C
A. sagittae	cXil3834	L. Xiloa	EF157534	C
A. sagittae	cXil3838	L. Xiloa	EF157537	122
A. sagittae	cXil5016	L. Xiloa	HM183839	77
A. sagittae	cXil5017	L. Xiloa	HM184689	C
A. sagittae	cXil5018	L. Xiloa	HM184045	122
A. sagittae	cXil5019	L. Xiloa	HM184690	C
A. sagittae	cXil5020	L. Xiloa	HM184691	C
A. sagittae	cXil5034	L. Xiloa	HM184692	C
A. sagittae	cXil5035	L. Xiloa	HM183968	109
A. sagittae	cXil5036	L. Xiloa	HM184693	C
A. sagittae	cXil5038	L. Xiloa	HM184046	122
A. sagittae	cXil5055	L. Xiloa	HM184065	124
A. sagittae	cXil5056	L. Xiloa	HM184694	C
A. sagittae	cXil5106	L. Xiloa	HM184047	122
A. sagittae	cXil5107	L. Xiloa	HM184048	122
A. sagittae	cXil5108	L. Xiloa	HM184695	C
A. sagittae	cXil5111	L. Xiloa	HM184497	511
A. sagittae	cXil5187	L. Xiloa	HM184696	C
A. sagittae	cXil5200	L. Xiloa	HM183958	107
A. sagittae	cXil5216	L. Xiloa	HM183998	114
A. sagittae	cXil5226	L. Xiloa	HM184066	124
A. sagittae	cXil5265	L. Xiloa	HM184029	121
A. sagittae	cXil5266	L. Xiloa	HM184697	C
A. sagittae	cXil5267	L. Xiloa	HM184698	C
A. sagittae	cXil5268	L. Xiloa	HM184699	C
A. sagittae	cXil5269	L. Xiloa	HM184498	511
A. sagittae	cXil5270	L. Xiloa	HM184499	511
A. sagittae	cXil5271	L. Xiloa	HM184700	C
A. sagittae	cXil5272	L. Xiloa	HM184701	C
A. sagittae	cXil5273	L. Xiloa	HM184702	C
A. sagittae	cXil5282	L. Xiloa	HM184703	C

A. sagittae	cXil5283	L. Xiloa	HM184049	122
A. sagittae	cXil5284	L. Xiloa	HM184704	C
A. sagittae	cXil5285	L. Xiloa	HM184030	121
A. sagittae	cXil5286	L. Xiloa	HM184705	C
A. sagittae	cXil5287	L. Xiloa	HM184706	C
A. sagittae	cXil5288	L. Xiloa	HM184050	122
A. sagittae	cXil5289	L. Xiloa	HM184707	C
A. sagittae	cXil5290	L. Xiloa	HM184051	122
A. sagittae	cXil5292	L. Xiloa	HM184708	C
A. sagittae	cXil5293	L. Xiloa	HM183959	107
A. sagittae	cXil5295	L. Xiloa	HM183960	107
A. sagittae	cXil5296	L. Xiloa	HM183891	88
A. sagittae	cXil5297	L. Xiloa	HM183827	69
A. sagittae	cXil5299	L. Xiloa	HM184020	117
A. sagittae	cXil5301	L. Xiloa	HM184709	C
A. sagittae	cXil5302	L. Xiloa	HM184710	C
A. sagittae	cXil5304	L. Xiloa	HM184711	C
A. sagittae	cXil5323	L. Xiloa	HM184712	C
A. sagittae	cXil5324	L. Xiloa	HM183828	69
A. sagittae	cXil5450	L. Xiloa	HM184713	C
A. sagittae	cXil5453	L. Xiloa	HM183907	92
A. sagittae	cXil5455	L. Xiloa	HM183804	63
A. sagittae	cXil5472	L. Xiloa	HM183983	112
A. sagittae	cXil5473	L. Xiloa	HM184714	C
A. sagittae	cXil5489	L. Xiloa	HM184715	C
A. sagittae	cXil5538	L. Xiloa	HM183919	95
A. sagittae	cXil5539	L. Xiloa	HM184067	124
A. sagittae	cXil5540	L. Xiloa	HM184052	122
A. sagittae	cXil5541	L. Xiloa	HM184053	122
A. sagittae	cXil5542	L. Xiloa	HM183898	89
A. sagittae	cXil5543	L. Xiloa	HM184072	125
A. sagittae	cXil5544	L. Xiloa	HM184054	122
A. sagittae	cXil5545	L. Xiloa	HM184055	122
A. sagittae	cXil5546	L. Xiloa	HM184056	122
A. sagittae	cXil5547	L. Xiloa	HM184716	C
A. sagittae	cXil5548	L. Xiloa	HM184068	124
A. sagittae	cXil5729	L. Xiloa	HM184057	122
A. sagittae	cXil5730	L. Xiloa	HM184717	C
A. sagittae	cXil5731	L. Xiloa	HM184718	C
A. sagittae	cXil5734	L. Xiloa	HM183905	91
A. sagittae	cXil5735	L. Xiloa	HM184719	C
A. sagittae	cXil5736	L. Xiloa	HM184720	C
A. sagittae	cXil5737	L. Xiloa	HM184058	122
A. sagittae	cXil5741	L. Xiloa	HM184721	C
A. sagittae	cXil5743	L. Xiloa	HM184722	C
A. sagittae	cXil5744	L. Xiloa	HM183999	114
A. sagittae	cXil5750	L. Xiloa	HM184059	122
A. sagittae	cXil5751	L. Xiloa	HM184723	C
A. sagittae	cXil5752	L. Xiloa	HM184724	C
A. sagittae	cXil5753	L. Xiloa	HM184725	C
A. sagittae	cXil5754	L. Xiloa	HM184726	C
A. sagittae	cXil5755	L. Xiloa	HM183872	79
A. sagittae	cXil5756	L. Xiloa	HM184727	C

*A. sagittae**	cXil3765	L. Xiloa	EF157495	C
*A. sagittae**	cXil3766	L. Xiloa	EF157496	C
*A. sagittae**	cXil3767	L. Xiloa	EF157497	C
*A. sagittae**	cXil3768	L. Xiloa	EF157498	C
*A. sagittae**	cXil3773	L. Xiloa	EF157500	107
*A. sagittae**	cXil3774	L. Xiloa	EF157501	C
*A. sagittae**	cXil3809	L. Xiloa	EF157514	C
*A. sagittae**	cXil5044	L. Xiloa	HM183995	113
*A. sagittae**	cXil5054	L. Xiloa	HM184728	C
*A. sagittae**	cXil5102	L. Xiloa	HM183918	94
*A. sagittae**	cXil5451	L. Xiloa	HM183899	89
*A. sagittae**	cXil5452	L. Xiloa	HM184729	C
*A. sagittae**	cXil5732	L. Xiloa	HM184730	C
*A. sagittae**	cXil5733	L. Xiloa	HM184731	C
*A. sagittae**	cXil5738	L. Xiloa	HM184732	C
*A. sagittae**	cXil5739	L. Xiloa	HM184733	C
*A. sagittae**	cXil5740	L. Xiloa	HM184734	C
*A. sagittae**	cXil5832	L. Xiloa	HM184735	C
*A. sagittae**	cXil5833	L. Xiloa	HM184736	C
*A. sagittae**	cXil5834	L. Xiloa	HM184737	C
*A. sagittae**	cXil5835	L. Xiloa	HM184738	C
*A. sagittae**	cXil5836	L. Xiloa	HM184739	C
*A. sagittae**	cXil5837	L. Xiloa	HM184740	C
*A. sagittae**	cXil5838	L. Xiloa	HM184741	C
*A. sagittae**	cXil5894	L. Xiloa	HM184742	C
*A. sagittae**	cXil5895	L. Xiloa	HM184743	C
*A. sagittae**	cXil5896	L. Xiloa	HM184744	C
*A. sagittae**	cXil5897	L. Xiloa	HM184745	C
*A. sagittae**	cXil5898	L. Xiloa	HM184746	C
*A. sagittae**	cXil5899	L. Xiloa	HM184747	C
*A. sagittae**	cXil5900	L. Xiloa	HM184748	C
*A. sagittae**	cXil5901	L. Xiloa	HM184749	C
*A. sagittae**	cXil5902	L. Xiloa	HM184750	C
*A. sagittae**	cXil5903	L. Xiloa	HM184751	C
*A. sagittae**	cXil5904	L. Xiloa	HM184752	C
*A. sagittae**	cXil5905	L. Xiloa	HM184753	C
*A. sagittae**	cXil5906	L. Xiloa	HM184754	C
*A. sagittae**	cXil5907	L. Xiloa	HM184755	C
*A. sagittae**	cXil5908	L. Xiloa	HM184756	C
*A. sagittae**	cXil5909	L. Xiloa	HM184757	C
*A. sagittae**	cXil5910	L. Xiloa	HM184758	C
*A. sagittae**	cXil5911	L. Xiloa	HM183967	108
*A. sagittae**	cXil5912	L. Xiloa	HM184759	C
*A. sagittae**	cXil5913	L. Xiloa	HM183961	107
*A. sagittae**	cXil5914	L. Xiloa	HM184760	C
*A. sagittae**	cXil5915	L. Xiloa	HM184761	C
*A. sagittae**	cXil5916	L. Xiloa	HM184762	C
A. xiloaensis	cXil3088	L. Xiloa	EF157328	124
A. xiloaensis	cXil3096	L. Xiloa	EF157330	60
A. xiloaensis	cXil3098	L. Xiloa	EF157331	C
A. xiloaensis	cXil3570	L. Xiloa	EF157478	C
A. xiloaensis	cXil3572	L. Xiloa	EF157479	84
A. xiloaensis	cXil3573	L. Xiloa	EF157480	122

A. xiloaensis	cXil3574	L. Xiloa	EF157481	61
A. xiloaensis	cXil3582	L. Xiloa	EF157484	C
A. xiloaensis	cXil3583	L. Xiloa	EF157485	110
A. xiloaensis	cXil3586	L. Xiloa	EF157488	122
A. xiloaensis	cXil3840	L. Xiloa	EF157538	C
A. xiloaensis	cXil5026	L. Xiloa	HM183908	93
A. xiloaensis	cXil5027	L. Xiloa	HM183789	61
A. xiloaensis	cXil5028	L. Xiloa	HM184763	C
A. xiloaensis	cXil5029	L. Xiloa	HM183923	96
A. xiloaensis	cXil5030	L. Xiloa	HM183933	99
A. xiloaensis	cXil5032	L. Xiloa	HM183962	107
A. xiloaensis	cXil5041	L. Xiloa	HM184764	C
A. xiloaensis	cXil5057	L. Xiloa	HM183882	84
A. xiloaensis	cXil5058	L. Xiloa	HM183925	97
A. xiloaensis	cXil5076	L. Xiloa	HM183996	113
A. xiloaensis	cXil5077	L. Xiloa	HM184765	C
A. xiloaensis	cXil5104	L. Xiloa	HM183892	88
A. xiloaensis	cXil5105	L. Xiloa	HM184060	122
A. xiloaensis	cXil5109	L. Xiloa	HM184766	C
A. xiloaensis	cXil5110	L. Xiloa	HM183963	107
A. xiloaensis	cXil5178	L. Xiloa	HM184767	C
A. xiloaensis	cXil5182	L. Xiloa	HM183883	84
A. xiloaensis	cXil5189	L. Xiloa	HM183790	61
A. xiloaensis	cXil5194	L. Xiloa	HM184768	C
A. xiloaensis	cXil5205	L. Xiloa	HM184069	124
A. xiloaensis	cXil5210	L. Xiloa	HM183980	110
A. xiloaensis	cXil5215	L. Xiloa	HM183906	91
A. xiloaensis	cXil5217	L. Xiloa	HM184769	C
A. xiloaensis	cXil5274	L. Xiloa	HM184770	C
A. xiloaensis	cXil5275	L. Xiloa	HM183829	69
A. xiloaensis	cXil5276	L. Xiloa	HM183830	69
A. xiloaensis	cXil5277	L. Xiloa	HM184771	C
A. xiloaensis	cXil5278	L. Xiloa	HM184061	122
A. xiloaensis	cXil5279	L. Xiloa	HM184021	117
A. xiloaensis	cXil5280	L. Xiloa	HM183964	107
A. xiloaensis	cXil5281	L. Xiloa	HM183965	107
A. xiloaensis	cXil5294	L. Xiloa	HM183831	69
A. xiloaensis	cXil5298	L. Xiloa	HM183880	81
A. xiloaensis	cXil5300	L. Xiloa	HM184772	C
A. xiloaensis	cXil5303	L. Xiloa	HM184022	117
A. xiloaensis	cXil5305	L. Xiloa	HM184773	C
A. xiloaensis	cXil5325	L. Xiloa	HM183981	110
A. xiloaensis	cXil5327	L. Xiloa	HM183832	69
A. xiloaensis	cXil5448	L. Xiloa	HM184023	117
A. xiloaensis	cXil5449	L. Xiloa	HM184892	511
A. xiloaensis	cXil5454	L. Xiloa	HM183884	84
A. xiloaensis	cXil5468	L. Xiloa	HM183893	88
A. xiloaensis	cXil5469	L. Xiloa	HM183894	88
A. xiloaensis	cXil5471	L. Xiloa	HM183895	88
A. xiloaensis	cXil5474	L. Xiloa	HM183833	69
A. xiloaensis	cXil5477	L. Xiloa	HM183834	69
A. xiloaensis	cXil5478	L. Xiloa	HM184774	C
A. xiloaensis	cXil5479	L. Xiloa	HM183835	69

A. xiloaensis	cXil5481	L. Xiloa	HM184775	C
A. xiloaensis	cXil5482	L. Xiloa	HM184024	117
A. xiloaensis	cXil5484	L. Xiloa	HM184062	122
A. xiloaensis	cXil5485	L. Xiloa	HM183896	88
A. xiloaensis	cXil5488	L. Xiloa	HM184063	122
A. xiloaensis	cXil5500	L. Xiloa	HM183897	88
A. labiatus	lManMir4067	L. Managua	HM184151	189
A. labiatus	lManMir4068	L. Managua	HM184781	C
A. labiatus	lManMir4069	L. Managua	HM184152	190
A. labiatus	lManMir5001	L. Managua	HM184782	C
A. labiatus	lManMir5002	L. Managua	HM184783	C
A. labiatus	lManMir5249	L. Managua	HM184784	C
A. labiatus	lManMir5250	L. Managua	HM184785	C
A. labiatus	lManMir5251	L. Managua	HM184786	C
A. labiatus	lManMom5598	L. Managua	HM183947	106
A. labiatus	lManMom5599	L. Managua	HM184101	151
A. labiatus	lManMom5600	L. Managua	HM183873	79
A. labiatus	lManMom5601	L. Managua	HM184776	C
A. labiatus	lManMom5602	L. Managua		-
A. labiatus	lManMom5603	L. Managua	HM184102	152
A. labiatus	lManMom5604	L. Managua	HM183727	60
A. labiatus	lManMom5605	L. Managua	HM183791	61
A. labiatus	lManMom5606	L. Managua	HM184103	152
A. labiatus	lManMom5607	L. Managua	HM184086	144
A. labiatus	lManMom5814	L. Managua	HM184112	154
A. labiatus	lManMom5815	L. Managua	HM183909	93
A. labiatus	lManMom5816	L. Managua	HM184104	152
A. labiatus	lManMom5817	L. Managua		-
A. labiatus	lManMom5818	L. Managua		-
A. labiatus	lManMom5819	L. Managua	HM183910	93
A. labiatus	lManMom5820	L. Managua	HM184113	155
A. labiatus	lManMom5821	L. Managua	HM183948	106
A. labiatus	lManMom5822	L. Managua	HM184115	156
A. labiatus	lManMom5823	L. Managua	HM184777	C
A. labiatus	lManMom5824	L. Managua	HM183728	60
A. labiatus	lManMom5825	L. Managua	HM184105	152
A. labiatus	lManMom5826	L. Managua	HM184116	156
A. labiatus	lManMom5827	L. Managua	HM184778	C
A. labiatus	lManMom5828	L. Managua	HM184779	C
A. labiatus	lManMom5829	L. Managua	HM184780	C
A. labiatus	lManMom5830	L. Managua	HM184114	155
A. citrinellus	cManMom608	L. Managua	HM184108	153
A. citrinellus	cManMom831	L. Managua	HM184117	157
A. citrinellus	cManMir1178	L. Managua	AY567411	221
A. citrinellus	cManMir1179	L. Managua	AY567412	222
A. citrinellus	cManMir1180	L. Managua	AY567413	223
A. citrinellus	cManMir1181	L. Managua	AY567414	224
A. citrinellus	cManMir1182	L. Managua	AY567415	250
A. citrinellus	cManMir1183	L. Managua	AY567416	145
A. citrinellus	cManMir1184	L. Managua	AY567417	223
A. citrinellus	cManMir1185	L. Managua	AY567418	60
A. citrinellus	cManMir1186	L. Managua	AY567419	60

A. citrinellus	cManMir1187	L. Managua	AY567420	60
A. citrinellus	cManMir1188	L. Managua	AY567421	150
A. citrinellus	cManMir1189	L. Managua	AY567422	60
A. citrinellus	cManMir1190	L. Managua	AY567423	216
A. citrinellus	cManMir1191	L. Managua	AY567424	225
A. citrinellus	cManMir1192	L. Managua	AY567425	226
A. citrinellus	cManMir1193	L. Managua	AY567426	227
A. citrinellus	cManMir1194	L. Managua	AY567427	250
A. citrinellus	cManMir1195	L. Managua	AY567428	60
A. citrinellus	cManMir1196	L. Managua	AY567429	60
A. citrinellus	cManMir1197	L. Managua	AY567430	228
A. citrinellus	cManMir1198	L. Managua	AY567431	60
A. citrinellus	cManMir1199	L. Managua	AY567432	60
A. citrinellus	cManMir1200	L. Managua	AY567433	229
A. citrinellus	cManMir1201	L. Managua	AY567434	60
A. citrinellus	cManMir1202	L. Managua	AY567435	250
A. citrinellus	cManMir1203	L. Managua	AY567436	145
A. citrinellus	cManMir1204	L. Managua	AY567437	250
A. citrinellus	cManMir1205	L. Managua	AY567438	205
A. citrinellus	cManMir1206	L. Managua	AY567439	145
A. citrinellus	cManMir1207	L. Managua	AY567440	230
A. citrinellus	cManMir1208	L. Managua	AY567441	250
A. citrinellus	cManMir1209	L. Managua	AY567442	254
A. citrinellus	cManMir1210	L. Managua	AY567443	60
A. citrinellus	cManMir1211	L. Managua	AY567444	231
A. citrinellus	cManMir1212	L. Managua	AY567445	232
A. citrinellus	cManMir1213	L. Managua	AY567446	233
A. citrinellus	cManMir1214	L. Managua	AY567447	234
A. citrinellus	cManMir1215	L. Managua	AY567448	60
A. citrinellus	cManMir1216	L. Managua	AY567449	235
A. citrinellus	cManMir1217	L. Managua	AY567450	236
A. citrinellus	cManMir1218	L. Managua	AY567451	174
A. citrinellus	cManMir1219	L. Managua	AY567452	237
A. citrinellus	cManMir1220	L. Managua	AY567453	250
A. citrinellus	cManMir1221	L. Managua	AY567454	60
A. citrinellus	cManMir1222	L. Managua	AY567455	60
A. citrinellus	cManMir1223	L. Managua	AY567456	238
A. citrinellus	cManMir1224	L. Managua	AY567457	239
A. citrinellus	cManMir1225	L. Managua	AY567458	240
A. citrinellus	cManMir1226	L. Managua	AY567459	241
A. citrinellus	cManMir1227	L. Managua	AY567460	250
A. citrinellus	cManMir1228	L. Managua	AY567461	242
A. citrinellus	cManMir1229	L. Managua	AY567462	145
A. citrinellus	cManMir1230	L. Managua	AY567463	250
A. citrinellus	cManMir1231	L. Managua	AY567464	243
A. citrinellus	cManMir1232	L. Managua	AY567465	244
A. citrinellus	cManMir1233	L. Managua	AY567466	245
A. citrinellus	cManMir1234	L. Managua	AY567467	246
A. citrinellus	cManMir1235	L. Managua	AY567468	145
A. citrinellus	cManMir1236	L. Managua	AY567469	145
A. citrinellus	cManMir1237	L. Managua	AY567470	247
A. citrinellus	cManMir3001	L. Managua	HM184118	158
A. citrinellus	cManMir3002	L. Managua	HM184119	159

A. citrinellus	cManMir3003	L. Managua	HM184162	250
A. citrinellus	cManMir3004	L. Managua	HM184121	160
A. citrinellus	cManMir3006	L. Managua	HM184122	161
A. citrinellus	cManMir3007	L. Managua	HM184787	C
A. citrinellus	cManMir3008	L. Managua	HM184788	C
A. citrinellus	cManMir3009	L. Managua	HM183729	60
A. citrinellus	cManMir3010	L. Managua	HM184789	C
A. citrinellus	cManMir3011	L. Managua	HM184089	145
A. citrinellus	cManMir3012	L. Managua	HM184163	250
A. citrinellus	cManMir3013	L. Managua	HM184790	C
A. citrinellus	cManMir3014	L. Managua	HM184106	152
A. citrinellus	cManMir3015	L. Managua	HM184791	C
A. citrinellus	cManMir3016	L. Managua	HM184087	144
A. citrinellus	cManMir3017	L. Managua	HM184123	162
A. citrinellus	cManMir3018	L. Managua	HM183920	95
A. citrinellus	cManMir3019	L. Managua	HM183949	106
A. citrinellus	cManMir3020	L. Managua	HM184095	146
A. citrinellus	cManMir3021	L. Managua	HM184124	163
A. citrinellus	cManMir3022	L. Managua	HM183730	60
A. citrinellus	cManMir3023	L. Managua	HM184178	251
A. citrinellus	cManMir3024	L. Managua	HM183731	60
A. citrinellus	cManMir3025	L. Managua	HM184097	147
A. citrinellus	cManMir3026	L. Managua	HM184125	164
A. citrinellus	cManMir3027	L. Managua	HM184184	255
A. citrinellus	cManMir3028	L. Managua	HM184164	250
A. citrinellus	cManMir3029	L. Managua	HM184126	165
A. citrinellus	cManMir3030	L. Managua	HM183921	95
A. citrinellus	cManMir3031	L. Managua	HM184165	250
A. citrinellus	cManMir3032	L. Managua	HM184792	C
A. citrinellus	cManMir3033	L. Managua	HM184128	166
A. citrinellus	cManMir3034	L. Managua	HM184129	167
A. citrinellus	cManMir3035	L. Managua	HM184130	168
A. citrinellus	cManMir3036	L. Managua	HM183997	113
A. citrinellus	cManMir3037	L. Managua	HM183836	69
A. citrinellus	cManMir3038	L. Managua	HM184166	250
A. citrinellus	cManMir3039	L. Managua	HM184793	C
A. citrinellus	cManMir3040	L. Managua	HM183732	60
A. citrinellus	cManMir3041	L. Managua	HM184131	169
A. citrinellus	cManMir3042	L. Managua	HM184167	250
A. citrinellus	cManMir5333	L. Managua	HM184794	C
A. citrinellus	cManMir5334	L. Managua	HM184795	C
A. citrinellus	cManMir5335	L. Managua	HM183792	61
A. citrinellus	cManSFc1012	L. Managua	AY567307	63
A. citrinellus	cManSFc1013	L. Managua	AY567308	146
A. citrinellus	cManSFc1014	L. Managua	AY567309	C
A. citrinellus	cManSFc1015	L. Managua	AY567310	196
A. citrinellus	cManSFc1016	L. Managua		-
A. citrinellus	cManSFc1017	L. Managua		-
A. citrinellus	cManSFc1018	L. Managua		-
A. citrinellus	cManSFc1019	L. Managua	AY567311	197
A. citrinellus	cManSFc1020	L. Managua	AY567312	198
A. citrinellus	cManSFc1021	L. Managua	AY567313	250
A. citrinellus	cManSFc1022	L. Managua	AY567314	199

A. citrinellus	cManSFc1023	L. Managua	AY567315	200
A. citrinellus	cManSFc1024	L. Managua	AY567316	C
A. citrinellus	cManSFc1025	L. Managua		-
A. citrinellus	cManSFc1026	L. Managua		389
A. citrinellus	cManSFc1027	L. Managua	AY567317	201
A. citrinellus	cManSFc1028	L. Managua	AY567318	202
A. citrinellus	cManSFc1029	L. Managua		-
A. citrinellus	cManSFc1030	L. Managua		-
A. citrinellus	cManSFc1031	L. Managua		-
A. citrinellus	cManSFc1032	L. Managua	AY567319	107
A. citrinellus	cManSFc1033	L. Managua	AY567320	84
A. citrinellus	cManSFc1034	L. Managua	AY567321	C
A. citrinellus	cManSFc1035	L. Managua		-
A. citrinellus	cManSFc1036	L. Managua		-
A. citrinellus	cManSFc1037	L. Managua		-
A. citrinellus	cManSFc1038	L. Managua		-
A. citrinellus	cManSFc1039	L. Managua	AY567322	253
A. citrinellus	cManSFc1040	L. Managua	AY567323	61
A. citrinellus	cManSFc1041	L. Managua	AY567324	203
A. citrinellus	cManSFc1042	L. Managua		-
A. citrinellus	cManSFc1043	L. Managua		-
A. citrinellus	cManSFc1056	L. Managua	AY567325	152
A. citrinellus	cManSFc1057	L. Managua	AY567326	C
A. citrinellus	cManSFc1058	L. Managua		-
A. citrinellus	cManSFc1059	L. Managua	AY567327	C
A. citrinellus	cManSFc1060	L. Managua		-
A. citrinellus	cManSFc1061	L. Managua	AY567328	146
A. citrinellus	cManSFc1062	L. Managua	AY567329	145
A. citrinellus	cManSFc1063	L. Managua		-
A. citrinellus	cManSFc1064	L. Managua		-
A. citrinellus	cManSFc1065	L. Managua	AY567330	C
A. citrinellus	cManSFc1066	L. Managua		-
A. citrinellus	cManSFc1441	L. Managua		-
A. citrinellus	cManSFc1442	L. Managua	AY567331	C
A. citrinellus	cManSFc1443	L. Managua	AY567332	250
A. citrinellus	cManSFc1444	L. Managua	AY567333	C
A. citrinellus	cManSFc1445	L. Managua	AY567334	C
A. citrinellus	cManSFc1446	L. Managua	AY567335	183
A. citrinellus	cManSFc1447	L. Managua	AY567336	204
A. citrinellus	cManSFc1448	L. Managua	AY567337	250
A. citrinellus	cManSFc1449	L. Managua		-
A. citrinellus	cManSFc1450	L. Managua	AY567338	205
A. citrinellus	cManSFc1451	L. Managua	AY567339	54
A. citrinellus	cManSFc1452	L. Managua	AY567340	206
A. citrinellus	cManSFc1453	L. Managua	AY567341	207
A. citrinellus	cManSFc1454	L. Managua	AY567342	208
A. citrinellus	cManSFc1455	L. Managua	AY567343	C
A. citrinellus	cManSFc1456	L. Managua	AY567344	C
A. citrinellus	cManSFc1457	L. Managua	AY567345	108
A. citrinellus	cManSFc1458	L. Managua	AY567346	250
A. citrinellus	cManSFc1459	L. Managua		-
A. citrinellus	cManSFc1460	L. Managua	AY567347	209
A. citrinellus	cManSFc1461	L. Managua	AY567348	210

A. citrinellus	cManSFc1462	L. Managua		
A. citrinellus	cManSFc1463	L. Managua	AY567349	211
A. citrinellus	cManSFc1464	L. Managua		-
A. citrinellus	cManSFc1465	L. Managua	AY567350	205
A. citrinellus	cManSFc1466	L. Managua	AY567351	212
A. citrinellus	cManSFc1467	L. Managua	AY567352	213
A. citrinellus	cManSFc1468	L. Managua	AY567353	C
A. citrinellus	cManSFc1469	L. Managua	AY567354	214
A. citrinellus	cManSFc1470	L. Managua	AY567355	215
A. citrinellus	cManSFc1471	L. Managua	AY567356	C
A. citrinellus	cManSFc1472	L. Managua		-
A. citrinellus	cManSFc1473	L. Managua	AY567357	145
A. citrinellus	cManSFc1474	L. Managua	AY567358	C
A. citrinellus	cManSFc1475	L. Managua	AY567359	C
A. citrinellus	cManSFc1476	L. Managua	AY567360	174
A. citrinellus	cManSFc1477	L. Managua		-
A. citrinellus	cManSFc1478	L. Managua		-
A. citrinellus	cManSFc1479	L. Managua	AY567361	216
A. citrinellus	cManSFc1480	L. Managua		-
A. citrinellus	cManSFc1481	L. Managua	AY567362	C
A. citrinellus	cManSFc1482	L. Managua	AY567363	162
A. citrinellus	cManSFc1483	L. Managua		-
A. citrinellus	cManSFc1484	L. Managua		-
A. citrinellus	cManSFc1485	L. Managua		-
A. citrinellus	cManSFc1486	L. Managua		-
A. citrinellus	cManSFc1487	L. Managua	AY567364	77
A. citrinellus	cManSFc1488	L. Managua	AY567365	217
A. citrinellus	cManSFc1489	L. Managua	AY567366	C
A. citrinellus	cManSFc1490	L. Managua	AY567367	250
A. citrinellus	cManSFc1491	L. Managua	AY567368	C
A. citrinellus	cManSFc1492	L. Managua	AY567369	250
A. citrinellus	cManSFc1493	L. Managua	AY567370	218
A. citrinellus	cManSFc1494	L. Managua	AY567371	C
A. citrinellus	cManSFc1495	L. Managua		-
A. citrinellus	cManSFc1496	L. Managua	AY567372	219
A. citrinellus	cManSFc1497	L. Managua	AY567373	220
A. citrinellus	cManSFc1498	L. Managua		-
A. citrinellus	cManSFc1499	L. Managua		-
A. citrinellus	cManSFc1500	L. Managua	AY567374	145
A. citrinellus	cManSFc1501	L. Managua	AY567375	C
A. citrinellus	cManTip1437	L. Managua	HM184153	191
A. citrinellus	cManTip1438	L. Managua		-
A. citrinellus	cManTip1439	L. Managua	HM184154	192
A. citrinellus	cManTip1440	L. Managua	HM184796	C
A. citrinellus	cManTip3043	L. Managua	HM184797	C
A. citrinellus	cManTip3044	L. Managua	HM184090	145
A. citrinellus	cManTip3045	L. Managua	HM183805	63
A. citrinellus	cManTip3046	L. Managua	HM184798	C
A. citrinellus	cManTip3047	L. Managua	HM183733	60
A. citrinellus	cManTip3048	L. Managua	HM184799	C
A. citrinellus	cManTip3049	L. Managua	HM183950	106
A. citrinellus	cManTip3050	L. Managua	HM184168	250
A. citrinellus	cManTip3051	L. Managua	HM184132	170

A. citrinellus	cManTip3052	L. Managua	HM183734	60
A. citrinellus	cManTip3053	L. Managua	HM183735	60
A. citrinellus	cManTip3054	L. Managua	HM184133	171
A. citrinellus	cManTip3055	L. Managua	HM184134	172
A. citrinellus	cManTip3056	L. Managua	HM184800	C
A. citrinellus	cManTip3057	L. Managua	HM184135	173
A. citrinellus	cManTip3058	L. Managua	HM184801	C
A. citrinellus	cManTip3059	L. Managua	HM184091	145
A. citrinellus	cManTip3060	L. Managua	HM184136	174
A. citrinellus	cManTip3061	L. Managua	HM184169	250
A. citrinellus	cManTip3062	L. Managua	HM184137	175
A. citrinellus	cManTip3063	L. Managua	HM184138	176
A. citrinellus	cManTip3064	L. Managua	HM184802	C
A. citrinellus	cManTip3065	L. Managua		-
A. citrinellus	cManTip3066	L. Managua	HM183793	61
A. citrinellus	cManTip3067	L. Managua	HM184099	148
A. citrinellus	cManTip3068	L. Managua	HM184096	146
A. citrinellus	cManTip3069	L. Managua	HM183736	60
A. citrinellus	cManTip3070	L. Managua	HM184139	177
A. citrinellus	cManTip3071	L. Managua	HM184120	159
A. citrinellus	cManTip3072	L. Managua	HM184140	178
A. citrinellus	cManTip3073	L. Managua	HM183737	60
A. citrinellus	cManTip3074	L. Managua	HM184107	152
A. citrinellus	cManTip3075	L. Managua	HM184170	250
A. citrinellus	cManTip3076	L. Managua	HM184803	C
A. citrinellus	cManTip3077	L. Managua	HM184804	C
A. citrinellus	cManTip3078	L. Managua	HM184171	250
A. citrinellus	cManTip3079	L. Managua	HM184805	C
A. citrinellus	cManTip3080	L. Managua	HM183806	63
A. citrinellus	cManTip3081	L. Managua	HM184141	179
A. citrinellus	cManTip3082	L. Managua	HM184098	147
A. citrinellus	cManTip3083	L. Managua	HM183738	60
A. citrinellus	cManTip3084	L. Managua	HM183911	93
A. citrinellus	cManTip3085	L. Managua	HM184127	165
A. citrinellus	cManMat1001	L. Managua	HM184189	257
A. citrinellus	cManMat1002	L. Managua	HM184172	250
A. citrinellus	cManMat1003	L. Managua	HM184806	C
A. citrinellus	cManMat1004	L. Managua	HM184807	C
A. citrinellus	cManMat1005	L. Managua	HM184187	256
A. citrinellus	cManMat1006	L. Managua	HM184173	250
A. citrinellus	cManMat1007	L. Managua	HM184155	193
A. citrinellus	cManMat1008	L. Managua	HM184156	194
A. citrinellus	cManMat1009	L. Managua	HM184157	195
A. citrinellus	cManMat1010	L. Managua	HM184808	C
A. citrinellus	cManMat1011	L. Managua	HM183794	61
A. citrinellus	cManSAn3924	L. Managua	HM184092	145
A. citrinellus	cManSAn3925	L. Managua	HM184150	188
A. citrinellus	cManSAn3926	L. Managua	HM184174	250
A. citrinellus	cManSAn3927	L. Managua	HM184180	252
A. citrinellus	cManSAn3928	L. Managua	HM184158	248
A. citrinellus	cManSAn3929	L. Managua	HM184809	C
A. citrinellus	cManSAn3930	L. Managua	HM184142	180
A. citrinellus	cManSAn3931	L. Managua	HM184109	153

A. citrinellus	cManSAn3932	L. Managua	HM184143	181
A. citrinellus	cManSAn3933	L. Managua	HM184144	182
A. citrinellus	cManSAn3934	L. Managua	HM184145	183
A. citrinellus	cManSAn3935	L. Managua		-
A. citrinellus	cManSAn3936	L. Managua	HM184146	184
A. citrinellus	cManSAn3937	L. Managua	HM184810	C
A. citrinellus	cManSAn3938	L. Managua	HM184175	250
A. citrinellus	cManSAn3939	L. Managua	HM183739	60
A. citrinellus	cManSAn3940	L. Managua	HM184147	185
A. citrinellus	cManSAn3941	L. Managua	HM183740	60
A. citrinellus	cManSAn3942	L. Managua	HM184811	C
A. citrinellus	cManSAn3943	L. Managua	HM184812	C
A. citrinellus	cManSAn3944	L. Managua	HM183795	61
A. citrinellus	cManSAn3945	L. Managua	HM184110	153
A. citrinellus	cManSAn3946	L. Managua	HM184813	C
A. citrinellus	cManSAn3947	L. Managua	HM183741	60
A. citrinellus	cManSAn3948	L. Managua		-
A. citrinellus	cManSAn3949	L. Managua	HM184148	186
A. citrinellus	cManSAn3950	L. Managua	HM184814	C
A. citrinellus	cManSAn3951	L. Managua	HM184100	149
A. citrinellus	cManSAn3952	L. Managua	HM184149	187
A. citrinellus	cManSAn3953	L. Managua	HM184111	153
A. citrinellus	cManSAn3954	L. Managua	HM184190	257
A. citrinellus	cManSAn3955	L. Managua	HM183966	107
A. citrinellus	cManSAn3956	L. Managua	HM183807	63
A. citrinellus	cManSAn3957	L. Managua	HM183840	77
A. citrinellus	cManSAn3958	L. Managua	HM184088	144
A. citrinellus	cManSAn3959	L. Managua	HM184160	249
A. citrinellus	cTis1238	Tisma Pond	AY567266	135
A. citrinellus	cTis1239	Tisma Pond	AY567267	C
A. citrinellus	cTis1240	Tisma Pond	AY567268	95
A. citrinellus	cTis1241	Tisma Pond	AY567269	C
A. citrinellus	cTis1242	Tisma Pond	AY567270	60
A. citrinellus	cTis1243	Tisma Pond	AY567271	250
A. citrinellus	cTis1244	Tisma Pond	AY567272	C
A. citrinellus	cTis1245	Tisma Pond	AY567273	C
A. citrinellus	cTis1246	Tisma Pond	AY567274	C
A. citrinellus	cTis1247	Tisma Pond	AY567275	C
A. citrinellus	cTis1248	Tisma Pond	AY567276	C
A. citrinellus	cTis1249	Tisma Pond	AY567277	60
A. citrinellus	cTis1250	Tisma Pond	AY567278	C
A. citrinellus	cTis1251	Tisma Pond	AY567279	136
A. citrinellus	cTis1252	Tisma Pond	AY567280	137
A. citrinellus	cTis1253	Tisma Pond	AY567281	60
A. citrinellus	cTis1254	Tisma Pond	AY567282	C
A. citrinellus	cTis1255	Tisma Pond	AY567283	C
A. citrinellus	cTis1256	Tisma Pond	AY567284	138
A. citrinellus	cTis1257	Tisma Pond	AY567285	C
A. citrinellus	cTis1258	Tisma Pond	AY567286	149
A. citrinellus	cTis1259	Tisma Pond	AY567287	250
A. citrinellus	cTis1260	Tisma Pond		-
A. citrinellus	cTis1261	Tisma Pond	AY567288	147

A. citrinellus	cTis1262	Tisma Pond		-
A. citrinellus	cTis1263	Tisma Pond		-
A. citrinellus	cTis1264	Tisma Pond		-
A. citrinellus	cTis1265	Tisma Pond	AY567289	C
A. citrinellus	cTis1266	Tisma Pond	AY567290	139
A. citrinellus	cTis1267	Tisma Pond	AY567291	146
A. citrinellus	cTis1401	Tisma Pond	AY567292	C
A. citrinellus	cTis1402	Tisma Pond		-
A. citrinellus	cTis1403	Tisma Pond	AY567293	C
A. citrinellus	cTis1404	Tisma Pond	AY567294	140
A. citrinellus	cTis1405	Tisma Pond	AY567295	150
A. citrinellus	cTis1406	Tisma Pond	AY567296	148
A. citrinellus	cTis1407	Tisma Pond	AY567297	141
A. citrinellus	cTis1408	Tisma Pond	AY567298	142
A. citrinellus	cTis1409	Tisma Pond	AY567299	144
A. citrinellus	cTis1410	Tisma Pond	AY567300	250
A. citrinellus	cTis1411	Tisma Pond	AY567301	C
A. citrinellus	cTis1412	Tisma Pond	AY567302	250
A. citrinellus	cTis1413	Tisma Pond	AY567303	145
A. citrinellus	cTis1414	Tisma Pond	AY567304	143
A. citrinellus	cTis1415	Tisma Pond	AY567305	60
A. citrinellus	cTis1416	Tisma Pond	AY567306	257
A. citrinellus	cTis3843	Tisma Pond		-
A. citrinellus	cTis3844	Tisma Pond	HM184815	C
A. citrinellus	cTis3845	Tisma Pond		-
A. citrinellus	cTis3846	Tisma Pond	HM184081	130
A. citrinellus	cTis3847	Tisma Pond	HM183684	54
A. citrinellus	cTis3848	Tisma Pond	HM184176	250
A. citrinellus	cTis3911	Tisma Pond	HM184177	250
A. citrinellus	cTis3912	Tisma Pond	HM184816	C
A. citrinellus	cTis3913	Tisma Pond		-
A. citrinellus	cTis3914	Tisma Pond	HM183742	60
A. citrinellus	cTis3915	Tisma Pond	HM184082	131
A. citrinellus	cTis3916	Tisma Pond	HM184093	145
A. citrinellus	cTis3917	Tisma Pond	HM184094	145
A. citrinellus	cTis3918	Tisma Pond	HM184191	257
A. citrinellus	cTis3919	Tisma Pond	HM184083	132
A. citrinellus	cTis3920	Tisma Pond	HM184084	133
A. citrinellus	cTis3921	Tisma Pond	HM184085	134
A. citrinellus	cTis3922	Tisma Pond	HM183796	61
A. citrinellus	cTis3923	Tisma Pond		-
A. citrinellus	cNicIsl1044	L. Nicaragua	AY567011	60
A. citrinellus	cNicIsl1045	L. Nicaragua	AY567012	60
A. citrinellus	cNicIsl1046	L. Nicaragua	AY567013	C
A. citrinellus	cNicIsl1047	L. Nicaragua	AY567014	440
A. citrinellus	cNicIsl1048	L. Nicaragua	AY567015	441
A. citrinellus	cNicIsl1049	L. Nicaragua	AY567016	250
A. citrinellus	cNicIsl1050	L. Nicaragua	AY567017	C
A. citrinellus	cNicIsl1051	L. Nicaragua	AY567018	250
A. citrinellus	cNicIsl1052	L. Nicaragua	AY567019	250
A. citrinellus	cNicIsl1053	L. Nicaragua	AY567020	77
A. citrinellus	cNicIsl1054	L. Nicaragua	AY567021	C

A. citrinellus	cNicIsl1055	L. Nicaragua	AY567022	C
A. citrinellus	cNicIsl1067	L. Nicaragua	AY567023	442
A. citrinellus	cNicIsl1068	L. Nicaragua	AY567024	443
A. citrinellus	cNicIsl1069	L. Nicaragua	AY567025	444
A. citrinellus	cNicIsl1070	L. Nicaragua	AY567026	60
A. citrinellus	cNicIsl1071	L. Nicaragua	AY567027	60
A. citrinellus	cNicIsl1072	L. Nicaragua	AY567028	445
A. citrinellus	cNicIsl1073	L. Nicaragua	AY567029	446
A. citrinellus	cNicIsl1074	L. Nicaragua	AY567030	60
A. citrinellus	cNicIsl1075	L. Nicaragua	AY567031	289
A. citrinellus	cNicIsl1077	L. Nicaragua	AY567032	387
A. citrinellus	cNicIsl1079	L. Nicaragua	AY567033	390
A. citrinellus	cNicIsl1083	L. Nicaragua	AY567036	447
A. citrinellus	cNicIsl1084	L. Nicaragua	AY567037	60
A. citrinellus	cNicIsl1085	L. Nicaragua	AY567038	60
A. citrinellus	cNicIsl1087	L. Nicaragua	AY567039	390
A. citrinellus	cNicIsl1088	L. Nicaragua		-
A. citrinellus	cNicIsl1089	L. Nicaragua		-
A. citrinellus	cNicIsl1090	L. Nicaragua	AY567040	78
A. citrinellus	cNicIsl1091	L. Nicaragua	AY567041	60
A. citrinellus	cNicIsl1092	L. Nicaragua	AY567042	388
A. citrinellus	cNicIsl1093	L. Nicaragua	AY567043	286
A. citrinellus	cNicIsl1094	L. Nicaragua	AY567044	448
A. citrinellus	cNicIsl1095	L. Nicaragua	AY567045	449
A. citrinellus	cNicIsl1106	L. Nicaragua	AY567047	450
A. citrinellus	cNicIsl1108	L. Nicaragua	AY567049	451
A. citrinellus	cNicIsl1113	L. Nicaragua	AY567054	393
A. citrinellus	cNicIsl1116	L. Nicaragua	AY567057	328
A. citrinellus	cNicIsl1118	L. Nicaragua	AY567059	C
A. citrinellus	cNicIsl1119	L. Nicaragua	AY567060	121
A. citrinellus	cNicIsl1122	L. Nicaragua	AY567063	266
A. citrinellus	cNicIsl1124	L. Nicaragua	AY567065	458
A. citrinellus	cNicIsl1141	L. Nicaragua	AY567082	465
A. citrinellus	cNicIsl1150	L. Nicaragua	AY567091	467
A. citrinellus	cNicIsl1156	L. Nicaragua	AY567097	C
A. citrinellus	cNicIsl1162	L. Nicaragua	AY567103	113
A. citrinellus	cNicIsl1168	L. Nicaragua	AY567109	472
A. citrinellus	cNicIsl1169	L. Nicaragua	AY567110	473
A. citrinellus	cNicIsl1170	L. Nicaragua	AY567111	474
A. citrinellus	cNicIsl1171	L. Nicaragua	AY567112	66
A. citrinellus	cNicIsl1304	L. Nicaragua	AY567115	476
A. citrinellus	cNicIsl1306	L. Nicaragua	AY567117	477
A. citrinellus	cNicIsl1308	L. Nicaragua	AY567119	479
A. citrinellus	cNicIsl1323	L. Nicaragua	AY567134	391
A. citrinellus	cNicIsl1327	L. Nicaragua	AY567138	511
A. citrinellus	cNicIsl1335	L. Nicaragua	AY567146	106
A. citrinellus	cNicIsl1341	L. Nicaragua	AY567152	487
A. citrinellus	cNicIsl1348	L. Nicaragua	AY567159	C
A. citrinellus	cNicIsl3102	L. Nicaragua	EF157332	280
A. citrinellus	cNicIsl3103	L. Nicaragua		-
A. citrinellus	cNicIsl3104	L. Nicaragua	EF157333	254
A. citrinellus	cNicIsl3105	L. Nicaragua	EF157334	C
A. citrinellus	cNicIsl3106	L. Nicaragua	EF157335	C

A. citrinellus	cNicIsl3107	L. Nicaragua	EF157336	322
A. citrinellus	cNicIsl3108	L. Nicaragua	EF157337	323
A. citrinellus	cNicIsl3109	L. Nicaragua	EF157338	324
A. citrinellus	cNicIsl3110	L. Nicaragua	EF157339	60
A. citrinellus	cNicIsl3111	L. Nicaragua	EF157340	325
A. citrinellus	cNicIsl3112	L. Nicaragua	EF157341	66
A. citrinellus	cNicIsl3113	L. Nicaragua	EF157342	326
A. citrinellus	cNicIsl3114	L. Nicaragua	EF157343	C
A. citrinellus	cNicIsl3115	L. Nicaragua	EF157344	60
A. citrinellus	cNicIsl3116	L. Nicaragua	EF157345	60
A. citrinellus	cNicIsl3117	L. Nicaragua	EF157346	327
A. citrinellus	cNicIsl3299	L. Nicaragua	EF157395	60
A. citrinellus	cNicIsl3300	L. Nicaragua	EF157396	336
A. citrinellus	cNicIsl3301	L. Nicaragua	EF157397	337
A. citrinellus	cNicIsl3302	L. Nicaragua	EF157398	78
A. citrinellus	cNicIsl3303	L. Nicaragua	EF157399	338
A. citrinellus	cNicIsl3304	L. Nicaragua	EF157400	252
A. citrinellus	cNicIsl3305	L. Nicaragua	EF157401	69
A. citrinellus	cNicIsl3306	L. Nicaragua	EF157402	339
A. citrinellus	cNicIsl3307	L. Nicaragua	EF157403	60
A. citrinellus	cNicIsl3309	L. Nicaragua	EF157404	109
A. citrinellus	cNicIsl3310	L. Nicaragua	EF157405	340
A. citrinellus	cNicIsl3312	L. Nicaragua	EF157406	265
A. citrinellus	cNicIsl3314	L. Nicaragua	EF157407	342
A. citrinellus	cNicIsl3315	L. Nicaragua	EF157408	78
A. citrinellus	cNicIsl3328	L. Nicaragua	EF157409	345
A. citrinellus	cNicIsl3375	L. Nicaragua	EF157410	257
A. citrinellus	cNicIsl3376	L. Nicaragua	EF157411	357
A. citrinellus	cNicIsl3377	L. Nicaragua	EF157412	357
A. citrinellus	cNicIsl3378	L. Nicaragua	EF157413	358
A. citrinellus	cNicIsl3380	L. Nicaragua	EF157414	360
A. citrinellus	cNicIsl3385	L. Nicaragua	EF157415	361
A. citrinellus	cNicIsl3386	L. Nicaragua	EF157416	254
A. citrinellus	cNicIsl3387	L. Nicaragua		-
A. citrinellus	cNicIsl3397	L. Nicaragua	EF157417	C
A. citrinellus	cNicIsl3404	L. Nicaragua	EF157418	346
A. citrinellus	cNicIsl3406	L. Nicaragua	EF157419	254
A. citrinellus	cNicIsl3408	L. Nicaragua	EF157420	C
A. citrinellus	cNicIsl3409	L. Nicaragua	EF157421	365
A. citrinellus	cNicIsl3410	L. Nicaragua	EF157422	366
A. citrinellus	cNicIsl3414	L. Nicaragua	EF157423	368
A. citrinellus	cNicIsl3432	L. Nicaragua	EF157424	254
A. citrinellus	cNicIsl3438	L. Nicaragua	EF157425	60
A. citrinellus	cNicIsl5640	L. Nicaragua	HM183743	60
A. citrinellus	cNicIsl5641	L. Nicaragua	HM183744	60
A. citrinellus	cNicIsl5642	L. Nicaragua	HM183745	60
A. citrinellus	cNicIsl5643	L. Nicaragua	HM184817	C
A. citrinellus	cNicIsl5645	L. Nicaragua	HM184233	268
A. citrinellus	cNicIsl5646	L. Nicaragua	HM184818	C
A. citrinellus	cNicIsl5647	L. Nicaragua		-
A. citrinellus	cNicIsl5648	L. Nicaragua	HM183844	78
A. citrinellus	cNicIsl5649	L. Nicaragua	HM184819	C
A. citrinellus	cNicIsl5650	L. Nicaragua	HM184820	C

A. citrinellus	cNicIsl5651	L. Nicaragua		-
A. citrinellus	cNicIsl5652	L. Nicaragua	HM184239	273
A. citrinellus	cNicIsl5653	L. Nicaragua	HM184240	274
A. citrinellus	cNicIsl5654	L. Nicaragua	HM184241	275
A. citrinellus	cNicIsl5655	L. Nicaragua	HM184242	276
A. citrinellus	cNicIsl5656	L. Nicaragua	HM184243	277
A. citrinellus	cNicIsl5657	L. Nicaragua	HM184244	278
A. citrinellus	cNicIsl5658	L. Nicaragua	HM184245	279
A. citrinellus	cNicIsl5659	L. Nicaragua	HM183969	109
A. citrinellus	cNicIsl5660	L. Nicaragua	HM184246	280
A. citrinellus	cNicIsl5661	L. Nicaragua	HM184249	281
A. citrinellus	cNicIsl5662	L. Nicaragua	HM183845	78
A. labiatus	lNicIsl1080	L. Nicaragua	HM184382	375
A. labiatus	lNicIsl1081	L. Nicaragua	HM184821	C
A. labiatus	lNicIsl1082	L. Nicaragua		-
A. labiatus	lNicIsl1096	L. Nicaragua	HM184221	265
A. labiatus	lNicIsl1107	L. Nicaragua	HM183811	66
A. labiatus	lNicIsl1109	L. Nicaragua	HM184461	452
A. labiatus	lNicIsl1110	L. Nicaragua	HM183846	78
A. labiatus	lNicIsl1111	L. Nicaragua	AY567052	453
A. labiatus	lNicIsl1112	L. Nicaragua	HM183847	78
A. labiatus	lNicIsl1114	L. Nicaragua	HM184462	454
A. labiatus	lNicIsl1115	L. Nicaragua	HM184463	455
A. labiatus	lNicIsl1117	L. Nicaragua	HM184822	C
A. labiatus	lNicIsl1120	L. Nicaragua	HM184464	456
A. labiatus	lNicIsl1121	L. Nicaragua	HM184465	457
A. labiatus	lNicIsl1123	L. Nicaragua	HM184321	330
A. labiatus	lNicIsl1125	L. Nicaragua	HM184823	C
A. labiatus	lNicIsl1126	L. Nicaragua	HM184315	327
A. labiatus	lNicIsl1127	L. Nicaragua	HM184375	371
A. labiatus	lNicIsl1128	L. Nicaragua	HM184466	459
A. labiatus	lNicIsl1129	L. Nicaragua	HM184824	C
A. labiatus	lNicIsl1130	L. Nicaragua	HM184322	330
A. labiatus	lNicIsl1131	L. Nicaragua	HM184383	375
A. labiatus	lNicIsl1132	L. Nicaragua	HM183812	66
A. labiatus	lNicIsl1133	L. Nicaragua	HM184825	C
A. labiatus	lNicIsl1134	L. Nicaragua	HM184356	355
A. labiatus	lNicIsl1135	L. Nicaragua	HM184467	460
A. labiatus	lNicIsl1136	L. Nicaragua	HM184826	C
A. labiatus	lNicIsl1137	L. Nicaragua	HM184468	461
A. labiatus	lNicIsl1138	L. Nicaragua	HM184469	462
A. labiatus	lNicIsl1139	L. Nicaragua	HM184470	463
A. labiatus	lNicIsl1140	L. Nicaragua	HM184471	464
A. labiatus	lNicIsl1142	L. Nicaragua	HM184353	354
A. labiatus	lNicIsl1143	L. Nicaragua	HM184347	351
A. labiatus	lNicIsl1144	L. Nicaragua	HM184472	466
A. labiatus	lNicIsl1145	L. Nicaragua	HM184357	355
A. labiatus	lNicIsl1146	L. Nicaragua	HM184409	393
A. labiatus	lNicIsl1147	L. Nicaragua	HM184410	393
A. labiatus	lNicIsl1148	L. Nicaragua	HM184827	C
A. labiatus	lNicIsl1149	L. Nicaragua	HM183848	78
A. labiatus	lNicIsl1151	L. Nicaragua	HM183849	78
A. labiatus	lNicIsl1152	L. Nicaragua	HM184828	C

A. labiatus	lNicIsl1153	L. Nicaragua	HM184192	258
A. labiatus	lNicIsl1154	L. Nicaragua	HM184473	468
A. labiatus	lNicIsl1155	L. Nicaragua	HM184474	469
A. labiatus	lNicIsl1157	L. Nicaragua	HM184475	470
A. labiatus	lNicIsl1158	L. Nicaragua	HM184829	C
A. labiatus	lNicIsl1159	L. Nicaragua	HM184348	351
A. labiatus	lNicIsl1160	L. Nicaragua	HM183813	66
A. labiatus	lNicIsl1161	L. Nicaragua	HM184411	393
A. labiatus	lNicIsl1163	L. Nicaragua	HM184830	C
A. labiatus	lNicIsl1164	L. Nicaragua	HM184831	C
A. labiatus	lNicIsl1165	L. Nicaragua	HM184476	471
A. labiatus	lNicIsl1166	L. Nicaragua	HM183808	63
A. labiatus	lNicIsl1167	L. Nicaragua	HM184832	C
A. labiatus	lNicIsl1302	L. Nicaragua	HM184477	475
A. labiatus	lNicIsl1303	L. Nicaragua	HM184337	335
A. labiatus	lNicIsl1305	L. Nicaragua	HM183850	78
A. labiatus	lNicIsl1311	L. Nicaragua	HM183837	69
A. labiatus	lNicIsl1312	L. Nicaragua	HM184478	482
A. labiatus	lNicIsl1313	L. Nicaragua	HM183746	60
A. labiatus	lNicIsl1314	L. Nicaragua	HM184833	C
A. labiatus	lNicIsl1315	L. Nicaragua	HM184376	371
A. labiatus	lNicIsl1316	L. Nicaragua	HM184834	C
A. labiatus	lNicIsl1317	L. Nicaragua	HM184188	256
A. labiatus	lNicIsl1318	L. Nicaragua	HM184479	483
A. labiatus	lNicIsl1319	L. Nicaragua	HM184835	C
A. labiatus	lNicIsl1320	L. Nicaragua	HM183851	78
A. labiatus	lNicIsl1321	L. Nicaragua	HM184415	394
A. labiatus	lNicIsl1322	L. Nicaragua	HM184362	356
A. labiatus	lNicIsl1324	L. Nicaragua	HM184480	484
A. labiatus	lNicIsl1325	L. Nicaragua	HM184222	265
A. labiatus	lNicIsl1326	L. Nicaragua	HM184481	485
A. labiatus	lNicIsl1328	L. Nicaragua	HM184201	260
A. labiatus	lNicIsl1329	L. Nicaragua	HM184323	330
A. labiatus	lNicIsl1330	L. Nicaragua	HM184836	C
A. labiatus	lNicIsl1331	L. Nicaragua	HM184193	258
A. labiatus	lNicIsl1332	L. Nicaragua	HM184202	260
A. labiatus	lNicIsl1333	L. Nicaragua	HM184482	486
A. labiatus	lNicIsl1334	L. Nicaragua	HM184363	356
A. labiatus	lNicIsl1336	L. Nicaragua	HM184416	394
A. labiatus	lNicIsl1337	L. Nicaragua	HM184316	328
A. labiatus	lNicIsl1338	L. Nicaragua	HM184317	328
A. labiatus	lNicIsl1339	L. Nicaragua	HM184223	265
A. labiatus	lNicIsl1340	L. Nicaragua	HM184354	354
A. labiatus	lNicIsl1342	L. Nicaragua	HM184412	393
A. labiatus	lNicIsl1343	L. Nicaragua	HM184263	290
A. labiatus	lNicIsl1344	L. Nicaragua	HM184837	C
A. labiatus	lNicIsl1345	L. Nicaragua	HM184483	488
A. labiatus	lNicIsl1346	L. Nicaragua	HM184203	260
A. labiatus	lNicIsl1347	L. Nicaragua	HM184484	489
A. labiatus	lNicIsl1349	L. Nicaragua	HM184485	490
A. labiatus	lNicIsl1351	L. Nicaragua	HM184214	263
A. labiatus	lNicIsl1352	L. Nicaragua	HM184486	491
A. labiatus	lNicIsl1353	L. Nicaragua	HM184215	263

A. labiatus	lNicIsl1354	L. Nicaragua	HM184487	492
A. labiatus	lNicIsl1355	L. Nicaragua	HM184224	265
A. labiatus	lNicIsl1356	L. Nicaragua	HM184204	260
A. labiatus	lNicIsl1357	L. Nicaragua	HM184838	C
A. labiatus	lNicIsl1358	L. Nicaragua	HM184413	393
A. labiatus	lNicIsl1359	L. Nicaragua	HM184488	493
A. labiatus	lNicIsl1360	L. Nicaragua	HM184489	494
A. labiatus	lNicIsl1361	L. Nicaragua	HM184194	258
A. labiatus	lNicIsl1362	L. Nicaragua	HM184490	495
A. labiatus	lNicIsl1363	L. Nicaragua	HM184491	496
A. labiatus	lNicIsl1364	L. Nicaragua	HM184414	393
A. labiatus	lNicIsl3279	L. Nicaragua	HM184304	326
A. labiatus	lNicIsl3280	L. Nicaragua	HM184839	C
A. labiatus	lNicIsl3281	L. Nicaragua	HM184318	328
A. labiatus	lNicIsl3282	L. Nicaragua	HM184320	329
A. labiatus	lNicIsl3283	L. Nicaragua	HM184324	330
A. labiatus	lNicIsl3284	L. Nicaragua		-
A. labiatus	lNicIsl3285	L. Nicaragua	HM184329	331
A. labiatus	lNicIsl3286	L. Nicaragua	HM183838	69
A. labiatus	lNicIsl3287	L. Nicaragua	HM184330	332
A. labiatus	lNicIsl3288	L. Nicaragua	HM183852	78
A. labiatus	lNicIsl3289	L. Nicaragua	HM184840	C
A. labiatus	lNicIsl3290	L. Nicaragua	HM184305	326
A. labiatus	lNicIsl3291	L. Nicaragua	HM184841	C
A. labiatus	lNicIsl3292	L. Nicaragua	HM184842	C
A. labiatus	lNicIsl3293	L. Nicaragua	HM183853	78
A. labiatus	lNicIsl3294	L. Nicaragua	HM184331	333
A. labiatus	lNicIsl3295	L. Nicaragua	HM184843	C
A. labiatus	lNicIsl3296	L. Nicaragua	HM184334	334
A. labiatus	lNicIsl3297	L. Nicaragua	HM183854	78
A. labiatus	lNicIsl3298	L. Nicaragua	HM184338	335
A. labiatus	lNicIsl3308	L. Nicaragua		-
A. labiatus	lNicIsl3311	L. Nicaragua	HM184225	265
A. labiatus	lNicIsl3313	L. Nicaragua	HM183747	60
A. labiatus	lNicIsl3316	L. Nicaragua	HM184325	330
A. labiatus	lNicIsl3317	L. Nicaragua	HM183855	78
A. labiatus	lNicIsl3318	L. Nicaragua	HM184205	260
A. labiatus	lNicIsl3319	L. Nicaragua	HM184185	255
A. labiatus	lNicIsl3320	L. Nicaragua	HM183748	60
A. labiatus	lNicIsl3321	L. Nicaragua	HM184340	343
A. labiatus	lNicIsl3322	L. Nicaragua	HM184326	330
A. labiatus	lNicIsl3323	L. Nicaragua	HM184206	260
A. labiatus	lNicIsl3324	L. Nicaragua	HM184339	335
A. labiatus	lNicIsl3325	L. Nicaragua	HM184306	326
A. labiatus	lNicIsl3326	L. Nicaragua	HM184226	265
A. labiatus	lNicIsl3327	L. Nicaragua	HM184341	344
A. labiatus	lNicIsl3329	L. Nicaragua	HM184844	C
A. labiatus	lNicIsl3330	L. Nicaragua	HM184342	346
A. labiatus	lNicIsl3331	L. Nicaragua	HM184343	347
A. labiatus	lNicIsl3332	L. Nicaragua	HM184845	C
A. labiatus	lNicIsl3333	L. Nicaragua	HM184327	330
A. labiatus	lNicIsl3334	L. Nicaragua	HM184846	C
A. labiatus	lNicIsl3335	L. Nicaragua	HM184273	298

A. labiatus	lNicIsl3336	L. Nicaragua	HM184307	326
A. labiatus	lNicIsl3337	L. Nicaragua	HM184319	328
A. labiatus	lNicIsl3338	L. Nicaragua	HM184227	265
A. labiatus	lNicIsl3339	L. Nicaragua	HM184344	348
A. labiatus	lNicIsl3340	L. Nicaragua	HM184345	349
A. labiatus	lNicIsl3342	L. Nicaragua	HM184346	350
A. labiatus	lNicIsl3343	L. Nicaragua	HM184328	330
A. labiatus	lNicIsl3344	L. Nicaragua		-
A. labiatus	lNicIsl3345	L. Nicaragua	HM183814	66
A. labiatus	lNicIsl3346	L. Nicaragua	HM184332	333
A. labiatus	lNicIsl3347	L. Nicaragua		-
A. labiatus	lNicIsl3348	L. Nicaragua	HM183815	66
A. labiatus	lNicIsl3349	L. Nicaragua	HM184847	C
A. labiatus	lNicIsl3350	L. Nicaragua	HM184349	351
A. labiatus	lNicIsl3351	L. Nicaragua	HM184848	C
A. labiatus	lNicIsl3352	L. Nicaragua	HM184350	351
A. labiatus	lNicIsl3353	L. Nicaragua	HM184849	C
A. labiatus	lNicIsl3354	L. Nicaragua		512
A. labiatus	lNicIsl3355	L. Nicaragua	HM184351	352
A. labiatus	lNicIsl3356	L. Nicaragua	HM184850	C
A. labiatus	lNicIsl3357	L. Nicaragua	HM183816	66
A. labiatus	lNicIsl3358	L. Nicaragua	HM184352	353
A. labiatus	lNicIsl3359	L. Nicaragua	HM183856	78
A. labiatus	lNicIsl3360	L. Nicaragua		-
A. labiatus	lNicIsl3361	L. Nicaragua		-
A. labiatus	lNicIsl3362	L. Nicaragua	HM184308	326
A. labiatus	lNicIsl3363	L. Nicaragua	HM183817	66
A. labiatus	lNicIsl3364	L. Nicaragua		-
A. labiatus	lNicIsl3365	L. Nicaragua	HM184309	326
A. labiatus	lNicIsl3366	L. Nicaragua	HM184310	326
A. labiatus	lNicIsl3367	L. Nicaragua	HM184355	354
A. labiatus	lNicIsl3368	L. Nicaragua	HM183857	78
A. labiatus	lNicIsl3369	L. Nicaragua	HM184358	355
A. labiatus	lNicIsl3370	L. Nicaragua	HM183858	78
A. labiatus	lNicIsl3371	L. Nicaragua	HM183951	106
A. labiatus	lNicIsl3372	L. Nicaragua	HM184364	356
A. labiatus	lNicIsl3373	L. Nicaragua		-
A. labiatus	lNicIsl3374	L. Nicaragua	HM183859	78
A. labiatus	lNicIsl3379	L. Nicaragua	HM184368	359
A. labiatus	lNicIsl3381	L. Nicaragua	HM184851	C
A. labiatus	lNicIsl3382	L. Nicaragua	HM184852	C
A. labiatus	lNicIsl3383	L. Nicaragua	HM184207	260
A. labiatus	lNicIsl3384	L. Nicaragua	HM184228	265
A. labiatus	lNicIsl3388	L. Nicaragua		-
A. labiatus	lNicIsl3389	L. Nicaragua	HM184208	260
A. labiatus	lNicIsl3390	L. Nicaragua	HM183860	78
A. labiatus	lNicIsl3391	L. Nicaragua	HM184853	C
A. labiatus	lNicIsl3392	L. Nicaragua		-
A. labiatus	lNicIsl3393	L. Nicaragua	HM184369	362
A. labiatus	lNicIsl3394	L. Nicaragua	HM184854	C
A. labiatus	lNicIsl3395	L. Nicaragua	HM184855	C
A. labiatus	lNicIsl3396	L. Nicaragua		-
A. labiatus	lNicIsl3398	L. Nicaragua	HM184856	C

A. labiatus	lNicIsl3399	L. Nicaragua	HM183861	78
A. labiatus	lNicIsl3400	L. Nicaragua	HM184857	C
A. labiatus	lNicIsl3401	L. Nicaragua	HM184365	356
A. labiatus	lNicIsl3402	L. Nicaragua	HM183818	66
A. labiatus	lNicIsl3403	L. Nicaragua	HM184274	298
A. labiatus	lNicIsl3405	L. Nicaragua	HM184370	363
A. labiatus	lNicIsl3407	L. Nicaragua	HM184371	364
A. labiatus	lNicIsl3411	L. Nicaragua	HM184372	367
A. labiatus	lNicIsl3412	L. Nicaragua	HM184366	356
A. labiatus	lNicIsl3413	L. Nicaragua	HM184311	326
A. labiatus	lNicIsl3415	L. Nicaragua	HM184373	369
A. labiatus	lNicIsl3416	L. Nicaragua	HM184374	370
A. labiatus	lNicIsl3417	L. Nicaragua	HM183862	78
A. labiatus	lNicIsl3418	L. Nicaragua	HM184377	371
A. labiatus	lNicIsl3419	L. Nicaragua	HM184216	263
A. labiatus	lNicIsl3420	L. Nicaragua	HM184312	326
A. labiatus	lNicIsl3421	L. Nicaragua	HM184378	372
A. labiatus	lNicIsl3422	L. Nicaragua	HM184359	355
A. labiatus	lNicIsl3423	L. Nicaragua	HM184360	355
A. labiatus	lNicIsl3424	L. Nicaragua	HM184858	C
A. labiatus	lNicIsl3425	L. Nicaragua	HM184379	373
A. labiatus	lNicIsl3426	L. Nicaragua	HM184217	263
A. labiatus	lNicIsl3427	L. Nicaragua	HM184333	333
A. labiatus	lNicIsl3428	L. Nicaragua	HM183886	86
A. labiatus	lNicIsl3429	L. Nicaragua	HM184313	326
A. labiatus	lNicIsl3430	L. Nicaragua	HM184361	355
A. labiatus	lNicIsl3431	L. Nicaragua	HM184381	374
A. labiatus	lNicIsl3433	L. Nicaragua	HM184384	375
A. labiatus	lNicIsl3434	L. Nicaragua	HM184367	356
A. labiatus	lNicIsl3435	L. Nicaragua	HM184385	376
A. labiatus	lNicIsl3436	L. Nicaragua	HM183819	66
A. labiatus	lNicIsl3437	L. Nicaragua		-
A. labiatus	lNicIsl5609	L. Nicaragua	HM183863	78
A. labiatus	lNicIsl5610	L. Nicaragua	HM183749	60
A. labiatus	lNicIsl5611	L. Nicaragua	HM184195	258
A. labiatus	lNicIsl5612	L. Nicaragua	HM183864	78
A. labiatus	lNicIsl5613	L. Nicaragua	HM184197	259
A. labiatus	lNicIsl5614	L. Nicaragua	HM184209	260
A. labiatus	lNicIsl5615	L. Nicaragua	HM183750	60
A. labiatus	lNicIsl5616	L. Nicaragua	HM184212	261
A. labiatus	lNicIsl5617	L. Nicaragua	HM184213	262
A. labiatus	lNicIsl5618	L. Nicaragua		-
A. labiatus	lNicIsl5619	L. Nicaragua	HM184218	263
A. labiatus	lNicIsl5620	L. Nicaragua	HM183970	109
A. labiatus	lNicIsl5621	L. Nicaragua	HM184210	260
A. labiatus	lNicIsl5622	L. Nicaragua	HM183751	60
A. labiatus	lNicIsl5623	L. Nicaragua	HM184220	264
A. labiatus	lNicIsl5624	L. Nicaragua	HM183752	60
A. labiatus	lNicIsl5625	L. Nicaragua	HM183753	60
A. labiatus	lNicIsl5626	L. Nicaragua	HM184211	260
A. labiatus	lNicIsl5627	L. Nicaragua		-
A. labiatus	lNicIsl5628	L. Nicaragua		-
A. labiatus	lNicIsl5629	L. Nicaragua		-

A. labiatus	lNicIsl5630	L. Nicaragua	HM184859	C
A. labiatus	lNicIsl5631	L. Nicaragua	HM184229	265
A. labiatus	lNicIsl5632	L. Nicaragua	HM184860	C
A. labiatus	lNicIsl5633	L. Nicaragua		-
A. labiatus	lNicIsl5634	L. Nicaragua	HM184861	C
A. labiatus	lNicIsl5635	L. Nicaragua	HM183754	60
A. labiatus	lNicIsl5636	L. Nicaragua	HM184230	266
A. labiatus	lNicIsl5637	L. Nicaragua	HM183971	109
A. labiatus	lNicIsl5638	L. Nicaragua	HM184862	C
A. labiatus	lNicIsl5639	L. Nicaragua	HM184232	267
A. labiatus	lNicIsl5644	L. Nicaragua	HM184219	263
A. citrinellus	cNicOme1268	L. Nicaragua	AY567175	60
A. citrinellus	cNicOme1269	L. Nicaragua	AY567176	C
A. citrinellus	cNicOme1270	L. Nicaragua	AY567177	290
A. citrinellus	cNicOme1271	L. Nicaragua	AY567178	C
A. citrinellus	cNicOme1272	L. Nicaragua	AY567179	256
A. citrinellus	cNicOme1273	L. Nicaragua	AY567180	60
A. citrinellus	cNicOme1274	L. Nicaragua	AY567181	113
A. citrinellus	cNicOme1275	L. Nicaragua	AY567182	393
A. citrinellus	cNicOme1276	L. Nicaragua	AY567183	497
A. citrinellus	cNicOme1277	L. Nicaragua	AY567184	289
A. citrinellus	cNicOme1278	L. Nicaragua	AY567185	C
A. citrinellus	cNicOme1279	L. Nicaragua	AY567186	498
A. citrinellus	cNicOme1280	L. Nicaragua	AY567187	499
A. citrinellus	cNicOme1281	L. Nicaragua	AY567188	500
A. citrinellus	cNicOme1282	L. Nicaragua	AY567189	395
A. citrinellus	cNicOme1283	L. Nicaragua	AY567190	501
A. citrinellus	cNicOme1284	L. Nicaragua	AY567191	60
A. citrinellus	cNicOme1285	L. Nicaragua	AY567192	502
A. citrinellus	cNicOme1286	L. Nicaragua	AY567193	C
A. citrinellus	cNicOme1287	L. Nicaragua	AY567194	503
A. citrinellus	cNicOme1288	L. Nicaragua	AY567195	60
A. citrinellus	cNicOme1289	L. Nicaragua	AY567196	504
A. citrinellus	cNicOme1290	L. Nicaragua	AY567197	505
A. citrinellus	cNicOme1291	L. Nicaragua	AY567198	395
A. citrinellus	cNicOme1292	L. Nicaragua	AY567199	506
A. citrinellus	cNicOme1293	L. Nicaragua	AY567200	341
A. citrinellus	cNicOme1294	L. Nicaragua	AY567201	507
A. citrinellus	cNicOme1295	L. Nicaragua	AY567202	393
A. citrinellus	cNicOme1296	L. Nicaragua	AY567203	334
A. citrinellus	cNicOme1297	L. Nicaragua		-
A. citrinellus	cNicOme1298	L. Nicaragua	AY567204	C
A. citrinellus	cNicOme1299	L. Nicaragua	AY567205	508
A. citrinellus	cNicOme1300	L. Nicaragua	AY567206	C
A. citrinellus	cNicOme1301	L. Nicaragua	AY567207	509
A. citrinellus	cNicOme3982	L. Nicaragua		-
A. citrinellus	cNicOme3992	L. Nicaragua		-
A. citrinellus	cNicOme3993	L. Nicaragua		-
A. citrinellus	cNicOme3997	L. Nicaragua		-
A. citrinellus	cNicOme3998	L. Nicaragua		-
A. citrinellus	cNicOme4003	L. Nicaragua		-
A. citrinellus	cNicOme4004	L. Nicaragua	HM183755	60
A. citrinellus	cNicOme4005	L. Nicaragua	HM184447	426

A. citrinellus	cNicOme4006	L. Nicaragua		-
A. citrinellus	cNicOme4007	L. Nicaragua		-
A. citrinellus	cNicOme4009	L. Nicaragua		-
A. citrinellus	cNicOme4010	L. Nicaragua	HM184448	427
A. citrinellus	cNicOme4011	L. Nicaragua		-
A. citrinellus	cNicOme4012	L. Nicaragua		-
A. citrinellus	cNicOme4013	L. Nicaragua	HM184392	382
A. citrinellus	cNicOme4014	L. Nicaragua	HM183865	78
A. citrinellus	cNicOme4015	L. Nicaragua	HM184449	428
A. citrinellus	cNicOme4016	L. Nicaragua	HM184257	288
A. citrinellus	cNicOme4017	L. Nicaragua	HM184181	253
A. citrinellus	cNicOme4018	L. Nicaragua	HM183756	60
A. citrinellus	cNicOme4019	L. Nicaragua	HM184247	280
A. citrinellus	cNicOme4020	L. Nicaragua		-
A. citrinellus	cNicOme4021	L. Nicaragua	HM184405	390
A. citrinellus	cNicOme4022	L. Nicaragua		-
A. citrinellus	cNicOme4023	L. Nicaragua	HM184863	C
A. citrinellus	cNicOme4024	L. Nicaragua	HM183757	60
A. citrinellus	cNicOme4025	L. Nicaragua	HM184864	C
A. citrinellus	cNicOme4026	L. Nicaragua		-
A. citrinellus	cNicOme4027	L. Nicaragua	HM184298	317
A. citrinellus	cNicOme4028	L. Nicaragua	HM184161	249
A. citrinellus	cNicOme4029	L. Nicaragua	HM184865	C
A. citrinellus	cNicOme4030	L. Nicaragua	HM184159	248
A. citrinellus	cNicOme4031	L. Nicaragua	HM184450	429
A. citrinellus	cNicOme4032	L. Nicaragua	HM184451	430
A. citrinellus	cNicOme4033	L. Nicaragua	HM184452	431
A. citrinellus	cNicOme4034	L. Nicaragua	HM184453	432
A. citrinellus	cNicOme4035	L. Nicaragua	HM184454	433
A. citrinellus	cNicOme4036	L. Nicaragua	HM184455	434
A. citrinellus	cNicOme4037	L. Nicaragua	HM184456	435
A. citrinellus	cNicOme4038	L. Nicaragua	HM184398	387
A. citrinellus	cNicOme4039	L. Nicaragua	HM183922	95
A. citrinellus	cNicOme4040	L. Nicaragua	HM184457	436
A. citrinellus	cNicOme4041	L. Nicaragua	HM184458	437
A. citrinellus	cNicOme4042	L. Nicaragua	HM184459	438
A. citrinellus	cNicOme4043	L. Nicaragua	HM183810	65
A. citrinellus	cNicOme4044	L. Nicaragua	HM184460	439
A. citrinellus	cNicOme4045	L. Nicaragua	HM183797	61
A. citrinellus	cNicOme4046	L. Nicaragua	HM184335	334
A. citrinellus	cNicOme4047	L. Nicaragua	HM184336	334
A. citrinellus	cNicOme5758	L. Nicaragua	HM184250	282
A. citrinellus	cNicOme5759	L. Nicaragua	HM183758	60
A. citrinellus	cNicOme5760	L. Nicaragua	HM184251	283
A. citrinellus	cNicOme5761	L. Nicaragua	HM184866	C
A. citrinellus	cNicOme5762	L. Nicaragua	HM184252	284
A. citrinellus	cNicOme5763	L. Nicaragua	HM184253	285
A. citrinellus	cNicOme5764	L. Nicaragua	HM183759	60
A. citrinellus	cNicOme5765	L. Nicaragua	HM184867	C
A. citrinellus	cNicOme5766	L. Nicaragua	HM184255	286
A. citrinellus	cNicOme5767	L. Nicaragua	HM183760	60
A. citrinellus	cNicOme5768	L. Nicaragua	HM184868	C
A. citrinellus	cNicOme5769	L. Nicaragua	HM184256	287

A. citrinellus	cNicOme5770	L. Nicaragua	HM184869	C
A. citrinellus	cNicOme5771	L. Nicaragua	HM184179	251
A. citrinellus	cNicOme5772	L. Nicaragua	HM184258	288
A. citrinellus	cNicOme5773	L. Nicaragua	HM184260	289
A. citrinellus	cNicOme5774	L. Nicaragua	HM183761	60
A. citrinellus	cNicOme5775	L. Nicaragua	HM184870	C
A. citrinellus	cNicOme5776	L. Nicaragua	HM184264	290
A. citrinellus	cNicOme5777	L. Nicaragua	HM183762	60
A. citrinellus	cNicOme5778	L. Nicaragua	HM184265	291
A. citrinellus	cNicOme5779	L. Nicaragua	HM184267	292
A. citrinellus	cNicOme5780	L. Nicaragua	HM184268	293
A. citrinellus	cNicOme5781	L. Nicaragua	HM184269	294
A. citrinellus	cNicOme5782	L. Nicaragua	HM184270	295
A. citrinellus	cNicOme5783	L. Nicaragua	HM183866	78
A. citrinellus	cNicOme5784	L. Nicaragua	HM184271	296
A. citrinellus	cNicOme5785	L. Nicaragua	HM184272	297
A. citrinellus	cNicOme5786	L. Nicaragua	HM184275	298
A. citrinellus	cNicOme5787	L. Nicaragua	HM184277	299
A. citrinellus	cNicOme5788	L. Nicaragua	HM184278	300
A. citrinellus	cNicOme5794	L. Nicaragua	HM183809	64
A. citrinellus	cNicOme5795	L. Nicaragua	HM184283	303
A. citrinellus	cNicOme5796	L. Nicaragua	HM184284	303
A. citrinellus	cNicOme5797	L. Nicaragua	HM184871	C
A. citrinellus	cNicOme5798	L. Nicaragua	HM184279	300
A. citrinellus	cNicOme5799	L. Nicaragua	HM184285	304
A. citrinellus	cNicOme5800	L. Nicaragua	HM184286	305
A. citrinellus	cNicOme5801	L. Nicaragua	HM184287	306
A. citrinellus	cNicOme5802	L. Nicaragua	HM184288	307
A. citrinellus	cNicOme5803	L. Nicaragua	HM184872	C
A. citrinellus	cNicOme5804	L. Nicaragua	HM184266	291
A. citrinellus	cNicOme5808	L. Nicaragua	HM184289	308
A. citrinellus	cNicOme5809	L. Nicaragua	HM184186	255
A. citrinellus	cNicOme5810	L. Nicaragua	HM184290	309
A. citrinellus	cNicOme5811	L. Nicaragua	HM183763	60
A. citrinellus	cNicOme5812	L. Nicaragua	HM184291	310
A. citrinellus	cNicOme5813	L. Nicaragua	HM184873	C
A. labiatus	lNicOme4008	L. Nicaragua	HM184281	302
A. labiatus	lNicOme5757	L. Nicaragua	HM184182	254
A. labiatus	lNicOme5790	L. Nicaragua	HM183867	78
A. labiatus	lNicOme5791	L. Nicaragua	HM184280	301
A. labiatus	lNicOme5793	L. Nicaragua	HM184282	302
A. labiatus	lNicOme5805	L. Nicaragua	HM184874	C
A. labiatus	lNicOme5806	L. Nicaragua	HM184196	258
A. citrinellus	cNicRiv5847	L. Nicaragua	HM184231	266
A. citrinellus	cNicRiv5848	L. Nicaragua	HM184875	C
A. citrinellus	cNicRiv5849	L. Nicaragua	HM184296	315
A. citrinellus	cNicRiv5850	L. Nicaragua		-
A. citrinellus	cNicRiv5851	L. Nicaragua	HM184297	316
A. citrinellus	cNicRiv5852	L. Nicaragua	HM183764	60
A. citrinellus	cNicRiv5853	L. Nicaragua	HM183798	61
A. citrinellus	cNicRiv5854	L. Nicaragua	HM184876	C
A. citrinellus	cNicRiv5855	L. Nicaragua	HM184299	317
A. citrinellus	cNicRiv5856	L. Nicaragua	HM184300	318

A. citrinellus	cNicRiv5857	L. Nicaragua	HM184261	289
A. citrinellus	cNicRiv5858	L. Nicaragua	HM184301	319
A. citrinellus	cNicRiv5859	L. Nicaragua	HM184302	320
A. citrinellus	cNicRiv5860	L. Nicaragua	HM183868	78
A. citrinellus	cNicRiv5861	L. Nicaragua	HM183972	109
A. citrinellus	cNicRiv5862	L. Nicaragua	HM184303	321
A. citrinellus	cNicRiv5863	L. Nicaragua	HM183841	77
A. labiatus	lNicRiv5839	L. Nicaragua	HM184198	259
A. labiatus	lNicRiv5840	L. Nicaragua	HM184199	259
A. labiatus	lNicRiv5841	L. Nicaragua	HM184292	311
A. labiatus	lNicRiv5842	L. Nicaragua	HM184293	312
A. labiatus	lNicRiv5843	L. Nicaragua	HM184200	259
A. labiatus	lNicRiv5844	L. Nicaragua	HM184294	313
A. labiatus	lNicRiv5845	L. Nicaragua	HM184877	C
A. labiatus	lNicRiv5846	L. Nicaragua	HM184295	314
A. citrinellus	cNicSol3599	L. Nicaragua	HM184386	377
A. citrinellus	cNicSol3600	L. Nicaragua	HM184878	C
A. citrinellus	cNicSol3601	L. Nicaragua	HM184879	C
A. citrinellus	cNicSol3602	L. Nicaragua	HM184387	378
A. citrinellus	cNicSol3603	L. Nicaragua	HM184388	379
A. citrinellus	cNicSol3604	L. Nicaragua	HM184390	380
A. citrinellus	cNicSol3605	L. Nicaragua	HM184391	381
A. citrinellus	cNicSol3606	L. Nicaragua	HM184880	C
A. citrinellus	cNicSol3607	L. Nicaragua	HM184183	254
A. citrinellus	cNicSol3608	L. Nicaragua	HM184393	382
A. citrinellus	cNicSol3609	L. Nicaragua	HM184394	383
A. citrinellus	cNicSol3610	L. Nicaragua	HM184395	384
A. citrinellus	cNicSol3611	L. Nicaragua	HM184396	385
A. citrinellus	cNicSol3612	L. Nicaragua	HM184881	C
A. citrinellus	cNicSol3613	L. Nicaragua	HM184397	386
A. citrinellus	cNicSol3614	L. Nicaragua	HM184399	387
A. citrinellus	cNicSol3615	L. Nicaragua	HM184882	C
A. citrinellus	cNicSol3616	L. Nicaragua	HM184883	C
A. citrinellus	cNicSol3617	L. Nicaragua	HM184417	396
A. citrinellus	cNicSol3618	L. Nicaragua	HM184418	397
A. citrinellus	cNicSol3619	L. Nicaragua	HM183765	60
A. citrinellus	cNicSol3627	L. Nicaragua	HM184419	398
A. citrinellus	cNicSol3628	L. Nicaragua	HM184420	399
A. citrinellus	cNicSol3629	L. Nicaragua	HM184421	400
A. citrinellus	cNicSol3650	L. Nicaragua	HM184422	401
A. citrinellus	cNicSol3651	L. Nicaragua	HM183869	78
A. citrinellus	cNicSol3670	L. Nicaragua	HM183799	61
A. citrinellus	cNicSol3671	L. Nicaragua	HM184884	C
A. citrinellus	cNicSol3672	L. Nicaragua	HM184424	403
A. citrinellus	cNicSol3673	L. Nicaragua	HM183951	106
A. citrinellus	cNicSol3674	L. Nicaragua	HM184425	404
A. citrinellus	cNicSol3675	L. Nicaragua	HM184426	405
A. citrinellus	cNicSol3676	L. Nicaragua	HM184427	406
A. citrinellus	cNicSol3678	L. Nicaragua	HM183973	109
A. citrinellus	cNicSol3682	L. Nicaragua	HM184430	409
A. citrinellus	cNicSol3683	L. Nicaragua	HM184400	387
A. citrinellus	cNicSol3684	L. Nicaragua	HM184431	410
A. citrinellus	cNicSol3685	L. Nicaragua	HM184403	389

A. citrinellus	cNicSol3686	L. Nicaragua	HM183870	78
A. citrinellus	cNicSol3687	L. Nicaragua	HM184432	411
A. citrinellus	cNicSol3688	L. Nicaragua	HM184406	391
A. citrinellus	cNicSol3689	L. Nicaragua	HM183820	66
A. citrinellus	cNicSol3690	L. Nicaragua	HM184433	412
A. citrinellus	cNicSol3691	L. Nicaragua	HM184885	C
A. citrinellus	cNicSol3692	L. Nicaragua	HM184434	413
A. citrinellus	cNicSol3693	L. Nicaragua	HM183766	60
A. citrinellus	cNicSol3694	L. Nicaragua	HM183767	60
A. citrinellus	cNicSol3695	L. Nicaragua	HM184435	414
A. citrinellus	cNicSol3696	L. Nicaragua	HM184401	387
A. citrinellus	cNicSol3697	L. Nicaragua	HM183871	78
A. citrinellus	cNicSol3698	L. Nicaragua	HM184436	415
A. citrinellus	cNicSol3699	L. Nicaragua	HM184407	392
A. citrinellus	cNicSol3700	L. Nicaragua	HM184276	298
A. citrinellus	cNicSol3701	L. Nicaragua	HM184254	285
A. citrinellus	cNicSol3702	L. Nicaragua	HM184262	289
A. citrinellus	cNicSol3703	L. Nicaragua	HM184389	379
A. citrinellus	cNicSol3705	L. Nicaragua	HM184437	416
A. citrinellus	cNicSol3706	L. Nicaragua	HM184438	417
A. citrinellus	cNicSol3707	L. Nicaragua	HM183768	60
A. citrinellus	cNicSol3708	L. Nicaragua	HM183769	60
A. citrinellus	cNicSol3718	L. Nicaragua	HM183770	60
A. citrinellus	cNicSol3719	L. Nicaragua	HM183771	60
A. citrinellus	cNicSol3720	L. Nicaragua	HM184248	280
A. citrinellus	cNicSol3721	L. Nicaragua	HM183772	60
A. citrinellus	cNicSol3722	L. Nicaragua	HM184439	418
A. citrinellus	cNicSol3724	L. Nicaragua	HM184380	373
A. citrinellus	cNicSol3725	L. Nicaragua	HM184886	C
A. citrinellus	cNicSol3726	L. Nicaragua	HM184441	420
A. citrinellus	cNicSol3727	L. Nicaragua	HM184887	C
A. citrinellus	cNicSol3728	L. Nicaragua	HM183773	60
A. citrinellus	cNicSol3729	L. Nicaragua	HM184442	421
A. citrinellus	cNicSol3730	L. Nicaragua	HM183774	60
A. citrinellus	cNicSol3731	L. Nicaragua	HM184888	C
A. citrinellus	cNicSol3732	L. Nicaragua	HM184443	422
A. citrinellus	cNicSol3733	L. Nicaragua	HM184444	423
A. citrinellus	cNicSol3734	L. Nicaragua	HM184408	392
A. citrinellus	cNicSol3735	L. Nicaragua	HM184889	C
A. citrinellus	cNicSol3736	L. Nicaragua	HM184890	C
A. citrinellus	cNicSol3737	L. Nicaragua	HM183775	60
A. citrinellus	cNicSol3738	L. Nicaragua	HM184314	326
A. citrinellus	cNicSol3739	L. Nicaragua	HM184402	388
A. citrinellus	cNicSol3740	L. Nicaragua	HM183776	60
A. citrinellus	cNicSol3741	L. Nicaragua	HM184445	424
A. citrinellus	cNicSol3742	L. Nicaragua	HM184446	425
A. labiatus	lNicSol3668	L. Nicaragua	HM184423	402
A. labiatus	lNicSol3680	L. Nicaragua	HM184428	407
A. labiatus	lNicSol3681	L. Nicaragua	HM184429	408
A. labiatus	lNicSol3723	L. Nicaragua	HM184440	419
A. citrinellus	cSJuan3743	R. San Juan	HM184235	269
A. citrinellus	cSJuan3744	R. San Juan	HM184234	268

A. citrinellus	cSJuan3745	R. San Juan	HM184891	C
A. citrinellus	cSJuan3746	R. San Juan	HM184236	270
A. citrinellus	cSJuan3747	R. San Juan	HM184237	271
A. citrinellus	cSJuan3748	R. San Juan	HM183777	60
A. citrinellus	cSJuan3749	R. San Juan	HM184238	272
A. citrinellus	cSJuan3750	R. San Juan	HM184259	288
A. citrinellus	cCanoa3756	Las Canoas	HM184070	124
A. citrinellus	cCanoa3757	Las Canoas	HM184073	125
A. citrinellus	cCanoa3758	Las Canoas	HM184071	124
A. citrinellus	cCanoa3759	Las Canoas	HM184074	125
A. citrinellus	cCanoa3760	Las Canoas	HM184077	126
A. citrinellus	cCanoa3761	Las Canoas	HM184075	125
A. citrinellus	cCanoa3762	Las Canoas	HM184078	127
A. citrinellus	cCanoa3763	Las Canoas	HM184079	128
A. citrinellus	cCanoa3764	Las Canoas	HM184080	129
