# Supplementary material for: Phylogeography, colonization and population history of the Midas cichlid species complex (Amphilophus spp.) in the Nicaraguan crater lakes
Source: BMC Evol Biol. 2010 Oct 26;10:326. doi: 10.1186/1471-2148-10-326 (PMC3087546; doi:10.1186/1471-2148-10-326)

**Table S2** List of microsatellite loci used, with information about the repeat motif, primer sequences, annealing temperature (Ta) and reference where it has been published.

| Locus | Repeat motif | Primer sequence (5'-3') | Ta | Refference |
|---|---|---|---|---|
| Acit1 | $(AG)_{11}$ | F: AAA TGA GTT CAG CGA TGG CTG AG<br>R: TGC ACA TCA TGT CCG CCG AAC A | 49 | Noack *et al*. 2000 |
| Acit2 | $(GT)_{35}$ | F: GGC ACT GAG GAT TTA TAT TAC AGG<br>R: GAG GTC CAG CTG AGA ACA GGG | 52 | Noack *et al*. 2000 |
| Acit3 | $(GT)_{32}$ | F: CTT AAG GTG TAC CTG CTT AGC<br>R: GAG TGG GAA GAC AGA TGT TGA GG | 51 | Noack *et al*. 2000 |
| Acit4 | $(GT)_{22}$ | F: CCT TCC TAC TAG TTA GTC TTT CAC<br>R: CAC ATA GCA CAG TGC ATT CAC CC | 49 | Noack *et al*. 2000 |
| Acit6 | $(AGC)_{8}$ | F: GCC GCA CCC TCA TTA TCC TCA C<br>R: GTG ACT CCA ACG TGT AGC TTC C | 52 | Noack *et al*. 2000 |
| Unh002 | $(CA)_{23\text{-}43}$ | F: TTA TCC CAA CTT GCA ACT CTA TTT<br>R: TCC ATT TCC TGA TCT AAC GAC AAG | 52 | Kellog *et al.* 1995 |
| Unh011 | $(TG)_{15}$ | F: TTC TCT CCA ACA TCA GTG<br>R: CAC AGA TGA GAG CAG ACT T | 55 | Mckaye *et al.* 2002 |
| Unh012 | $(GT)_{39}$ | F: GCT GTA GCC CTC TTT AGT G<br>R: ACC ATG AAG ATA AAA CTT CA | 55 | Mckaye *et al.* 2002 |
| Unh013 | $(TG)_{34}$ | F: TTC TTA CAC ATG CCT CAC<br>R: AAC CGC TAT GAA CTT TCA | 55 | Mckaye *et al.* 2002 |
| TmoM7 | $(CA)AA(CA)_{10}$ | F: CTG CAG CCT CGC TCA CCA CGT AT<br>R: CAC CAG ATA ACT GCA CAG CCC AG | 48,50 | Zardoya *et al.* 1996 |
| Abur28 | $(AC)_{8}(CA)_{6}$ | F: CGAAGCGCTTTAGTGGTTTC<br>R: CATCGCTCAGCTTTCTCCTC | 55 | Sanetra *et al.* unpubl. |
| Abur45 | $(TG)_{43}$ | F: AGCAGTGGATGTGGCAGAG<br>R: TACAGGATGTGCCCCTCTC | 55 | Sanetra *et al.* unpubl. |
| Abur82 | $(CA)_{46}$ | F: ACAAAGAGCATGCACAAATG<br>R: CAGGAGACACAGTGGGAGATG | 55 | Sanetra *et al.* unpubl. |
| Abur151 | $(TG)_{25}$ | F: GTGAATGTGTGAGCGTGTCC<br>R: CAGATGCAAACAGCTCGAAG | 55 | Sanetra *et al.* unpubl. |
| BurtKit | $(TG)_{43}$ | F: GAT CTG GAG AAT ATG CAC CTG GA<br>R: ATC ACT CTT GTG GAT GGT TGG AG | 55 | Salzburger *et al*. unpubl. |

**Table S3** Results of the Bayesian inference of number of populations within the Midas Cichlid. Posterior probabilities are shown for two models, one that assumes a fixed number of populations (Pr[K=i]) and another that assumes a variable number of populations (Pr[K=i│X]), where X: Observations, K: Number of populations, Alpha: Parameter of Dirichlet process. **A.** Analysis including all populations. **B.** Analysis including only the populations in the large lakes.

**A.**

| i | Pr[K = i \| X] | Pr[K = i] |
|---|---|---|
| 1 | 0.0000 | 0.0017 |
| 2 | 0.0000 | 0.0118 |
| 3 | 0.0000 | 0.0393 |
| 4 | 0.9913 | 0.0849 |
| 5 | 0.0086 | 0.1344 |
| 6 | 0.0000 | 0.1666 |
| 7 | 0.0000 | 0.1689 |
| 8 | 0.0000 | 0.1442 |
| 9 | 0.0000 | 0.1059 |
| 10 | 0.0000 | 0.0681 |
| 11 | 0.0000 | 0.0388 |

E[K | X] = 4.0089
Var[K | X] = 0.0107
Alpha = 0.9018

**B.**

| i | Pr[K = i \| X] | Pr[K = i] |
|---|---|---|
| 1 | 0.0000 | 0.3614 |
| 2 | 0.9994 | 0.3747 |
| 3 | 0.0005 | 0.1869 |
| 4 | 0.0000 | 0.0600 |
| 5 | 0.0000 | 0.0140 |
| 6 | 0.0000 | 0.0026 |
| 7 | 0.0000 | 0.0004 |
| 8 | 0.0000 | 0.0000 |

E[K | X] = 2.0008
Var[K | X] = 0.0055
Alpha = 0.1580

**Table S4** Matrix of pairwise *F*-statistics between pairs of localities within **A.** Lake Managua and **B.** Lake Nicaragua. Below diagonal microsatellite data and above diagonal data on mtDNA sequences. Probability values: **P* < 0.05, ***P* < 0.001 (in black), ns, non significant (in red).

**A.**

| | | N | Momotombo *A. citrinellus* | Momotombo *A. labiatus* | Mateare | Miraflores *A. citrinellus* | Miraflores *A. labiatus* | Tipitapa | San Antonio | San Francisco | Tisma Pond |
|---|---|---|---|---|---|---|---|---|---|---|---|
| **Momotombo** | ***A. citrinellus*** | 2 | **0.000** | 0.044$^{ns}$ | 0.044$^{ns}$ | -0.007$^{ns}$ | 0.053$^{ns}$ | 0.020$^{ns}$ | 0.006$^{ns}$ | -0.002$^{ns}$ | 0.003$^{ns}$ |
| | ***A. labiatus*** | 27 | 0.047$^{ns}$ | **0.000** | 0.026* | 0.035** | 0.035* | 0.033** | 0.026** | 0.024** | 0.029** |
| **Mateare** | | 11 | 0.012$^{ns}$ | 0.193** | **0.000** | 0.009$^{ns}$ | 0.045** | 0.012$^{ns}$ | 0.015$^{ns}$ | 0.001$^{ns}$ | 0.004$^{ns}$ |
| **Miraflores** | ***A. citrinellus*** | 104 | 0.122$^{ns}$ | 0.245** | 0.004$^{ns}$ | **0.000** | 0.008$^{ns}$ | 0.001$^{ns}$ | 0.008$^{ns}$ | -0.001$^{ns}$ | -0.003$^{ns}$ |
| | ***A. labiatus*** | 8 | 0.039$^{ns}$ | -0.002$^{ns}$ | 0.097$^{ns}$ | 0.195** | **0.000** | 0.001$^{ns}$ | 0.009$^{ns}$ | 0.006$^{ns}$ | 0.001$^{ns}$ |
| **Tipitapa** | | 47 | 0.008$^{ns}$ | 0.138** | -0.029$^{ns}$ | 0.025$^{ns}$ | 0.079$^{ns}$ | **0.000** | 0.004$^{ns}$ | 0.002$^{ns}$ | 0.0004$^{ns}$ |
| **San Antonio** | | 36 | -0.106$^{ns}$ | 0.040* | 0.045* | 0.143** | -0.012$^{ns}$ | 0.041$^{ns}$ | **0.000** | -0.001$^{ns}$ | 0.002$^{ns}$ |
| **San Francisco** | | 104 | 0.015$^{ns}$ | 0.136** | -0.033$^{ns}$ | 0.028* | 0.075$^{ns}$ | -0.012$^{ns}$ | 0.051** | **0.000** | -0.001$^{ns}$ |
| **Tisma Pond** | | 65 | 0.004$^{ns}$ | 0.148** | -0.034$^{ns}$ | 0.031* | 0.082* | -0.012$^{ns}$ | 0.052* | -0.011$^{ns}$ | **0.000** |

**B.**

| | | N | Isletas *A. citrinellus* | Isletas *A. labiatus* | Ometepe *A. citrinellus* | Ometepe *A. labiatus* | Rivas *A. citrinellus* | Rivas *A. labiatus* | Solentiname *A. citrinellus* | Solentiname *A. labiatus* | River San Juan | Las Canoas | Tisma Pond |
|---|---|---|---|---|---|---|---|---|---|---|---|---|---|
| **Isletas** | ***A. citrinellus*** | 129 | **0.000** | 0.010** | 0.003** | 0.020** | 0.009$^{ns}$ | 0.033** | 0.010** | 0.016$^{ns}$ | 0.011$^{ns}$ | 0.061** | 0.034** |
| | ***A. labiatus*** | 264 | 0.011** | **0.000** | 0.010** | 0.021$^{ns}$ | 0.012** | 0.026** | 0.016** | 0.030* | 0.018** | 0.078** | 0.035** |
| **Ometepe** | ***A. citrinellus*** | 131 | -0.001$^{ns}$ | 0.020** | **0.000** | 0.024** | 0.007$^{ns}$ | 0.033** | 0.008** | 0.027* | 0.006$^{ns}$ | 0.068** | 0.039** |
| | ***A. labiatus*** | 7 | 0.027$^{ns}$ | 0.029$^{ns}$ | 0.052* | **0.000** | 0.033** | 0.052* | 0.032** | 0.013$^{ns}$ | 0.035** | 0.131** | 0.024* |
| **Rivas** | ***A. citrinellus*** | 17 | 0.005$^{ns}$ | 0.016$^{ns}$ | 0.010$^{ns}$ | 0.015$^{ns}$ | **0.000** | 0.016$^{ns}$ | 0.022** | 0.044* | 0.020$^{ns}$ | 0.107** | 0.022* |
| | ***A. labiatus*** | 8 | 0.028$^{ns}$ | 0.034$^{ns}$ | 0.036$^{ns}$ | 0.078$^{ns}$ | 0.037$^{ns}$ | **0.000** | 0.047** | 0.090** | 0.056** | 0.175** | 0.036** |
| **Solentiname** | ***A. citrinellus*** | 84 | -0.003$^{ns}$ | 0.017** | -0.004$^{ns}$ | 0.054$^{ns}$ | 0.016$^{ns}$ | 0.039$^{ns}$ | **0.000** | 0.020$^{ns}$ | 0.009$^{ns}$ | 0.071** | 0.044** |
| | ***A. labiatus*** | 4 | 0.114$^{ns}$ | 0.117$^{ns}$ | 0.148$^{ns}$ | 0.022$^{ns}$ | -0.001$^{ns}$ | 0.165$^{ns}$ | 0.018* | **0.000** | 0.025* | 0.162* | 0.072** |
| **River San Juan** | | 8 | -0.010$^{ns}$ | 0.017$^{ns}$ | -0.017$^{ns}$ | 0.024$^{ns}$ | 0.007$^{ns}$ | 0.005$^{ns}$ | -0.005$^{ns}$ | 0.117$^{ns}$ | **0.000** | 0.051** | 0.050** |
| **Las Canoas** | | 9 | 0.411** | 0.407** | 0.451** | 0.408** | 0.330** | 0.545** | 0.467** | 0.196* | 0.354** | **0.000** | 0.118** |
| **Tisma Pond** | | 65 | 0.107** | 0.130** | 0.119** | 0.083$^{ns}$ | 0.079* | 0.159* | 0.128** | 0.097$^{ns}$ | 0.110$^{ns}$ | 0.525** | **0.000** |

**Figure S1** Plot of log-likelihoods resulting from the clustering analysis with STRUCTURE. A. Analysis including all populations (10 replicates). B. Analysis including only the populations in the large lakes (5 replicates).

**A.**

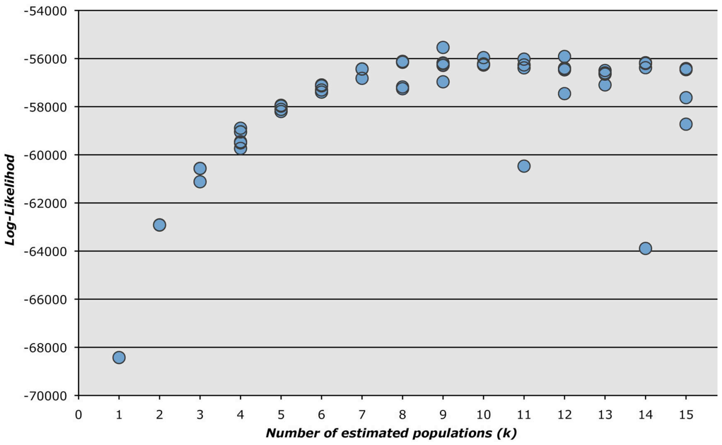


**B.**

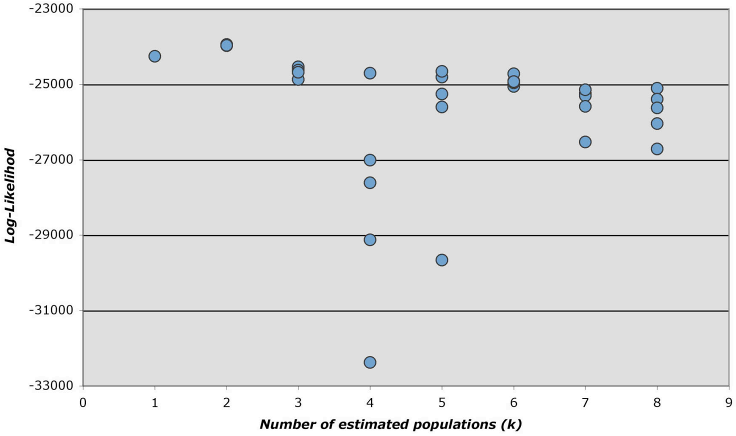

**Figure S2** Bayesian clustering analysis with the software STRUCTURE based on 15 microsatellites on the samples from the large lakes Managua and Nicaragua, and Tisma Pond. (7,8,9 are Managua *A. citrinellus*, Managua *A. labiatus*, and Tisma; 10, 12 Nicaragua *A. citrinellus* and *A. labiatus*).

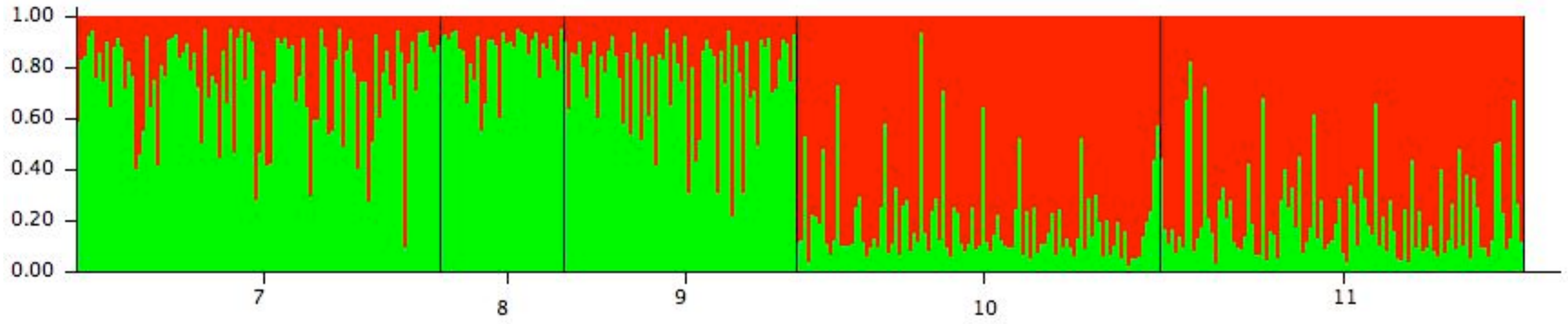

Supplement: Additional file 2 — Supplementary materials. This additional file contains information about the microsatellite primers used in the study, and additional details on the results of the clustering analyses with STRUCTURE and STRUCTURAMA, and pairwise statistics. [file 1471-2148-10-326-S2.PDF]
